# Surgical Risk Factors for Ischemic Stroke Following Coronary Artery Bypass Grafting. A Multi-Factor Multimodel Analysis

**DOI:** 10.3389/fcvm.2021.622480

**Published:** 2021-07-05

**Authors:** Sandro Gelsomino, Cecilia Tetta, Francesco Matteucci, Stefano del Pace, Orlando Parise, Edvin Prifti, Aleksander Dokollari, Gianmarco Parise, Linda Renata Micali, Mark La Meir, Massimo Bonacchi

**Affiliations:** ^1^Cardiovascular Research Institute Maastricht - CARIM, Maastricht University Medical Centre, Maastricht, Netherlands; ^2^Cardiac Surgery Department, Vrije Universiteit Brussels, Brussels, Belgium; ^3^Cardio-Thoraco-Vascular Department, Careggi Hospital, Florenze, Italy; ^4^Division of Cardiac Surgery, University Hospital Center of Tirana, Tirana, Albania; ^5^Cardiac Surgery, St. Michael's Hospital, University of Toronto, Toronto, ON, Canada; ^6^Cardiac Surgery Unit, Department of Experimental and Clinical Medicine, University of Florence, Firenze, Italy

**Keywords:** stroke, coronary artery bypass—adverse effects, surgical technique, aortic manipulation, aortic cannulation, off-pump artery bypass and grafting, proximal aortic anastomosis

## Abstract

**Background:** Ischemic stroke after coronary artery bypass (CABG) has been often linked to aortic manipulation during surgery.

**Objectives:** The objective of the study was to estimate the rate of postoperative ischemic stroke within 30 days from CABG by surgical risk factors alone or in combination.

**Methods:** The multinomial propensity score for multiple treatments was used to create six models with a total of 16,255 consecutive patients undergoing isolated CABG. For each model, a different classification variable was used to stratify patients.

**Results:** Balance achieved in all models was substantial, enabling unbiased estimation of the treatment estimand. Both off-pump techniques with (0.009; 95% CI 0.006–0.011) or without proximal anastomoses (0.005; 0.005–0.003), and surgery performed on the beating heart using cardiopulmonary bypass with (0.009; 0.006–0.011) or without proximal anastomoses (0.024; 0.021–0.029) showed a mean stroke estimate significantly lower than the other techniques. Off-pump surgery and on-pump surgery without an aortic cross-clamp yielded nearly equal incidences of stroke (0.012; 0.008–0.015 and 0.018; 0.012–0.023, respectively). Using an aortic cross-clamp significantly increased the stroke estimate (0.075; 0.061–0.088), whereas using a side-biting clamp did not (0.039; 0.033–0.044). The number of aortic touches (0.029; 0.026–0.031) and the number of proximal anastomoses (0.044; 0.035–0.047) did not significantly increase the incidence of stroke.

**Conclusions:** Aortic cross-clamping was found to be the primary cause of post-CABG ischemic stroke. Instead, additional aortic manipulation from a side-biting clamp, on-pump surgery, multiple aortic touches, number of proximal anastomoses, and aortic cannulation were found not to increase the estimate of stroke significantly. Further research on this topic is warranted.

## Graphical Abstract

Factors increasing post CABG incidence of ischemic stroke (red) and those which were not significant at the multi-model multifactorial analysis that analyzed all technical factors alone or in different combinations (green). 
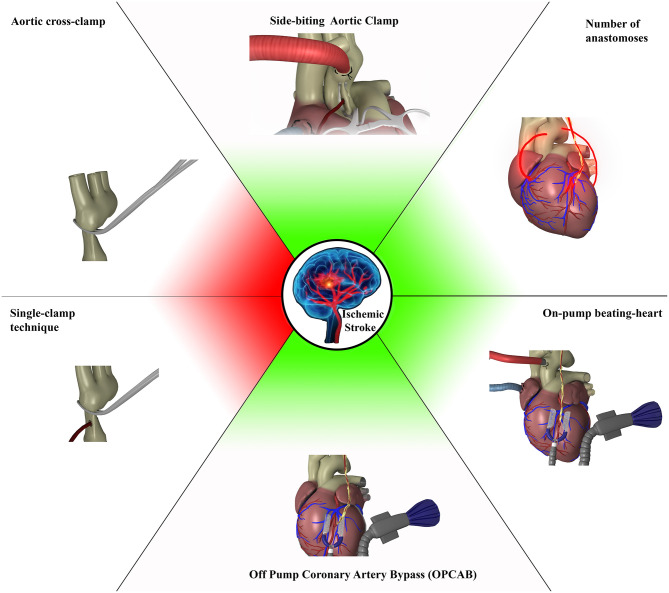

## Introduction

Ischemic stroke following coronary artery bypass grafting (CABG) has been related to aortic manipulation, which may trigger the embolization of debris (1). Several strategies have been developed to minimize the risk of ischemic strokes, such as the single clamp technique (2), the “ no-touch-anaortic” procedure (3, 4), and the use of devices for creating proximal anastomoses (4–6). Also, other authors have found that off-pump coronary artery bypass (OPCAB) lessens the incidence of stroke (7), although this finding is still the subject of heated debates (8, 9).

However, despite the abundance of studies, most published data are the result of one-to-one comparisons of only a few of all the available techniques (10, 11), ruling out other potential causes of ischemic stroke such as the aortic cannula, aortic needle, etc. Moreover, most of the studies were biased by significant baseline imbalances that may have influenced the study outcomes (12).

Using a large patient population undergoing CABG, this study explored whether different surgical procedures encompassing vary degrees of aortic manipulation may have affected the incidence of ischemic stroke.

## Methods

### Patient Classification

The approval for this retrospective study was according to National Laws regulating observational retrospective studies.

The study population included 16,255 consecutive patients undergoing isolated CABG between 1997 and 2017 at our Institutions.

**Figure 1:**
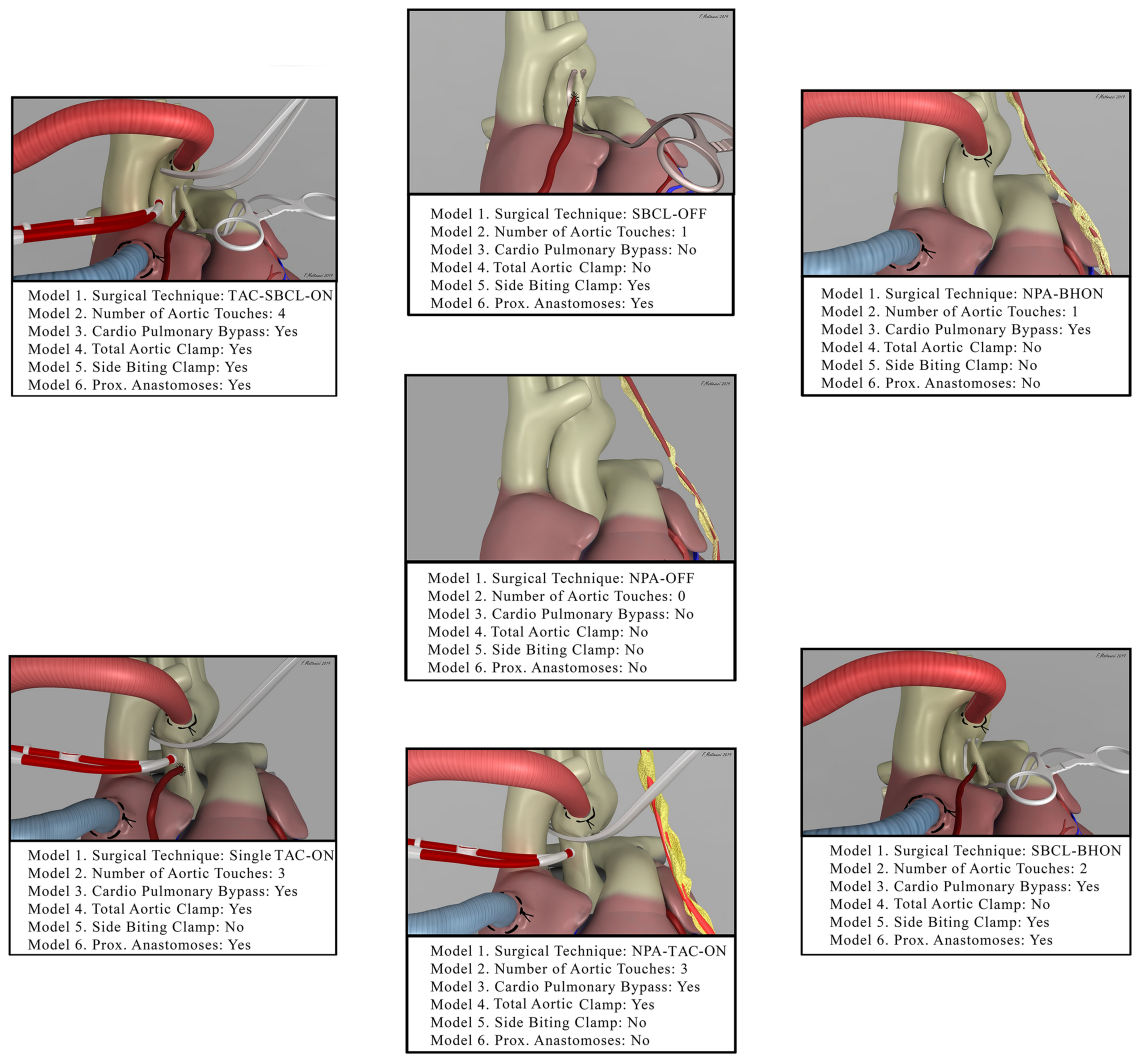
The picture shows the seven combinations of surgical techniques for coronary artery bypass grafting (Model 1, from the middle image in the clockwise direction). It also shows how each of these techniques was included in the other models. NPA-OFF, No proximal Anastomoses-Off pump; SBCL-OFF, Side Biting Clamp-Off Pump; NPA-BHON, No Proximal Anastomoses-Beating Heart-On Pump; SBCL-BHON, Side Biting Clamp-Beating Heart-On-Pump; NPA-TAC-ON, No Proximal Anastomoses- Total Aortic Clamp-On-Pump; Single TAC-ON, Single Total Aortic Clamp-On Pump; TAC-SBCL-ON, Total Aortic Clamp-Side Biting Clamp-On Pump.

Patients were classified as follows by creating six propensity score models (Figure 1).

*Model 1—Patients stratified by surgical technique:* (1) Off-pump technique (OPCAB) without proximal anastomoses on the aorta (NPA-Off); (2) OPCAB with proximal anastomoses on the aorta performed with the use of a side-biting clamp (SBCL-Off); (3) surgery performed on the beating heart but with the use of cardiopulmonary bypass without an aortic clamp, cardioplegic arrest, and proximal anastomoses (NPA-BHON); (4) surgery performed on the beating heart but on-pump with proximal anastomoses on the aorta made with the use of a side-biting clamp (SBCL-BHON); (5) on-pump technique without proximal anastomoses on the aorta, obtained using one or two internal thoracic arteries (ITAs) and/or Y graft anastomosed to ITAs (NPA-TAC-ON); (6) on-pump technique with proximal anastomoses performed without releasing the aortic clamp on cardiopulmonary bypass and without the use of a second side-biting clamp (single clamp technique, single TAC-ON); (7) on-pump technique with proximal anastomoses performed with the use of a side-biting clamp after releasing the aortic clamp (TAC-SBCL-ON).

*Model 2—Patients stratified by the number of touches on the aorta:* (1) No-touch; (2) one-touch (side-biting clamp or aortic cannula); (3) two touches (side-biting clamp plus aortic cannula); (4) three touches (total aortic clamp, aortic cannula, and aortic needle); (5) four touches (a full aortic clamp, side-biting clamp, aortic cannula, and aortic needle); (6) three or more touches without proximal anastomosis, with a single total aortic clamp, and with a full aortic clamp and side-biting clamp.

*Model 3—Patients stratified by the use of cardiopulmonary bypass (CPB):* (1) Off-pump; (2) on-pump, with or without the use of a total aortic clamp.

*Model 4—Patients stratified by the use of a total aortic clamp:* (1) No TAC; (2) TAC, with or without the use of a second side-biting clamp.

*Model 5—Patients stratified by the use of a side-biting clamp:* (1) No SBCL, without proximal anastomoses or without using the single clamp technique; (2) SBCL with or without TAC.

*Model 6—Patients stratified by the creation and number of proximal anastomoses:* (1) No proximal anastomoses; (2) proximal anastomosis; (3) one proximal anastomosis; (4) two proximal anastomoses; (5) three or more proximal anastomoses.

Conventional full median sternotomy CABG was performed in a standard fashion. When surgery was carried out on-pump, middle hypothermic (30–32°C), CPB was instituted by cannulating the ascending aorta and the right atrium. In some patients, a cross-clamp was released at the end of the distal anastomoses, and an SBCL was applied once or twice to make proximal anastomoses. For the patients in the single TAC-on group, the aortic cross-clamp remained in place for both distal and proximal coronary anastomoses. When the intervention was performed off-pump, stabilization of the target arteries was accomplished with the Medtronic Octopus System (Medtronic, Minneapolis, MN). Regarding the beating heart on-pump technique, surgery was performed on the beating heart specifically on normothermic (32°C) CPB established by standard ascending aorta cannulation and the insertion of a two-stage venous cannula with no additional venting of the left ventricle. In the non-touch technique, no proximal anastomosis was performed either because it was unnecessary or because of the use of one or two internal thoracic arteries (ITAs) and/or Y graft anastomosed to ITAs. Data obtained preoperatively, surgically, and postoperatively in the unweighted population are shown in the Supplementary Tables 1–3.

### Definition of Ischemic Stroke

The objective of the present study was to estimate how frequently postoperative ischemic stroke may occur within 30 days of the procedure. Following the American Heart Association/American Stroke Association, ischemic stroke was defined as an acute episode of neurological dysfunction caused by focal cerebral, spinal, or retinal infarction with a diagnosis based on symptoms persisting 24 or more hours or until death on pathological imaging, or other objective evidence of ischemic injury in a defined vascular distribution, and excluding other pathologies (e.g., hemorrhagic infarction) (13).

### Statistical Analysis

Propensity score (PS) inverse-probability of treatment weighting estimation for multiple treatments was used, and six independent models were created. The balance was tested either graphically or utilizing balance tables. Graphical estimation used standardized effect plots and quantile-quantile plots, which provide an immediate visual evaluation of balance quality. In each model, absolute standardized mean differences (ASMD) were calculated using a cutoff of <0.20 for bias statistics. The average treatment effect (ATE) was chosen as the causal effect estimand of stroke. It was defined as the ratio of the incidence of stroke in the entire population undergoing one treatment over the impact of stroke of the whole population under another treatment.

R software v. 3.6.1 and specific packages were used for analysis. More details can be found in the Supplementary Material.

## Results

### Balance

Balance plots and the explanation of how to interpret them are presented in the Supplementary Figures 4–9. Briefly, both the standardized effect plots and the quantile-quantile plots indicate an optimal balance across the models. Supplementary Table 10 summarizes the results of the balance tables. Using models, without weights, the ASMD was ≥0.20 in 30–51% of the cases. No imbalance was observed using models with weights, and groups were sufficiently similar to support causal estimation of the treatment estimand. The complete balance tables are shown in the Supplementary Tables 11–16.

### Treatment Estimand: Ischemic Stroke

*Model 1. Stratification by surgical technique*

**Figure 2:**
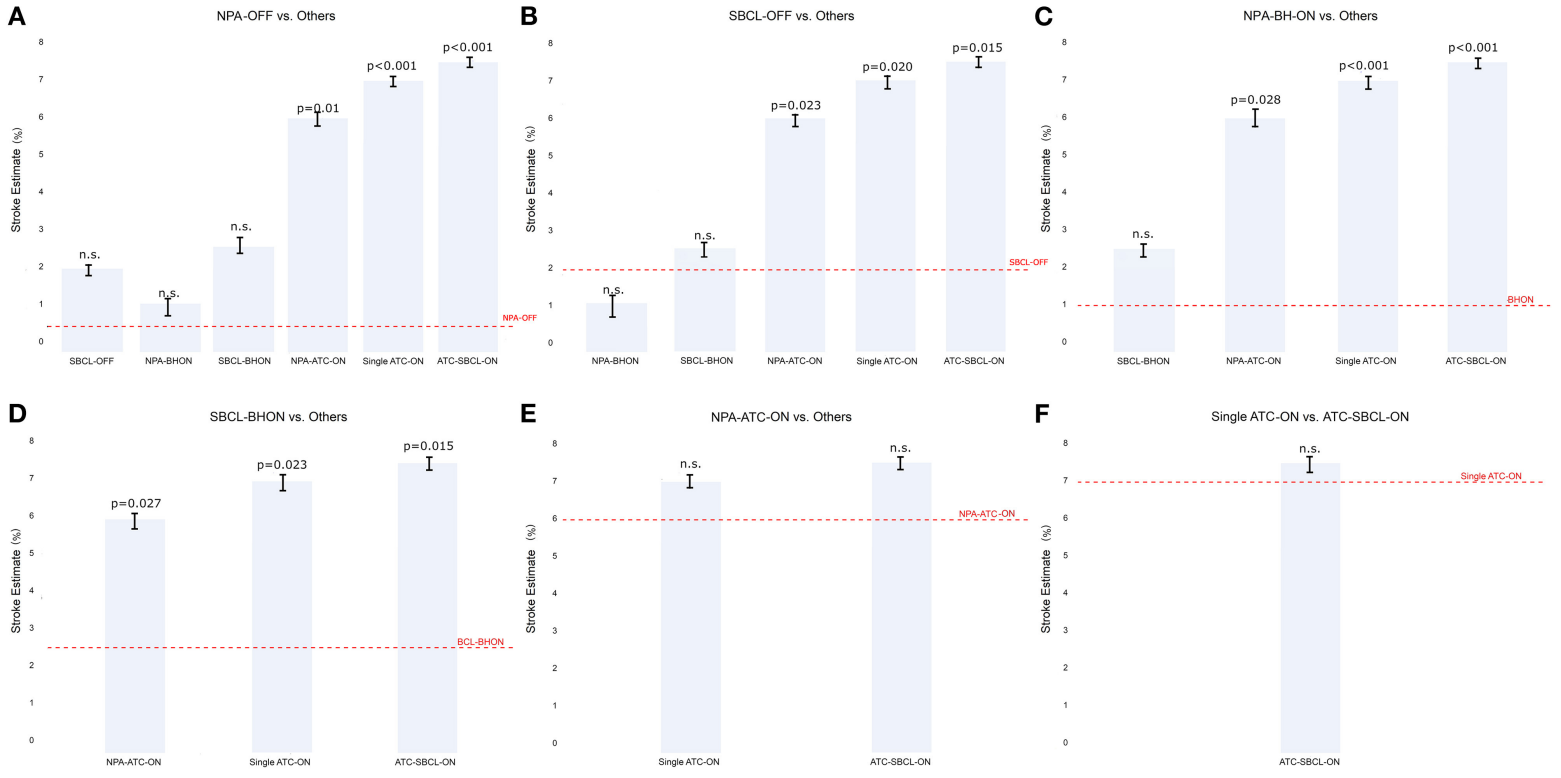
**(A–F)** The estimate of stroke in Model 1 (patients stratified by surgical technique). Any single technique is compared to the others.Abbreviations as in Figure 1.

Stroke estimate was significantly lower in NPA-off, SBCL-off, NPA-BH-on, and SBCL-BH-on than in all the other methods without significant differences between these procedures (Figures 2A–D). Also, ischemic stroke estimate was comparable between NPA-TAC-on, single TAC-on, and TAC-SBCL-on (Figures 2E,F).

*Model 2. Stratification by number of aortic touches*

**Figure 3:**
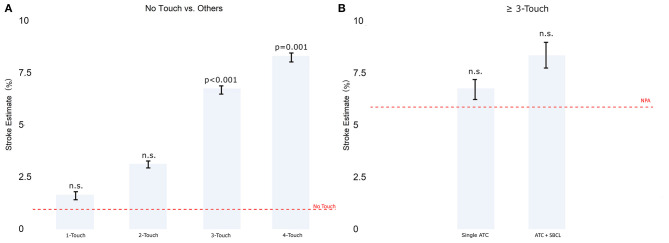
**(A)** The estimate of stroke in Model 2 (patients stratified by the number of touches on the aorta). **(B)** Comparison between single TAC and TAC+ SBCL in ≥3 touches. Abbreviations as in Figure 1.

There was no difference between the no-touch technique and procedures involving one or two aortic touches. In contrast, the ischemic stroke estimate was significantly higher in the case of three or four touches, with no significant difference between three and four touches (Figure 3A). When aortic touches were three or more, there was no significant difference between using a single clamp, a double clamp, or a no-proximal anastomosis technique (Figure 3B).

*Model 3 Stratification by cardiopulmonary bypass*

**Figure 4:**
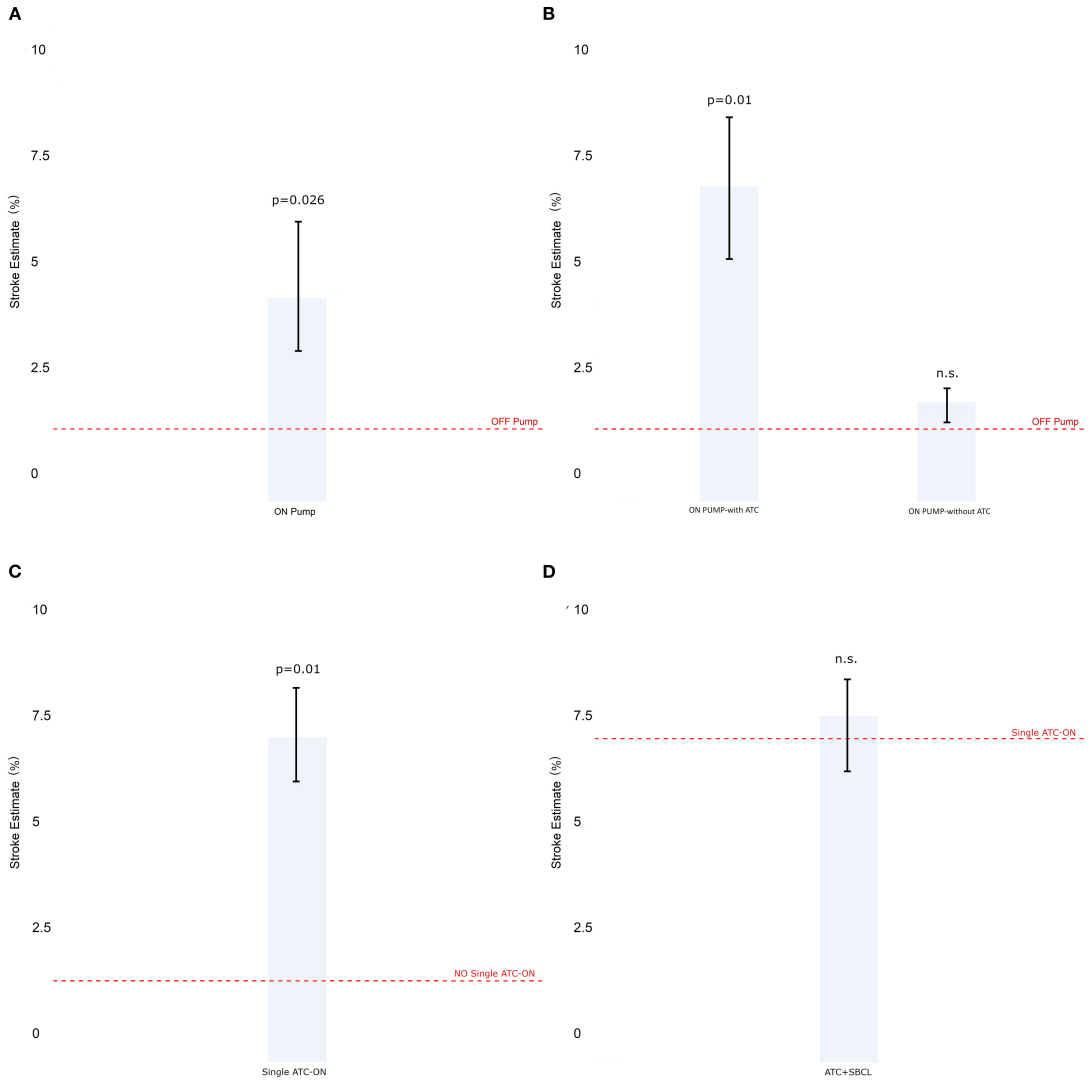
The estimate of stroke in Model 3 (patients stratified by cardiopulmonary bypass). **(A)** Off-pump vs. on-pump. **(B)** Off-pump vs. on-pump with or without a total aortic total clamp. **(C)** The estimate of stroke in Model 4 (patients stratified by total aortic clamp). **(D)** Single vs. double clamp (TAC+SBCL). Abbreviations as in Figure 1.

The incidence of stroke was significantly higher in CABG on-pump compared with the off-pump technique (Figure 4A). Nonetheless, in this group, the patients who underwent beating heart surgery with cardiopulmonary bypass without an aortic clamp and cardioplegic arrest, showed a similar incidence of stroke compared to OPCAB which was anyway significantly lower than that when on-pump with clamping was used (Figure 4B).

*Model 4. Stratification by total aortic clamping*

The use of full clamping significantly increased the estimate of stroke (Figure 4C). Besides, no significant difference was found between performing anastomoses with a double clamp or performing them under pulmonary bypass with the aorta clamped and without using a side-biting clamp (Figure 4D).

*Model 5. Stratification with a side-clamp for proximal anastomoses*

**Figure 5:**
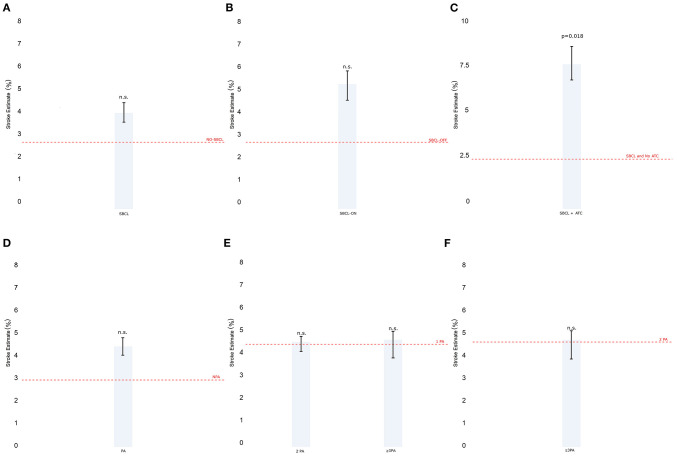
**(A)** The estimate of stroke in Model 5 (patients stratified by the side-biting clamp). **(B)** SBCL on-pump vs. off-pump. **(C)** SBCL with or without an aortic cross-clamp. **(D)** The estimate of stroke in Model 6 (patients stratified by creation and number of proximal anastomoses). **(E)** One proximal anastomosis vs. > 1 proximal anastomosis. **(F)** Two proximal anastomoses vs. ≥ 3 proximal anastomoses.

The use of a side-biting clamp for proximal anastomoses did not influence the estimate of ischemic stroke rate (Figure 5A). Furthermore, using a side-clamp on- and off-pump was comparable (Figure 5B). In contrast, the association of the side-biting clamp with a total clamp significantly increased the incidence of stroke (Figure 5C).

*Model 6. Stratification by number of proximal anastomoses*

The performance of one or more anastomoses slightly increased the ischemic stroke estimate without reaching statistical significance (Figure 5D). However, the difference between making one or more proximal anastomoses was not significant (Figures 5E,F).

Complete estimand tables and two-by-two comparisons are shown in the Supplementary Tables 17–28.

## Discussion

Several techniques have been proposed to minimize the risk of ischemic stroke (2–7). Nonetheless, despite the plethora of research published on the topic, it is not possible to draw any conclusions on the matter because of significant research limitations of published studies such as significant baseline imbalances (i.e., a higher number of double ITAs and arterial revascularization in the non-touch technique; preoperative differences between OPCAB and on-pump, etc.) (10–12) and incomplete testing of all the CABG techniques available and the combination thereof on the incidence of postoperative ischemic stroke. The creation of six independent multinomial PS-models achieving optimal balance enabled us to obtain unbiased estimates of the treatment effect with any combination of the techniques employed and, at the same time, to retain all the patients in the cohort (14).

The main findings of the paper can be summarized as follows:

1. The total aortic cross-clamp (TAC) turned out to be the main cause of postoperative stroke, whereas using a partial side-biting clamp did not affect its incidence. Model 1 shows that the estimate of stroke becomes significantly higher each time TAC is employed. Indeed, once the aortic clamp is added, a higher number of touches (Model 2) or proximal anastomoses (Model 6), as well as the use of the side-biting clamp (Model 6), does not add any significant risk. Barbut et al. (15) in 1994 showed that the majority of emboli detected by transcranial Doppler ultrasonography during CABG were associated with the release of both an aortic cross-clamp and a side-biting clamp. Still, the largest number of embolic signals were detected after the removal of TAC. Nonetheless, in the following years, the attention of researchers focused mainly on SBCL (2, 12, 13, 16), and, to the extent of our knowledge, this is the first study that specifically analyzed the influence of TAC on the incidence of post-CABG-ischemic stroke.
2. One of the main hypotheses rejected by our paper is that the off-pump technique may reduce the incidence of stroke. Different theories have been proposed to explain the potential beneficial effect of off-pump surgery (17), including reduced inflammatory response (18), lower coagulation impairment (19), reduction in atrial fibrillation occurrence (17), and reduced incidence of episodes of hypotension and cerebral hypoperfusion (20). Nonetheless, in a recent randomized multicenter trial, off-pump CABG did not result in lower stroke rates (21) confirming data from the main off-pump vs. on-pump trials (22–25). A recent paper (26) has associated the use of OPCAB and single clamp technique during on pump to significantly lower postoperative stroke rates. Nonetheless, this paper is a one-to-one comparison and does not fully consider the influence of other potential technical factors when associated with OPCAB.

Indeed, in our experience, although the estimate of stroke was higher on the whole for on-pump patients, a more detailed analysis showed that this difference disappears when the TAC is considered (Model 3). Moreover, Model 1 shows comparable shock estimates between the off-pump technique and surgery performed on the beating heart. This finding demonstrates that the use of CPB itself does not add any risk of stroke.

3. Neither the aortic clamping strategy for constructing proximal anastomoses in CABG nor the number of anastomoses affected the incidence of postoperative ischemic stroke.

More specifically, Model 5 failed to find any difference in the estimate of stroke employing a side-biting clamp, either off-pump or on the beating heart, on CPB. This finding is in accordance with Emmert et al., who reported no change in stroke rate when comparing on-pump CABG vs. applying partial aortic cross-clamping in OPCAB (12) and with Chu et al. who did not identify any significant differences in the incidence of postoperative stroke regardless of the clamping method used to perform a proximal anastomosis (27).

Moreover, Model 6 found no difference in stroke estimate whether proximal anastomoses were performed or not. This finding is in line with the lack of influence of SBCL on stroke. In addition, in Model 2, we found that one or two aortic touches did not influence the incidence of stroke. Nonetheless, three or four touches always included the use of the aortic cross-clamp. Hence the risk of stroke is independent of the number of touches and increases when TAC is used, independently of the technique employed.

4. Our findings do not support the hypothesis that the incidence of post-CABG embolic stroke decreases by eliminating the second, partially occluding aortic clamp. Model 4 indicated clearly that the estimate of stroke did not increase with the application of the conventional second clamp for performing proximal anastomosis on an arrested heart. This result conforms with large studies showing similar rates of stroke with single or double cross-clamp techniques (28–30).
5. Finally, our data do not confirm the hypothesis that cannulation of the aorta increases the risk of stroke. Model 1 indicated that, by adding aortic cannulation, with or without proximal anastomoses, the ischemic stroke estimate did not increase, and this was also confirmed by Model 3, showing comparable results between off-pump and on-pump without a cross-clamp. This finding is in agreement with the increasing evidence coming from other cardiac interventions that aortic cannulation involves low stroke risk (31)

## Clinical Repercussions

In the real clinical world, the non-touch technique is seldom applicable. Furthermore, the use of double ITAs, radial artery, and Y or T anastomosis is not always possible, and these procedures are not unanimously accepted by the cardiac surgery community (32). Thus, the daily-work life of a cardiac surgeon very often makes him/her deal with a certain degree of aortic touching with the consequent need to choose among different technical options. Our findings refute the choice of using the anaortic technique (33–35) “at any cost.” Indeed, from our experience, avoiding the aortic cross-clamp leaves a wide range of options, from performing proximal anastomoses using a side-biting clamp off-pump to working on the beating heart on normothermic CPB. Beating-heart-non-cardioplegic CABG should be preferred in case of risk of incomplete revascularization or of bypass construction on difficult-to-reach or remote areas of the heart to avoid hemodynamic deterioration and hypoperfusion, to unload the heart, and to preserve the native coronary blood flow (36, 37).

If a patient should require a CABG technique entailing the use of CPB, our findings suggest that there is no difference between performing proximal anastomoses with a side-biting clamp after releasing the total aortic cross-clamp or creating an anastomosis on full CPB with the aorta cross-clamped. Hence, our data recommend caution in choosing the single clamp technique and suggest carefully evaluating every individual patient for the potential advantages of the double-clamp technique, including better myocardial protection (38), compared to the disadvantages of the single-clamp method such as prolonging myocardial ischemic time and increasing the risk of cardiac and cerebral air embolism.

Finally, our data provide insufficient evidence to justify either the attempt to perform as many anastomoses as possible with a single partial clamp or to reduce the number of proximal anastomoses on the aorta.

## Limitations

The current research is retrospective and, therefore, susceptible to all the inherent weaknesses of such studies. Second, no systematic assessment of the ascending aorta with epi-aortic ultrasound and transesophageal echocardiography was carried out. Third, the number of patients with facilitating devices or anastomotic connectors could not be included because the group was too small. Fourth, ischemic stroke was analyzed only from a surgical point of view, without taking into account other factors (inflammation, coagulation, etc.). Fifth, despite our aims, it was not possible to distinguish damages from TAC from potential injuries caused by the aortic needle since these two tools were always used together. Sixth, the majority of head computed tomography scans were performed early after surgery, so they may not have revealed some strokes detected by MRI afterward. Seventh, due to the number of patients between groups, we cannot exclude a Type 2 error. This should be taken into account when reading our results.

## Conclusions

The aortic cross-clamp is the leading cause of post-CABG ischemic stroke. In contrast, additional aortic manipulation from a side-biting clamp, on-pump surgery, multiple aortic touches, the number of proximal anastomoses, and aortic cannulation do not increase the risk of stroke. Further research on this topic is warranted.

## Perspectives

**Competency in Medical Knowledge:** The total aortic cross-clamp is the leading cause of postoperative stroke. The off-pump technique does not reduce the incidence of stroke.

**Competency in Patient Care:** The use of the “no-touch” technique “at any cost” is not justified. Performing proximal anastomoses using a side-biting clamp off-pump or working on the beating heart on normothermic CPB guarantee comparable safety in terms of stroke compared to the fully “no-touch” technique.

**Translational Outlook 1:** The mechanism by which the total aortic cross-clamp causes more ischemic stroke requires further investigation. New clamp designs, as well as a cross-clamp imaging-guided treatment, need to be evaluated.

**Translational Outlook 2:** Additional research is needed in order to understand the interaction of technical factors and clinical risk factors (hypertension diabetes, dyslipidemia, etc.) to lower the incidence of stroke after CABG that is still high.

## Data Availability Statement

The original contributions presented in the study are included in the article/Supplementary Material, further inquiries can be directed to the corresponding author/s.

## Ethics Statement

Ethical approval was not provided for this study on human participants because the Italian National Laws regulating observational retrospective studies (law nr. 11960, released on 13/07/2004). Written informed consent for participation was not required for this study in accordance with the national legislation and the institutional requirements.

## Author Contributions

SG: conceptualization, methodology, validation, formal analysis, investigation, resources, writing—original draft, writing—review and editing, visualization, project administration, and supervision. CT: investigation, resources, data curation, writing—original draft, and validation. FM: software, validation, data curation, writing—original draft, validation, and visualization. SP: validation, investigation, resources, data curation, writing—review and editing, and visualization. OP: software, validation, investigation, resources, and data curation. EP: validation, formal analysis, investigation, resources, data curation, review and editing, and visualization. AD: resources, data curation, conceptualization, methodology, validation, and review and editing. GP and LM: resources, data curation, review and editing, and visualization. ML: conceptualization, validation, data curation, review and editing, visualization, project administration, and supervision. MB: conceptualization, methodology, validation, formal analysis, investigation, writing—review and editing, visualization, project administration, and supervision. All authors contributed to the article and approved the submitted version.

## Conflict of Interest

The authors declare that the research was conducted in the absence of any commercial or financial relationships that could be construed as a potential conflict of interest.

## Abbreviations

CABG: Coronary artery bypass grafting
NPA-OFF: Non-Touch Off-Pump
SBCL-OFF: Side-Clamp Off-Pump
NPA-BHON: No Proximal Anastomoses Beating Heart On-Pump
SBCL-BHON: Side-Clamp Beating Heart On-Pump
NPA-TAC-ON: No Proximal Anastomoses On-Pump
SINGLE TAC-ON: Single Total Clamp On-Pump
TAC-SBCL-ON: Side-Clamp On-Pump
CPB: Cardio-Pulmonary Bypass (minutes)
AF: Atrial Fibrillation.
