# Supplementary material for: Surgical Risk Factors for Ischemic Stroke Following Coronary Artery Bypass Grafting. A Multi-Factor Multimodel Analysis
Source: Front Cardiovasc Med. 2021 Jul 5;8:622480. doi: 10.3389/fcvm.2021.622480 (PMC8287035; doi:10.3389/fcvm.2021.622480)
Supplement: Supplementary file 1 [file Data_Sheet_1.pdf]

Supplemental Material

Table of Contents

		Page
**1. Data Unweighted population**		**4**
	Table S1. Patient Characteristics	5
	Table S2. Surgery	6
	Table.S3. Early outcomes	7
**2. Balance Graphs**		**8**
	**2.1** How to interpret the balance Graphs	9
	Figure S4. Model 1- Stratification by Surgical Technique	10
	Figure S5. Model 2- Stratification by Number of Aortic Touches	11
	Figure S6. Model 3- Stratification by Cardiopulmonary Bypass	12
	Figure S7. Model 4- Stratification by Aortic Total Clamp	13
	Figure S8. Model 5- Stratification by Side-clamp for Proximal Anastomoses	14
	Figure S9. Model 6- Stratification by Creation and Number of Proximal Anastomoses	15
**3. Balance Tables**		**16**
	Table S10. Summary of Balance Tables	17
	Table S11. Balance Table Model 1- Stratification by Surgical Technique	18
	Table S12. Balance Table Model 2- Stratification by Number of Aortic Touches	53
	Table S13. Balance Table Model 3- Stratification by Cardio Pulmonary Bypass	70
	Table S14. Balance Table Model 4- Stratification by Total Aortic Clamp	72
	Table S15. Balance Table Model 5- Stratification by Side-Biting Clamp	74
	Table S16. Balance Table Model 6- Stratification by Creation and Number of Proximal Anastomoses	76
**4. Estimate Tables**		**86**
	Table S17. Estimate Model 1- Stratification by surgical technique	87
	Table S18. Estimate Model 2- by Number of Aortic Touches	87
	Table S19. Estimate Model 3- by Cardiopulmonary Bypass	88
	Table S20. Estimate Model 4- by Aortic Total Clamp	88
	Table S21. Estimate Model 5- by Side Clamp	89
	Table S22. Estimate Model 6- Stroke by Proximal Anastomoses	89
**5. Stroke Estimate: p values two-by-two comparisons**		**90**
	Table S23. Two-by-two comparisons by Surgical Technique	91
	Table S24. Two-by-two comparisons by Number of Aortic Touches	91
	Table S25. Two-by-two comparisons by Stratification by Cardiopulmonary Bypass	92

Table S26. Two-by-two comparisons by Cardiopulmonary Bypass and by use of Total Aortic Clamp 92
Table S27. Two-by-two comparisons by Side-Biting Clamp 93
Table S28. Two-by-two comparisons by Creation of Proximal Anastomoses 93

6. Statistical Notes **94**

6.1 Advantages of the PS method over regression-based prediction 97
6.2 Pre-treatment variables 98
6.3 Variables included in the Propensity Score Models 98

7. STROBE **99**
8. Author Roles **103**
7.1 Database 100

9. Ethical Issues **104**

References **105**

1. Data Unweighted Population

Table S1. Patient Characteristics (Unweighted)

	Overall	NPA-OFF	SBCL-OFF	NPA-BHON	SBCL-BHON	NPA-TAC-ON	SINGLE TAC-ON	TAC-SBCL-ON	p
	16,255	1,062	1,794	840	1,561	937	3,970	6,091	
Age	68.00 [61.00, 74.00]	67.00 [60.00, 74.00]	68.00 [61.00, 74.75] a	70.00 [63.00, 76.00] b,g	68.00 [60.00, 74.00] l,h	67.00 [60.00, 74.00] m,i	67.00 [60.00, 73.00] n,q,j,u	68.00 [61.00, 75.00] o,t,f,r	<0.001
Gender (Male)	12993 (79.9)	847 (79.8)	1468 (81.8)	694 (82.6)	1280 (82.0)	692 (73.9) d,i,m,p	3139 (79.1) s	4873 (80.0) t	<0.001
Hypertension	9508 (58.5)	289 (27.2)	653 (36.4) a	267 (31.8)	1254 (80.3) c,h,l	463 (49.4) d,i,m,p	2727 (68.7) e,j,n,q,s	3855 (63.3) f,k,o,r,t,u	<0.001
Smoker	5959 (36.7)	147 (13.8)	220 (12.3)	96 (11.4) g	851 (54.5) c,h,l	281 (30.0) d,i,m,p	1934 (48.7) e,j,n,q,s	2430 (39.9) f,k,o,r,t,u	<0.001
Hypercholesterolemia	7189 (44.2)	151 (14.2)	275 (15.3)	109 (13.0)	1134 (72.6) c,h,l	350 (37.4) d,i,m,p	2136 (53.8) e,j,n,q,s	3034 (49.8) f,k,o,r,t,u	<0.001
Diabetes	4024 (24.8)	185 (17.4)	415 (23.1) a	172 (20.5) g	573 (36.7) c,m,l	206 (22.0) p	743 (18.7) j,q	1730 (28.4) f,k,o,r,t,u	<0.001
Insulin Dependent Diabetes	1517 (9.3)	57 (5.4)	48 (2.7) a	22 (2.6)	314 (20.1) c,h,l	67 (7.2) i,m,p	484 (12.2) e,j,n,q,s	525 (8.6) f,k,o,r,u	<0.001
COPD	1101 (6.8)	47 (4.4)	92 (5.1)	76 (9.0) b,g	34 (2.2) c,h,l	70 (7.5) p	353 (8.9) e,j,q	429 (7.0) f,r,u	<0.001
AKD	702 (4.3)	37 (3.5)	40 (2.2)	39 (4.6) g	26 (1.7)	44 (4.7) i,p	183 (4.6) j,q	333 (5.5) r	<0.001
Dialysis	146 (0.9)	6 (0.6)	4 (0.2)	9 (1.1)	18 (1.2) h	10 (1.1)	41 (1.0) j	58 (1.0) k	0.039
Stroke	463 (2.8)	15 (1.4)	18 (1.0)	15 (1.8)	8 (0.5)	28 (3.0) p	261 (6.6) e,j,n,q,s	118 (1.9) r,u	<0.001
TIA	494 (3.0)	14 (1.3)	19 (1.1)	15 (1.8)	25 (1.6)	20 (2.1)	157 (4.0) e,j,n,q	244 (4.0) f,k,o,r	<0.001
Carotid Stenosis > 50%	1401 (8.6)	56 (5.3)	108 (6.0)	105 (12.5) b,m	28 (1.8) c,h,l	51 (5.4) g,p	788 (19.8) e,j,n,q,s	265 (4.4) o,r,u	<0.001
PAD	2765 (17.0)	78 (7.3)	108 (6.0)	65 (7.7) d,i	369 (23.6) c,h,l	147 (15.7) m,p	912 (23.0) e,j,n,s	1086 (17.8) f,k,o,r,u	<0.001
Pulmonary Hypertension	797 (4.9)	17 (1.6)	46 (2.6)	33 (3.9) b	18 (1.2) l	46 (4.9) d,i,p	378 (9.5) e,j,n,q,s	259 (4.3) f,k,r,u	<0.001
IABP	257 (1.6)	19 (1.8)	32 (1.8)	10 (1.2)	54 (3.5) l	15 (1.6)	26 (0.7) e,j,q	101 (1.7) r,u	<0.001
NYHA ≥ 3	2481 (15.3)	112 (10.5)	156 (8.7)	45 (5.4) b	455 (29.1) c,h,l	154 (16.4) d,i,m,p	341 (8.6) n,q,s	1218 (20.0) f,k,o,r,u	<0.001
LVEF	55.00 [45.00, 60.00]	57.01 [50.00, 64.03]	56.00 [50.00, 60.00]	58.00 [50.00, 60.59]	55.00 [50.00, 60.00] l,c,h	55.00 [45.00, 60.00] m,d,p,i	50.00 [45.00, 60.00] n,s,e,q,j,u	55.00 [45.00, 60.00] o,t,f,k	<0.001
LMCA Stenosis	5316 (32.7)	224 (21.1)	548 (30.5) a	288 (34.3) b	540 (34.6) c	234 (25.0) m,p	1472 (37.1) e,j,s	2010 (33.0) f,t,u	<0.001
LAD Stenosis	15233 (93.7)	818 (77.0)	1776 (99.0) a	812 (96.7) b,g	1555 (99.6) c,l	865 (92.3) d,i,m,p	3663 (92.3) e,j,n,q	5744 (94.3) f,k,r,u	<0.001
2-Vessel Disease	5446 (33.5)	502 (47.3)	518 (28.9) a	451 (53.7) g	347 (22.2) c,h,l	357 (38.1) d,i,m,p	1168 (29.4) e,n,q,s	2103 (34.5) f,k,o,r,u	<0.001
≥ 3-Vessel Disease	9845 (60.6)	170 (16.0)	1286 (71.7) a	137 (16.3) g	1181 (75.7) c,l	153 (16.3) i,p	2928 (73.8) e,n,s	3990 (65.5) f,k,o,r,t,u	<0.001
Previuos PTCA	5096 (31.4)	118 (11.1)	526 (29.3) a	99 (11.8) g	253 (16.2) c,h	152 (16.2) d,i	671 (16.9) e,j,n	3277 (53.8) f,k,o,r,t,u	<0.001
Antiplatelet Drugs	5106 (31.4)	237 (22.3)	558 (31.1) a	235 (28.0)	492 (31.5) c	285 (30.4) d	1327 (33.4) e,n	1972 (32.4) f	<0.001
REDO	611 (3.8)	40 (3.8)	34 (1.9)	32 (3.8)	2 (0.1) c,h,l	49 (5.2) i,m,p	286 (7.2) e,j,q	168 (2.8) r,t,u	<0.001

Abbreviations: NPA-OFF: Non-Touch Off-Pump; SBCL-OFF: Side-Clamp Off-Pump; NPA-BHON: No Proximal Anastomoses Beating Heart On-Pump; SBCL-BHON: Side-Clamp Beating Heart On-Pump; NPA-TAC-ON: No Proximal Anastomoses On-Pump; SINGLE TAC-ON: Single Total Clamp On-Pump; TAC-SBCL-ON: Side-Clamp On-Pump; COPD: Chronic Obstructive Pulmonary Disease; AKD: Acute Kidney Disease; TIA: Transient Ischemic Attack; PAD: Peripheral Arterial Disease; IABP: Intra-Aortic Balloon Pump; NYHA: New York Heart Association Score; LVEF: Left Ventricular Ejection Fraction; LMCA: Left Main Coronary Artery; PTCA: Percutaneous Transluminal Coronary Angioplasty.

Statistical Significance: **a**. NPA-OFF vs SBCL-OFF; **b**. NPA-OFF vs NPA-BHON; **c**. NPA-OFF vs SBCL-BHON; **d**. NPA-OFF vs NPA-TAC-ON; **e**. NPA-OFF vs SINGLE TAC-ON; **f**. NPA-OFF vs TAC-SBCL-ON; **g**. SBCL-OFF vs NPA-BHON; **h**. SBCL-OFF vs SBCL-BHON; **i**. SBCL-OFF vs NPA-TAC-ON; **j**. SBCL-OFF vs SINGLE TAC-ON; **k**. SBCL-OFF vs SCL ON; **l**. NPA-BHON vs SBCL-BHON; **m**. NPA-BHON vs NPA-TAC-ON; **n**. NPA-BHON vs SINGLE TAC-ON; **o**. NPA-BHON vs TAC-SBCL-ON; **p**. SBCL-BHON vs NPA-TAC-ON; **q**. SBCL-BHON vs SINGLE TAC-ON; **r**. SBCL-BHON vs TAC-SBCL-ON; **s**. NPA-TAC-ON vs SINGLE TAC-ON; **t**. NPA-TAC-ON vs TAC-SBCL-ON; **u**. SINGLE TAC-ON vs TAC-SBCL-ON.

Table. S2. Surgery (Unweighted)

	Overall	NPA-OFF	SBCL-OFF	NPA-BHON	SBCL-BHON	NPA-TAC-ON	SINGLE TAC-ON	TAC-SBCL-ON	p
	**16,255**	**1,062**	**1,794**	**840**	**1,561**	**937**	**3,970**	**6,091**	
Surgery									<0.001
Urgency	8549 (52.6)	358 (33.7)	611 (34.1)	178 (21.2) b,g	1464 (93.8) c,h,l	393 (41.9) d,i,m,p	2863 (72.1) e,j,n,q,s	2682 (44.0) f,k,o,r,u	
Emergency	1406 (8.6)	36 (3.4)	78 (4.3)	39 (4.6)	97 (6.2) c	118 (12.6) d,i,m,p	635 (16.0) e,j,n,q	403 (6.6) f,k,t,u	
Elective	6300 (38.8)	668 (62.9)	1105 (61.6)	623 (74.2) b,g	-	426 (45.5) d,i,m,p	472 (11.9) e,j,n,q,s	3006 (49.4) f,k,o,r,u	
CPB time	68.00 [34.70, 90.00]	-	-	22.08 [9.18, 38.55]	91.00 [75.00, 113.00] l,c	50.00 [33.00, 73.00] m,p	58.00 [45.00, 74.00] n,s,q,j,u	90.00 [68.00, 93.00] o,t,r	<0.001
Clamping time	43.00 [20.95, 62.00]	-	-	-	-	32.34 [20.00, 49.00] m,d,p,i	33.00 [26.00, 45.00] n,e,q,j,u	60.00 [50.00, 70.00] o,t,f,r,k	<0.001
LITA	16,225 (99.8)	1,060 (99.8)	1,792 (99.8)	836 (99.5)	1,555 (99.6)	932 (99.5)	3,966 (99.9)	6,084 (99.9)	
BITA	3,986 (24.5)	900 (84.7)	384 (21.4)	747 (88.9)	464 (29.7)	844 (90.1)	366 (9.2)	281 (4.6)	
Radial Artery	1,073(6.6)	160 (15.1)	140 (7.8)	89 (10.6)	164 (10.5)	192 (20.5)	155 (3.9)	173 (2.8)	
Y Graft	755 (4.6)	167 (15.7)	86 (4.8)	95 (11.3)	58 (3.7)	208 (22.2)	77 (1.9)	64 (1.0)	
Distal Anastom/Patient	3.3±0.4	2.1±0.2	3.2±0.4	2.0±0.2	3.8±0.5	2.1±0.2	3.7±0.5	3.9±0.6	
Arterial Grafts/Patient	1.6±0.2	2.1±0.2	1.3±0.1	2.0±0.2	1.4±0.1	2.1±0.2	1.1±0.1	1.1±0.1	
Venous Grafts/Patient	2.1±0.2	-	1.9±0.2	-	2.4±0.2	-	2.6±0.3	2.8±0.4	
Prox Anastom/Side Clamp	1.3± 0.1	-	1.2±0.1	-	1.2±0.1	-	1.3±0.1	1.4±0.2	

Abbreviations: NPA-OFF: Non-Touch Off-Pump; SBCL-OFF: Side-Clamp Off-Pump; NPA-BHON: No Proximal Anastomoses Beating Heart On-Pump; SBCL-BHON: Side-Clamp Beating Heart On-Pump; NPA-TAC-ON: No Proximal Anastomoses On-Pump; SINGLE TAC-ON: Single Total Clamp On-Pump; TAC-SBCL-ON: Side-Clamp On-Pump; CPB: Cardio-Pulmonary Bypass (minutes); AF: Atrial Fibrillation.
Statistical Significance: **a**. NPA-OFF vs SBCL-OFF; **b**. NPA-OFF vs NPA-BHON; **c**. NPA-OFF vs SBCL-BHON; **d**. NPA-OFF vs NPA-TAC-ON; **e**. NPA-OFF vs SINGLE TAC-ON; **f**. NPA-OFF vs TAC-SBCL-ON; **g**. SBCL-OFF vs NPA-BHON; **h**. SBCL-OFF vs SBCL-BHON; **i**. SBCL-OFF vs NPA-TAC-ON; **j**. SBCL-OFF vs SINGLE TAC-ON; **k**. SBCL-OFF vs TAC-SBCL-ON; **l**. NPA-BHON vs SBCL-BHON; **m**. NPA-BHON vs NPA-TAC-ON; **n**. NPA-BHON vs SINGLE TAC-ON; **o**. NPA-BHON vs TAC-SBCL-ON; **p**. SBCL-BHON vs NPA-TAC-ON; **q**. SBCL-BHON vs SINGLE TAC-ON; **r**. SBCL-BHON vs TAC-SBCL-ON; **s**. NPA-TAC-ON vs SINGLE TAC-ON; **t**. NPA-TAC-ON vs TAC-SBCL-ON; **u**. SINGLE TAC-ON vs TAC-SBCL-ON.

Table S3. Early Outcomes (Unweighted)

	Overall<br>16,255	NPA-OFF<br>1,062	SBCL-OFF<br>1,794	NPA-BHON<br>840	SBCL-BHON<br>1,561	NPA-TAC-ON<br>937	SINGLE TAC-ON<br>3,970	TAC-SBCL-ON<br>6,091	p
Death	328 (2.0)	18 (1.7)	14 (0.8)	5 (0.6)	20 (1.3)	29 (3.1) i,m	110 (2.8) j,n,q	132 (2.2) k,o	<0.001
Cardiac Death	209 (1.3)	19 (1.8)	14 (0.8)	6 (0.7)	5 (0.3)c	19 (2.0) p	67 (1.7) q	79 (1.3) r	<0.001
Neurologic Death	70 (0.4)	-	6 (0.3)	-	2 (0.1)	17 (1.8) d,i,m,p	4 (0.1) s	41 (0.7) t,u	<0.001
Stroke	159 (1.0)	7 (0.7)	11 (0.6)	3 (0.4)	16 (1.0)	13 (1.4)	32 (0.8)	77 (1.3)	0.02
TIA	501 (3.1)	4 (0.4)	7 (0.4)	3 (0.4)	20 (1.3)	78 (8.3)d,i,m,p	139 (3.5)e,j,n,q,s	250 (4.1)f,k,o,r,t	<0.001
Bleeding	695 (4.3)	12 (1.1)	23 (1.3)	7 (0.8)	66 (4.2) c,h,l	34 (3.6) d,i,m	436 (11.0) e,j,n,q,s	117 (1.9) r,t,u	<0.001
Hypotension	955 (5.9)	13 (1.2)	37 (2.1)	10 (1.2)	133 (8.5) c,h,l	31 (3.3) d,p	580 (14.6) e,j,n,q,s	151 (2.5) r,u	<0.001
High Doses Vasoconstrictors	1571 (9.7)	18 (1.7)	109 (6.1) a	18 (2.1) g	240 (15.4) c,h,l	83 (8.9) d,m,p	777 (19.6) e,j,n,q,s	326 (5.4) f,o,r,t,u	<0.001
AF	2717 (16.7)	88 (8.3)	190 (10.6)	62 (7.4)	273 (17.5) c,h,l	182 (19.4) d,i,m	733 (18.5) e,j,n	1189 (19.5) f,k,o	<0.001
IABP	388 (2.4)	9 (0.8)	35 (2.0)	9 (1.1)	21 (1.3)	35 (3.7) d,m,p	93 (2.3) e	186 (3.1) f,o,r	<0.001
Impaired AV Conduction	467 (2.9)	14 (1.3)	12 (0.7)	1 (0.1)	64 (4.1) c,h,l	46 (4.9) d,i,m	142 (3.6) e,j,n	188 (3.1) f,k,o	<0.001
Transfusions	5626 (34.6)	146 (13.7)	264 (14.7)	129 (15.4)	213 (13.6)	375 (40.0) d,i,m,p	1431 (36.0) e,j,n,q	3068 (50.4) f,k,o,r,t,u	<0.001
AKD	1410 (8.7)	45 (4.2)	64 (3.6)	32 (3.8)	39 (2.5)	86 (9.2) d,i,m,p	648 (16.3) e,j,n,q,s	496 (8.1) f,k,o,u	<0.001
Anticoagulant Therapy	2282 (14.0)	38 (3.6)	69 (3.8)	49 (5.8)	41 (2.6) l	137 (14.6) d,i,m,p	476 (12.0) e,j,n,q	1472 (24.2) f,k,o,r,t,u	<0.001

Abbreviations: NPA-OFF: Non-Touch Off-Pump; SBCL-OFF: Side-Clamp Off-Pump; NPA-BHON: No Proximal Anastomoses Beating Heart On-Pump; SBCL-BHON: Side-Clamp Beating Heart On-Pump; NPA-TAC-ON: No Proximal Anastomoses On-Pump; SINGLE TAC-ON: Single Total Clamp On-Pump; TAC-SBCL-ON: Side-Clamp On-Pump; IABP: Intra-Aortic Balloon Pump; AV: Atrioventricular; AKD: Acute Kidney Disease.
Statistical Significance: **a**. NPA-OFF vs SBCL-OFF; **b**. NPA-OFF vs NPA-BHON; **c**. NPA-OFF vs SBCL-BHON; **d**. NPA-OFF vs NPA-TAC-ON; **e**. NPA-OFF vs SINGLE TAC-ON; **f**. NPA-OFF vs TAC-SBCL-ON; **g**. SBCL-OFF vs NPA-BHON; **h**. SBCL-OFF vs SBCL-BHON; **i**. SBCL-OFF vs NPA-TAC-ON; **j**. SBCL-OFF vs SINGLE TAC-ON; **k**. SBCL-OFF vs TAC-SBCL-ON; **l**. NPA-BHON vs SBCL-BHON; **m**. NPA-BHON vs NPA-TAC-ON; **n**. NPA-BHON vs SINGLE TAC-ON; **o**. NPA-BHON vs TAC-SBCL-ON; **p**. SBCL-BHON vs NPA-TAC-ON; **q**. SBCL-BHON vs SINGLE TAC-ON; **r**. SBCL-BHON vs TAC-SBCL-ON; **s**. NPA-TAC-ON vs SINGLE TAC-ON; **t**. NPA-TAC-ON vs TAC-SBCL-ON; **u**. SINGLE TAC-ON vs TAC-SBCL-ON.

2. Balance Graphs

2.1 How to Interpret the Balance Graphs

Standardized Effect Size plot. It assesses the balance of pretreatment variables before and after weighting. It shows the maximum pairwise absolute standardized mean differences (ASMDs). The ASMDs cutoff for defining unbalanced variables was 0.20. The light blue line represents pretreatment covariates for which the maximum pairwise ASMD reduced after weighting. The red lines mean the pretreatment covariates for which the maximum pairwise ASMD increased after weighting.

A good balance is obtained when, after weighting in the majority of variables the ASMDs are lower than 0.20 and there is a prevalence of light blue lines.

Quantile-quantile (Q-Q) plot. This plot also assesses the balance of pretreatment variables. In the plot, the Kolmogorov-Smirnov p-value is plotted against the rank of p-value for pretreatment variables. Along a 45-degree fitting line, open symbols represent weighted covariates and solid symbols represent unweighted covariates. A good balance is obtained when open symbols lie close, below or above, the 45-degree line.

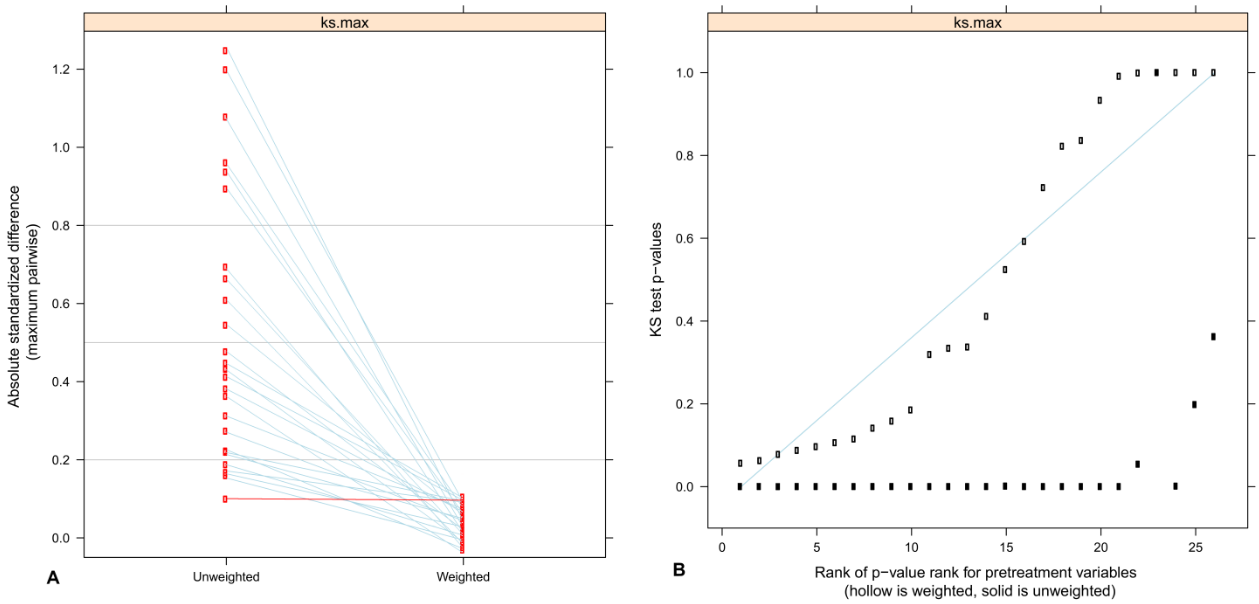

Figure S4 Model 1-stratification by surgical technique, 8000 iterations.

A. Standardized Effect Size plot. The weighted ASMD values were lower than 0.20 in all cases.

B. Quantile-quantile (Q-Q). Before weighting, most of the values were below the 45-degree line. A good balance was achieved after weighting, with p-values that lie close the 45-degree line.

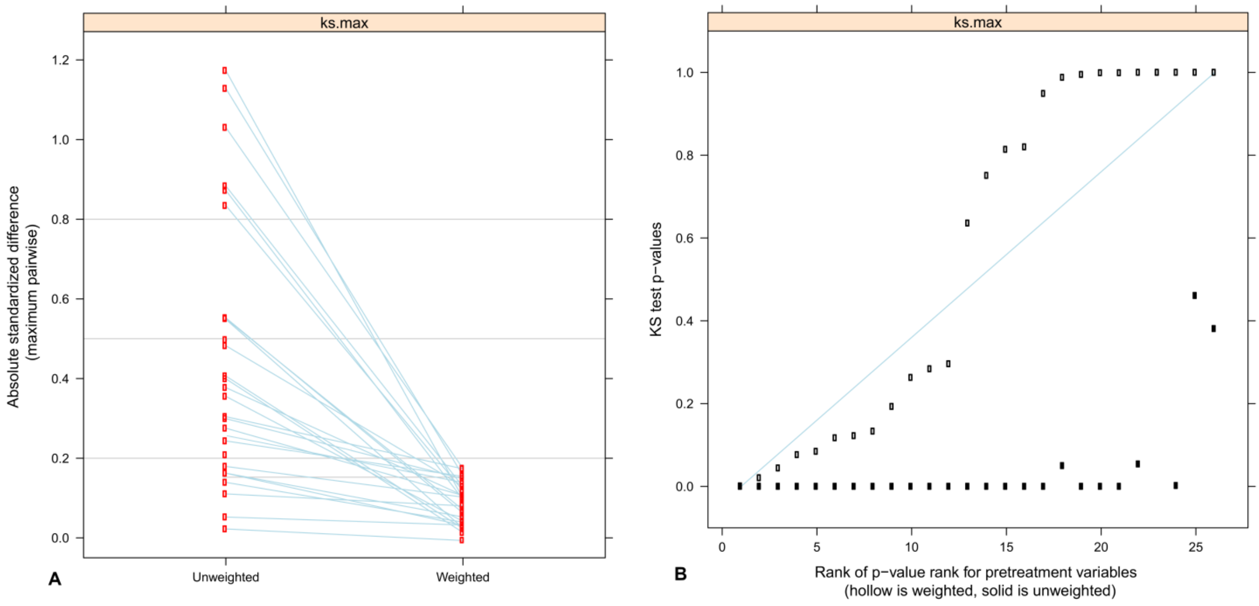

Figure S5. Model 2-stratification by number of aortic touches, 8000 iterations. A. Std.eff.sz: Standardized Effect Size plot. The weighted ASMD values were lower than 0.20 in all cases. B. Quantile-quantile (Q-Q). A good balance was achieved after weighting, with p-values with p-values that lie close the 45-degree line.

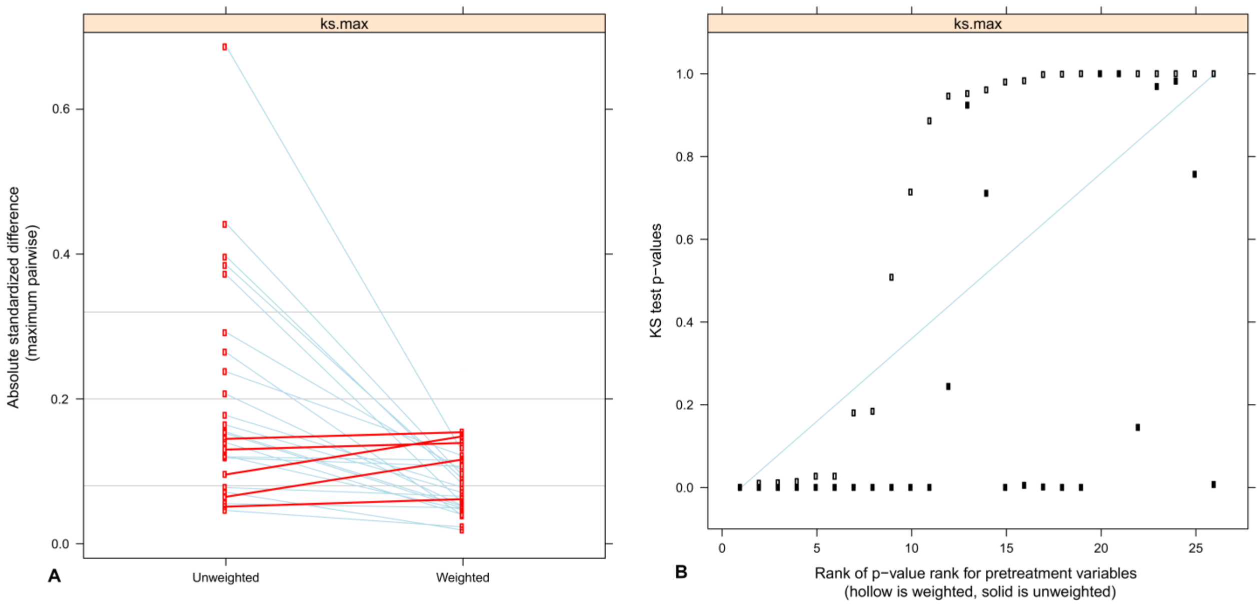

Figure S6. Model 3-stratification by cardiopulmonary bypass (CPB), 8000 iterations. A. Standardized Effect Size plot. The weighted ASMD values were lower than 0.20 in all cases. B. Quantile-quantile (Q-Q). A good balance was achieved after weighting, with most of p-values well above the 45-degree line.

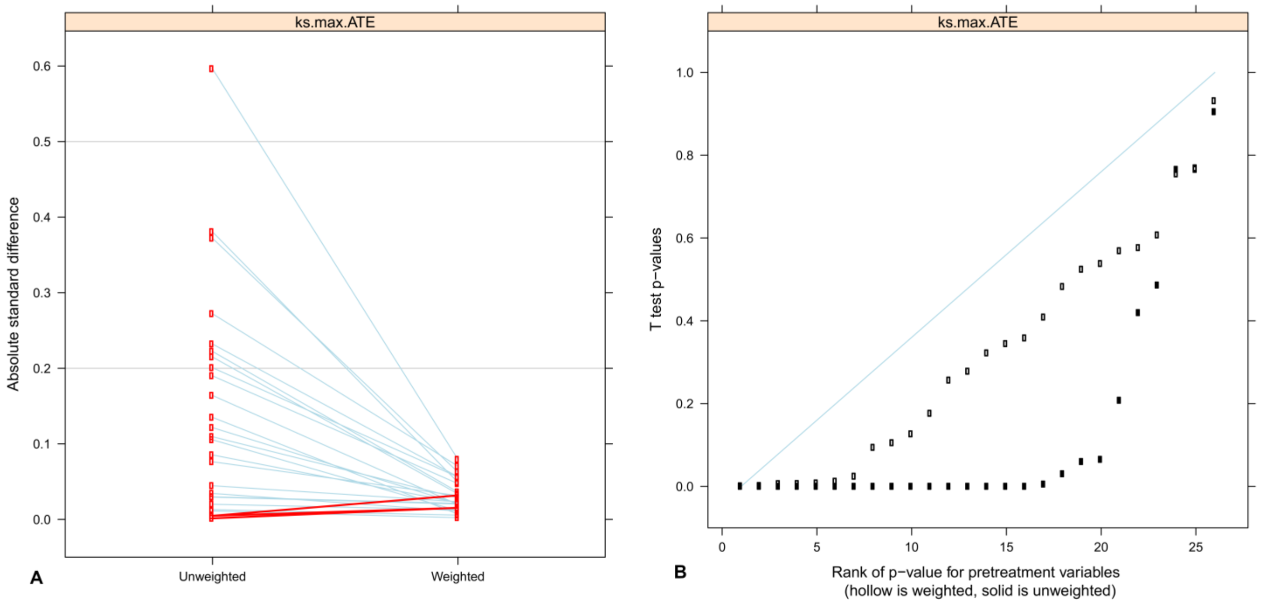

Figure S7. Model 4-stratification by total aortic clamp, 8000 iterations. A. Standardized Effect Size plot. The weighted ASMD values were lower than 0.20 in all cases. B. Quantile-quantile (Q-Q). A good balance was achieved after weighting, with most of p-values close to the 45-degree line.

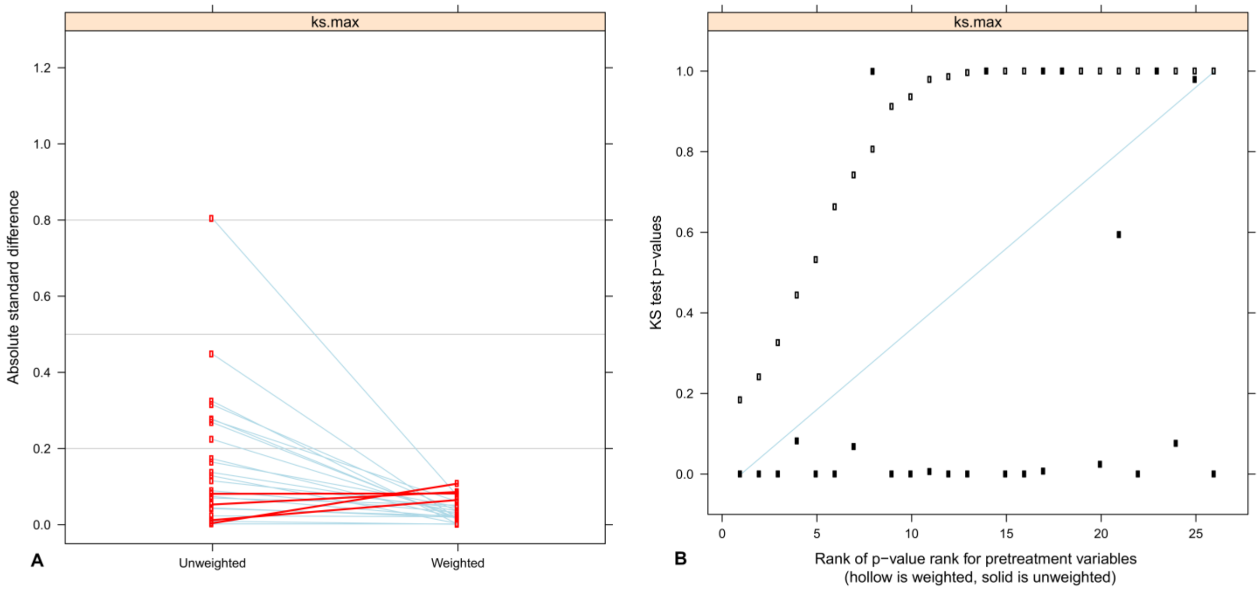

Figure S8. Model 5-stratification by the use of side-biting clamp for proximal anastomoses, 8000 iterations. A. Standardized Effect Size plot. The weighted ASMD values were lower than 0.20 in all cases. B. Quantile-quantile (Q-Q). A good balance was achieved after weighting, with most of p-values above the 45-degree line.

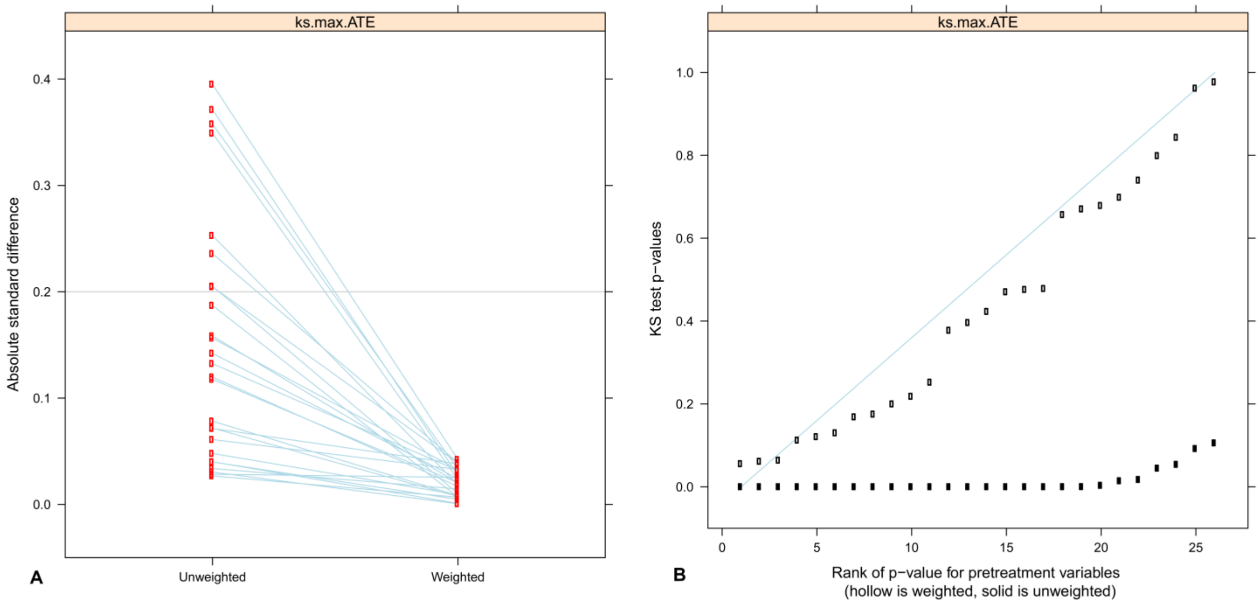

Figure S9. Model 6-stratification by Creation and Number of Proximal Anastomoses, 8000 iterations. A. Standardized Effect Size plot. The weighted ASMD values were lower than 0.20 in all cases. B. Quantile-quantile (Q-Q). A good balance was achieved after weighting, with most of p-values around the 45-degree line.

3. Balance Tables

Table S10. Summary of Balance Tables

	Before Weighting					After Weighting				
	**ASMD**	**SD**	**MIN**	**MAX**	**≥ 0.20**	**ASMD**	**SD**	**MIN**	**MAX**	**≥ 0.20**
**Model 1-**Stratification by surgical technique	0.23	0.25	0.01	1.25	214 (36.2)	0.07	0.04	0	0.18	0
**Model 2-S**tratification by number of aortic touches	0.24	0.25	0.01	1.22	110 (42.3)	0.06	0.05	0	0.19	0
**Model 3-S**tratification by CPB	0.16	0.17	0.01	0.81	8 (30.6)	0.03	0.02	0	0.09	0
**Model 4-**Stratification by total aortic clamping	0.15	0.11	0.03	0.4	8 (30.8)	0.02	0.01	0	0.04	0
**Model 5-S**tratification by side-clamping	0.14	0.14	0.01	0.6	8 (30.8)	0.03	0.02	0	0.08	0
**Model 6-S**tratification by proximal anastomoses	0.25	0.22	0.01	0.83	80 (51.3)	0.09	0.06	0	0.19	0
**Model 7-S**tratification by cannula	0.17	0.21	0.3	0.72	9 (34.6)	0.01	0.03	0.04	0.08	0

Abbreviations: ASMD: Absolute standardized mean difference; SD: Standard Deviation. An absolute SMD value ≥ 0.20 was the cutoff for imbalance. Cutoff SMD ≥ 0.20 is presented as number and (percentage) for each model. CPB: Cardiopulmonary bypass

Table S11. Balance Table Model 1. Stratification by Surgical Technique

tmt1	tmt2	var	mean1	mean2	pop.sd	std.eff.sz	p	ks	Stop Method
NPA-OFF	SBCL-OFF	Age	66.12	67.46	9.53	0.14	0	0.06	unw
NPA-OFF	SBCL-OFF	Gender(Male)	0.8	0.82	0.4	0.05	0.18	0.02	unw
NPA-OFF	SBCL-OFF	Hypertension	0.27	0.36	0.49	0.18	0	0.09	unw
NPA-OFF	SBCL-OFF	Smoke	0.14	0.13	0.48	0.02	0.39	0.01	unw
NPA-OFF	SBCL-OFF	Hypercholesterolemia	0.14	0.15	0.5	0.02	0.42	0.01	unw
NPA-OFF	SBCL-OFF	Diabetes	0.17	0.23	0.43	0.13	0	0.06	unw
NPA-OFF	SBCL-OFF	Insulin Dependent Diabetes	0.05	0.03	0.29	0.09	0	0.03	unw
NPA-OFF	SBCL-OFF	COPD	0.04	0.05	0.25	0.03	0.39	0.01	unw
NPA-OFF	SBCL-OFF	AKD	0.03	0.02	0.2	0.06	0.06	0.01	unw
NPA-OFF	SBCL-OFF	Dialysis	0.01	0	0.09	0.04	0.18	0	unw
NPA-OFF	SBCL-OFF	Stroke History	0.01	0.01	0.17	0.02	0.34	0	unw
NPA-OFF	SBCL-OFF	TIA History	0.01	0.01	0.17	0.01	0.64	0	unw
NPA-OFF	SBCL-OFF	Carotid Stenosis > 50%	0.05	0.06	0.27	0.03	0.4	0.01	unw
NPA-OFF	SBCL-OFF	PAD	0.07	0.06	0.38	0.04	0.18	0.01	unw
NPA-OFF	SBCL-OFF	Pulmonary Hypertension	0.01	0.03	0.21	0.08	0	0.02	unw
NPA-OFF	SBCL-OFF	IABP Preop	0.02	0.02	0.12	0.01	0.82	0	unw
NPA-OFF	SBCL-OFF	LVEF	55.54	55.09	11.36	0.04	0.29	0.06	unw
NPA-OFF	SBCL-OFF	LMCA Stenosis	0.21	0.31	0.47	0.2	0	0.1	unw
NPA-OFF	SBCL-OFF	Two Vessels Disease	0.47	0.29	0.47	0.38	0	0.18	unw
NPA-OFF	SBCL-OFF	Previuos PTCA	0.11	0.3	0.46	0.41	0	0.19	unw
NPA-OFF	SBCL-OFF	Antiplatelet Drugs	0.26	0.32	0.47	0.13	0	0.06	unw
NPA-OFF	SBCL-OFF	Surgery	0.03	0.04	0.28	0.03	0.19	0.01	unw
NPA-OFF	SBCL-OFF	REDO	0.04	0.02	0.2	0.1	0.01	0.02	unw
NPA-OFF	SBCL-OFF	NYHA ≥ 3	0.1	0.08	0.36	0.05	0.09	0.02	unw
NPA-OFF	SBCL-OFF	≥ 3-Vessel Disease	0.16	0.71	0.49	1.13	0	0.55	unw
NPA-OFF	SBCL-OFF	LAD Stenosis	0.77	0.99	0.24	0.91	0	0.22	unw
NPA-OFF	NPA-BHON	Age	66.12	68.8	9.53	0.28	0	0.12	unw
NPA-OFF	NPA-BHON	Gender(Male)	0.8	0.83	0.4	0.07	0.11	0.03	unw
NPA-OFF	NPA-BHON	Hypertension	0.27	0.32	0.49	0.09	0.03	0.05	unw
NPA-OFF	NPA-BHON	Smoke	0.14	0.12	0.48	0.05	0.13	0.02	unw

NPA-OFF	NPA-BHON	Hypercholesterolemia	0.14	0.13	0.5	0.02	0.58	0.01	unw
NPA-OFF	NPA-BHON	Diabetes	0.17	0.2	0.43	0.07	0.09	0.03	unw
NPA-OFF	NPA-BHON	Insulin Dependent Diabetes	0.05	0.03	0.29	0.1	0	0.03	unw
NPA-OFF	NPA-BHON	COPD	0.04	0.09	0.25	0.18	0	0.05	unw
NPA-OFF	NPA-BHON	AKD	0.03	0.05	0.2	0.06	0.21	0.01	unw
NPA-OFF	NPA-BHON	Dialysis	0.01	0.01	0.09	0.05	0.23	0.01	unw
NPA-OFF	NPA-BHON	Stroke History	0.01	0.02	0.17	0.02	0.52	0	unw
NPA-OFF	NPA-BHON	TIA History	0.01	0.02	0.17	0.03	0.42	0	unw
NPA-OFF	NPA-BHON	Carotid Stenosis > 50%	0.05	0.13	0.27	0.27	0	0.07	unw
NPA-OFF	NPA-BHON	PAD	0.07	0.08	0.38	0	0.9	0	unw
NPA-OFF	NPA-BHON	Pulmonary Hypertension	0.01	0.04	0.21	0.14	0	0.03	unw
NPA-OFF	NPA-BHON	IABP Preop	0.02	0.01	0.12	0.05	0.28	0.01	unw
NPA-OFF	NPA-BHON	LVEF	55.54	55.65	11.36	0.01	0.83	0.08	unw
NPA-OFF	NPA-BHON	LMCA Stenosis	0.21	0.34	0.47	0.28	0	0.13	unw
NPA-OFF	NPA-BHON	Two Vessels Disease	0.47	0.55	0.47	0.16	0	0.08	unw
NPA-OFF	NPA-BHON	Previuos PTCA	0.11	0.09	0.46	0.03	0.34	0.01	unw
NPA-OFF	NPA-BHON	Antiplatelet Drugs	0.26	0.29	0.47	0.07	0.1	0.03	unw
NPA-OFF	NPA-BHON	Surgery	0.03	0.05	0.28	0.04	0.21	0.01	unw
NPA-OFF	NPA-BHON	REDO	0.04	0.04	0.2	0.01	0.75	0	unw
NPA-OFF	NPA-BHON	NYHA ≥ 3	0.1	0.05	0.36	0.13	0	0.05	unw
NPA-OFF	NPA-BHON	≥ 3-Vessel Disease	0.16	0.15	0.49	0.01	0.75	0.01	unw
NPA-OFF	NPA-BHON	LAD Stenosis	0.77	0.97	0.24	0.81	0	0.2	unw
NPA-OFF	SBCL-BHON	Age	66.12	66.6	9.53	0.05	0.22	0.03	unw
NPA-OFF	SBCL-BHON	Gender(Male)	0.8	0.82	0.4	0.06	0.15	0.02	unw
NPA-OFF	SBCL-BHON	Hypertension	0.27	0.8	0.49	1.08	0	0.53	unw
NPA-OFF	SBCL-BHON	Smoker	0.14	0.54	0.48	0.84	0	0.4	unw
NPA-OFF	SBCL-BHON	Hypercholesterolemia	0.14	0.73	0.5	1.18	0	0.58	unw
NPA-OFF	SBCL-BHON	Diabetes	0.17	0.37	0.43	0.45	0	0.19	unw
NPA-OFF	SBCL-BHON	Insulin Dependent Diabetes	0.05	0.2	0.29	0.51	0	0.15	unw
NPA-OFF	SBCL-BHON	COPD	0.04	0.02	0.25	0.09	0	0.02	unw
NPA-OFF	SBCL-BHON	AKD	0.03	0.02	0.2	0.09	0.01	0.02	unw
NPA-OFF	SBCL-BHON	Dialysis	0.01	0.01	0.09	0.06	0.1	0.01	unw

NPA-OFF	SBCL-BHON	Stroke History	0.01	0.01	0.17	0.05	0.03	0.01	unw
NPA-OFF	SBCL-BHON	TIA History	0.01	0.02	0.17	0.02	0.55	0	unw
NPA-OFF	SBCL-BHON	Carotid Stenosis > 50%	0.05	0.02	0.27	0.13	0	0.03	unw
NPA-OFF	SBCL-BHON	PAD	0.07	0.24	0.38	0.43	0	0.16	unw
NPA-OFF	SBCL-BHON	Pulmonary Hypertension	0.01	0.01	0.21	0.02	0.28	0	unw
NPA-OFF	SBCL-BHON	IABP Preop	0.02	0.03	0.12	0.13	0.01	0.02	unw
NPA-OFF	SBCL-BHON	LVEF	55.54	52.22	11.36	0.29	0	0.23	unw
NPA-OFF	SBCL-BHON	LMCA Stenosis	0.21	0.35	0.47	0.29	0	0.14	unw
NPA-OFF	SBCL-BHON	Two Vessels Disease	0.47	0.22	0.47	0.53	0	0.25	unw
NPA-OFF	SBCL-BHON	Previuos PTCA	0.11	0.16	0.46	0.12	0	0.06	unw
NPA-OFF	SBCL-BHON	Antiplatelet Drugs	0.26	0.31	0.47	0.12	0	0.05	unw
NPA-OFF	SBCL-BHON	Surgery	0.03	0.06	0.28	0.1	0	0.03	unw
NPA-OFF	SBCL-BHON	REDO	0.04	0	0.2	0.19	0	0.04	unw
NPA-OFF	SBCL-BHON	NYHA ≥ 3	0.1	0.29	0.36	0.53	0	0.19	unw
NPA-OFF	SBCL-BHON	≥ 3-Vessel Disease	0.16	0.77	0.49	1.24	0	0.61	unw
NPA-OFF	SBCL-BHON	LAD Stenosis	0.77	1	0.24	0.94	0	0.23	unw
NPA-OFF	NPA-TAC-ON	Age	66.12	66.15	9.53	0	0.94	0.04	unw
NPA-OFF	NPA-TAC-ON	Gender(Male)	0.8	0.74	0.4	0.15	0	0.06	unw
NPA-OFF	NPA-TAC-ON	Hypertension	0.27	0.49	0.49	0.44	0	0.22	unw
NPA-OFF	NPA-TAC-ON	Smoker	0.14	0.29	0.48	0.32	0	0.15	unw
NPA-OFF	NPA-TAC-ON	Hypercholesterolemia	0.14	0.37	0.5	0.46	0	0.23	unw
NPA-OFF	NPA-TAC-ON	Diabetes	0.17	0.22	0.43	0.11	0.01	0.05	unw
NPA-OFF	NPA-TAC-ON	Insulin Dependent Diabetes	0.05	0.08	0.29	0.08	0.03	0.02	unw
NPA-OFF	NPA-TAC-ON	COPD	0.04	0.07	0.25	0.12	0	0.03	unw
NPA-OFF	NPA-TAC-ON	AKD	0.03	0.05	0.2	0.06	0.17	0.01	unw
NPA-OFF	NPA-TAC-ON	Dialysis	0.01	0.01	0.09	0.05	0.22	0.01	unw
NPA-OFF	NPA-TAC-ON	Stroke History	0.01	0.03	0.17	0.09	0.02	0.02	unw
NPA-OFF	NPA-TAC-ON	TIA History	0.01	0.02	0.17	0.05	0.17	0.01	unw
NPA-OFF	NPA-TAC-ON	Carotid Stenosis > 50%	0.05	0.05	0.27	0	0.97	0	unw
NPA-OFF	NPA-TAC-ON	PAD	0.07	0.15	0.38	0.21	0	0.08	unw
NPA-OFF	NPA-TAC-ON	Pulmonary Hypertension	0.01	0.04	0.21	0.17	0	0.04	unw
NPA-OFF	NPA-TAC-ON	IABP Preop	0.02	0.01	0.12	0.03	0.47	0	unw

NPA-OFF	NPA-TAC-ON	LVEF	55.54	52.96	11.36	0.23	0	0.11	unw
NPA-OFF	NPA-TAC-ON	LMCA Stenosis	0.21	0.25	0.47	0.08	0.05	0.04	unw
NPA-OFF	NPA-TAC-ON	Two Vessels Disease	0.47	0.39	0.47	0.19	0	0.09	unw
NPA-OFF	NPA-TAC-ON	Previuos PTCA	0.11	0.16	0.46	0.12	0	0.05	unw
NPA-OFF	NPA-TAC-ON	Antiplatelet Drugs	0.26	0.31	0.47	0.11	0.01	0.05	unw
NPA-OFF	NPA-TAC-ON	Surgery	0.03	0.13	0.28	0.33	0	0.09	unw
NPA-OFF	NPA-TAC-ON	REDO	0.04	0.05	0.2	0.05	0.3	0.01	unw
NPA-OFF	NPA-TAC-ON	NYHA ≥ 3	0.1	0.16	0.36	0.17	0	0.06	unw
NPA-OFF	NPA-TAC-ON	≥ 3-Vessel Disease	0.16	0.17	0.49	0.02	0.65	0.01	unw
NPA-OFF	NPA-TAC-ON	LAD Stenosis	0.77	0.92	0.24	0.63	0	0.15	unw
NPA-OFF	SINGLE TAC-ON	Age	66.12	65.83	9.53	0.03	0.38	0.04	unw
NPA-OFF	SINGLE TAC-ON	Gender(Male)	0.8	0.79	0.4	0.02	0.62	0.01	unw
NPA-OFF	SINGLE TAC-ON	Hypertension	0.27	0.66	0.49	0.78	0	0.39	unw
NPA-OFF	SINGLE TAC-ON	Smoker	0.14	0.44	0.48	0.63	0	0.3	unw
NPA-OFF	SINGLE TAC-ON	Hypercholesterolemia	0.14	0.5	0.5	0.72	0	0.36	unw
NPA-OFF	SINGLE TAC-ON	Diabetes	0.17	0.19	0.43	0.03	0.33	0.01	unw
NPA-OFF	SINGLE TAC-ON	Insulin Dependent Diabetes	0.05	0.11	0.29	0.2	0	0.06	unw
NPA-OFF	SINGLE TAC-ON	COPD	0.04	0.09	0.25	0.18	0	0.04	unw
NPA-OFF	SINGLE TAC-ON	AKD	0.03	0.05	0.2	0.06	0.09	0.01	unw
NPA-OFF	SINGLE TAC-ON	Dialysis	0.01	0.01	0.09	0.05	0.1	0	unw
NPA-OFF	SINGLE TAC-ON	Stroke History	0.01	0.07	0.17	0.31	0	0.05	unw
NPA-OFF	SINGLE TAC-ON	TIA History	0.01	0.04	0.17	0.15	0	0.03	unw
NPA-OFF	SINGLE TAC-ON	Carotid Stenosis > 50%	0.05	0.16	0.27	0.41	0	0.11	unw
NPA-OFF	SINGLE TAC-ON	PAD	0.07	0.24	0.38	0.44	0	0.17	unw
NPA-OFF	SINGLE TAC-ON	Pulmonary Hypertension	0.01	0.1	0.21	0.41	0	0.09	unw
NPA-OFF	SINGLE TAC-ON	IABP Preop	0.02	0.01	0.12	0.09	0.01	0.01	unw
NPA-OFF	SINGLE TAC-ON	LVEF	55.54	50.73	11.36	0.42	0	0.24	unw
NPA-OFF	SINGLE TAC-ON	LMCA Stenosis	0.21	0.39	0.47	0.38	0	0.18	unw
NPA-OFF	SINGLE TAC-ON	Two Vessels Disease	0.47	0.32	0.47	0.32	0	0.15	unw
NPA-OFF	SINGLE TAC-ON	Previuos PTCA	0.11	0.17	0.46	0.13	0	0.06	unw
NPA-OFF	SINGLE TAC-ON	Antiplatelet Drugs	0.26	0.33	0.47	0.16	0	0.07	unw
NPA-OFF	SINGLE TAC-ON	Surgery	0.03	0.15	0.28	0.43	0	0.12	unw

NPA-OFF	SINGLE TAC-ON	REDO	0.04	0.08	0.2	0.22	0	0.04	unw
NPA-OFF	SINGLE TAC-ON	NYHA ≥ 3	0.1	0.08	0.36	0.04	0.12	0.02	unw
NPA-OFF	SINGLE TAC-ON	≥ 3-Vessel Disease	0.16	0.72	0.49	1.15	0	0.56	unw
NPA-OFF	SINGLE TAC-ON	LAD Stenosis	0.77	0.93	0.24	0.65	0	0.16	unw
NPA-OFF	TAC-SBCL-ON	Age	66.12	67.47	9.53	0.14	0	0.07	unw
NPA-OFF	TAC-SBCL-ON	Gender(Male)	0.8	0.8	0.4	0.01	0.85	0	unw
NPA-OFF	TAC-SBCL-ON	Hypertension	0.27	0.63	0.49	0.73	0	0.36	unw
NPA-OFF	TAC-SBCL-ON	Smoker	0.14	0.4	0.48	0.54	0	0.26	unw
NPA-OFF	TAC-SBCL-ON	Hypercholesterolemia	0.14	0.5	0.5	0.72	0	0.36	unw
NPA-OFF	TAC-SBCL-ON	Diabetes	0.17	0.28	0.43	0.25	0	0.11	unw
NPA-OFF	TAC-SBCL-ON	Insulin Dependent Diabetes	0.05	0.09	0.29	0.11	0	0.03	unw
NPA-OFF	TAC-SBCL-ON	COPD	0.04	0.07	0.25	0.1	0	0.03	unw
NPA-OFF	TAC-SBCL-ON	AKD	0.03	0.05	0.2	0.1	0	0.02	unw
NPA-OFF	TAC-SBCL-ON	Dialysis	0.01	0.01	0.09	0.04	0.14	0	unw
NPA-OFF	TAC-SBCL-ON	Stroke History	0.01	0.02	0.17	0.03	0.18	0.01	unw
NPA-OFF	TAC-SBCL-ON	TIA History	0.01	0.04	0.17	0.16	0	0.03	unw
NPA-OFF	TAC-SBCL-ON	Carotid Stenosis > 50%	0.05	0.04	0.27	0.03	0.21	0.01	unw
NPA-OFF	TAC-SBCL-ON	PAD	0.07	0.18	0.38	0.28	0	0.1	unw
NPA-OFF	TAC-SBCL-ON	Pulmonary Hypertension	0.01	0.04	0.21	0.14	0	0.03	unw
NPA-OFF	TAC-SBCL-ON	IABP Preop	0.02	0.02	0.12	0.01	0.77	0	unw
NPA-OFF	TAC-SBCL-ON	LVEF	55.54	52.04	11.36	0.31	0	0.14	unw
NPA-OFF	TAC-SBCL-ON	LMCA Stenosis	0.21	0.33	0.47	0.25	0	0.12	unw
NPA-OFF	TAC-SBCL-ON	Two Vessels Disease	0.47	0.34	0.47	0.27	0	0.13	unw
NPA-OFF	TAC-SBCL-ON	Previuos PTCA	0.11	0.54	0.46	0.93	0	0.43	unw
NPA-OFF	TAC-SBCL-ON	Antiplatelet Drugs	0.26	0.33	0.47	0.15	0	0.07	unw
NPA-OFF	TAC-SBCL-ON	Surgery	0.03	0.07	0.28	0.12	0	0.03	unw
NPA-OFF	TAC-SBCL-ON	REDO	0.04	0.03	0.2	0.05	0.1	0.01	unw
NPA-OFF	TAC-SBCL-ON	NYHA ≥ 3	0.1	0.2	0.36	0.28	0	0.1	unw
NPA-OFF	TAC-SBCL-ON	≥ 3-Vessel Disease	0.16	0.65	0.49	1.01	0	0.49	unw
NPA-OFF	TAC-SBCL-ON	LAD Stenosis	0.77	0.94	0.24	0.72	0	0.17	unw
SBCL-OFF	NPA-BHON	Age	67.46	68.8	9.53	0.14	0	0.07	unw
SBCL-OFF	NPA-BHON	Gender(Male)	0.82	0.83	0.4	0.02	0.62	0.01	unw

SBCL-OFF	NPA-BHON	Hypertension	0.36	0.32	0.49	0.09	0.02	0.05	unw
SBCL-OFF	NPA-BHON	Smoker	0.13	0.12	0.48	0.02	0.39	0.01	unw
SBCL-OFF	NPA-BHON	Hypercholesterolemia	0.15	0.13	0.5	0.04	0.17	0.02	unw
SBCL-OFF	NPA-BHON	Diabetes	0.23	0.2	0.43	0.06	0.12	0.03	unw
SBCL-OFF	NPA-BHON	Insulin Dependent Diabetes	0.03	0.03	0.29	0	0.93	0	unw
SBCL-OFF	NPA-BHON	COPD	0.05	0.09	0.25	0.16	0	0.04	unw
SBCL-OFF	NPA-BHON	AKD	0.02	0.05	0.2	0.12	0	0.02	unw
SBCL-OFF	NPA-BHON	Dialysis	0	0.01	0.09	0.09	0.02	0.01	unw
SBCL-OFF	NPA-BHON	Stroke History	0.01	0.02	0.17	0.05	0.13	0.01	unw
SBCL-OFF	NPA-BHON	TIA History	0.01	0.02	0.17	0.04	0.2	0.01	unw
SBCL-OFF	NPA-BHON	Carotid Stenosis > 50%	0.06	0.13	0.27	0.25	0	0.07	unw
SBCL-OFF	NPA-BHON	PAD	0.06	0.08	0.38	0.04	0.17	0.01	unw
SBCL-OFF	NPA-BHON	Pulmonary Hypertension	0.03	0.04	0.21	0.06	0.1	0.01	unw
SBCL-OFF	NPA-BHON	IABP Preop	0.02	0.01	0.12	0.04	0.32	0	unw
SBCL-OFF	NPA-BHON	LVEF	55.09	55.65	11.36	0.05	0.18	0.06	unw
SBCL-OFF	NPA-BHON	LMCA Stenosis	0.31	0.34	0.47	0.08	0.07	0.04	unw
SBCL-OFF	NPA-BHON	Two Vessels Disease	0.29	0.55	0.47	0.54	0	0.26	unw
SBCL-OFF	NPA-BHON	Previuos PTCA	0.3	0.09	0.46	0.44	0	0.2	unw
SBCL-OFF	NPA-BHON	Antiplatelet Drugs	0.32	0.29	0.47	0.05	0.2	0.02	unw
SBCL-OFF	NPA-BHON	Surgery	0.04	0.05	0.28	0.01	0.84	0	unw
SBCL-OFF	NPA-BHON	REDO	0.02	0.04	0.2	0.11	0	0.02	unw
SBCL-OFF	NPA-BHON	NYHA ≥ 3	0.08	0.05	0.36	0.08	0.01	0.03	unw
SBCL-OFF	NPA-BHON	≥ 3-Vessel Disease	0.71	0.15	0.49	1.14	0	0.56	unw
SBCL-OFF	NPA-BHON	LAD Stenosis	0.99	0.97	0.24	0.09	0	0.02	unw
SBCL-OFF	SBCL-BHON	Age	67.46	66.6	9.53	0.09	0.01	0.03	unw
SBCL-OFF	SBCL-BHON	Gender(Male)	0.82	0.82	0.4	0	0.9	0	unw
SBCL-OFF	SBCL-BHON	Hypertension	0.36	0.8	0.49	0.89	0	0.44	unw
SBCL-OFF	SBCL-BHON	Smoker	0.13	0.54	0.48	0.87	0	0.42	unw
SBCL-OFF	SBCL-BHON	Hypercholesterolemia	0.15	0.73	0.5	1.16	0	0.57	unw
SBCL-OFF	SBCL-BHON	Diabetes	0.23	0.37	0.43	0.31	0	0.14	unw
SBCL-OFF	SBCL-BHON	Insulin Dependent Diabetes	0.03	0.2	0.29	0.61	0	0.17	unw
SBCL-OFF	SBCL-BHON	COPD	0.05	0.02	0.25	0.12	0	0.03	unw

SBCL-OFF	SBCL-BHON	AKD	0.02	0.02	0.2	0.03	0.24	0.01	unw
SBCL-OFF	SBCL-BHON	Dialysis	0	0.01	0.09	0.1	0	0.01	unw
SBCL-OFF	SBCL-BHON	Stroke History	0.01	0.01	0.17	0.03	0.1	0	unw
SBCL-OFF	SBCL-BHON	TIA History	0.01	0.02	0.17	0.03	0.23	0	unw
SBCL-OFF	SBCL-BHON	Carotid Stenosis > 50%	0.06	0.02	0.27	0.16	0	0.04	unw
SBCL-OFF	SBCL-BHON	PAD	0.06	0.24	0.38	0.47	0	0.18	unw
SBCL-OFF	SBCL-BHON	Pulmonary Hypertension	0.03	0.01	0.21	0.06	0.01	0.01	unw
SBCL-OFF	SBCL-BHON	IABP Preop	0.02	0.03	0.12	0.14	0	0.02	unw
SBCL-OFF	SBCL-BHON	LVEF	55.09	52.22	11.36	0.25	0	0.2	unw
SBCL-OFF	SBCL-BHON	LMCA Stenosis	0.31	0.35	0.47	0.08	0.01	0.04	unw
SBCL-OFF	SBCL-BHON	Two Vessels Disease	0.29	0.22	0.47	0.15	0	0.07	unw
SBCL-OFF	SBCL-BHON	Previuos PTCA	0.3	0.16	0.46	0.29	0	0.13	unw
SBCL-OFF	SBCL-BHON	Antiplatelet Drugs	0.32	0.31	0.47	0.01	0.79	0	unw
SBCL-OFF	SBCL-BHON	Surgery	0.04	0.06	0.28	0.07	0.02	0.02	unw
SBCL-OFF	SBCL-BHON	REDO	0.02	0	0.2	0.09	0	0.02	unw
SBCL-OFF	SBCL-BHON	NYHA ≥ 3	0.08	0.29	0.36	0.59	0	0.21	unw
SBCL-OFF	SBCL-BHON	≥ 3-Vessel Disease	0.71	0.77	0.49	0.11	0	0.05	unw
SBCL-OFF	SBCL-BHON	LAD Stenosis	0.99	1	0.24	0.03	0.02	0.01	unw
SBCL-OFF	NPA-TAC-ON	Age	67.46	66.15	9.53	0.14	0	0.06	unw
SBCL-OFF	NPA-TAC-ON	Gender(Male)	0.82	0.74	0.4	0.2	0	0.08	unw
SBCL-OFF	NPA-TAC-ON	Hypertension	0.36	0.49	0.49	0.26	0	0.13	unw
SBCL-OFF	NPA-TAC-ON	Smoker	0.13	0.29	0.48	0.34	0	0.16	unw
SBCL-OFF	NPA-TAC-ON	Hypercholesterolemia	0.15	0.37	0.5	0.43	0	0.21	unw
SBCL-OFF	NPA-TAC-ON	Diabetes	0.23	0.22	0.43	0.03	0.49	0.01	unw
SBCL-OFF	NPA-TAC-ON	Insulin Dependent Diabetes	0.03	0.08	0.29	0.18	0	0.05	unw
SBCL-OFF	NPA-TAC-ON	COPD	0.05	0.07	0.25	0.09	0.02	0.02	unw
SBCL-OFF	NPA-TAC-ON	AKD	0.02	0.05	0.2	0.12	0	0.02	unw
SBCL-OFF	NPA-TAC-ON	Dialysis	0	0.01	0.09	0.09	0.02	0.01	unw
SBCL-OFF	NPA-TAC-ON	Stroke History	0.01	0.03	0.17	0.12	0	0.02	unw
SBCL-OFF	NPA-TAC-ON	TIA History	0.01	0.02	0.17	0.06	0.06	0.01	unw
SBCL-OFF	NPA-TAC-ON	Carotid Stenosis > 50%	0.06	0.05	0.27	0.03	0.39	0.01	unw
SBCL-OFF	NPA-TAC-ON	PAD	0.06	0.15	0.38	0.24	0	0.09	unw

SBCL-OFF	NPA-TAC-ON	Pulmonary Hypertension	0.03	0.04	0.21	0.09	0.02	0.02	unw
SBCL-OFF	NPA-TAC-ON	IABP Preop	0.02	0.01	0.12	0.02	0.56	0	unw
SBCL-OFF	NPA-TAC-ON	LVEF	55.09	52.96	11.36	0.19	0	0.12	unw
SBCL-OFF	NPA-TAC-ON	LMCA Stenosis	0.31	0.25	0.47	0.12	0	0.06	unw
SBCL-OFF	NPA-TAC-ON	Two Vessels Disease	0.29	0.39	0.47	0.2	0	0.09	unw
SBCL-OFF	NPA-TAC-ON	Previuos PTCA	0.3	0.16	0.46	0.29	0	0.14	unw
SBCL-OFF	NPA-TAC-ON	Antiplatelet Drugs	0.32	0.31	0.47	0.02	0.7	0.01	unw
SBCL-OFF	NPA-TAC-ON	Surgery	0.04	0.13	0.28	0.3	0	0.08	unw
SBCL-OFF	NPA-TAC-ON	REDO	0.02	0.05	0.2	0.14	0	0.03	unw
SBCL-OFF	NPA-TAC-ON	NYHA ≥ 3	0.08	0.16	0.36	0.22	0	0.08	unw
SBCL-OFF	NPA-TAC-ON	≥ 3-Vessel Disease	0.71	0.17	0.49	1.11	0	0.54	unw
SBCL-OFF	NPA-TAC-ON	LAD Stenosis	0.99	0.92	0.24	0.27	0	0.07	unw
SBCL-OFF	SINGLE TAC-ON	Age	67.46	65.83	9.53	0.17	0	0.08	unw
SBCL-OFF	SINGLE TAC-ON	Gender(Male)	0.82	0.79	0.4	0.07	0.01	0.03	unw
SBCL-OFF	SINGLE TAC-ON	Hypertension	0.36	0.66	0.49	0.6	0	0.3	unw
SBCL-OFF	SINGLE TAC-ON	Smoker	0.13	0.44	0.48	0.65	0	0.31	unw
SBCL-OFF	SINGLE TAC-ON	Hypercholesterolemia	0.15	0.5	0.5	0.7	0	0.35	unw
SBCL-OFF	SINGLE TAC-ON	Diabetes	0.23	0.19	0.43	0.1	0	0.04	unw
SBCL-OFF	SINGLE TAC-ON	Insulin Dependent Diabetes	0.03	0.11	0.29	0.29	0	0.08	unw
SBCL-OFF	SINGLE TAC-ON	COPD	0.05	0.09	0.25	0.15	0	0.04	unw
SBCL-OFF	SINGLE TAC-ON	AKD	0.02	0.05	0.2	0.12	0	0.02	unw
SBCL-OFF	SINGLE TAC-ON	Dialysis	0	0.01	0.09	0.09	0	0.01	unw
SBCL-OFF	SINGLE TAC-ON	Stroke History	0.01	0.07	0.17	0.33	0	0.06	unw
SBCL-OFF	SINGLE TAC-ON	TIA History	0.01	0.04	0.17	0.17	0	0.03	unw
SBCL-OFF	SINGLE TAC-ON	Carotid Stenosis > 50%	0.06	0.16	0.27	0.39	0	0.1	unw
SBCL-OFF	SINGLE TAC-ON	PAD	0.06	0.24	0.38	0.48	0	0.18	unw
SBCL-OFF	SINGLE TAC-ON	Pulmonary Hypertension	0.03	0.1	0.21	0.33	0	0.07	unw
SBCL-OFF	SINGLE TAC-ON	IABP Preop	0.02	0.01	0.12	0.08	0	0.01	unw
SBCL-OFF	SINGLE TAC-ON	LVEF	55.09	50.73	11.36	0.38	0	0.25	unw
SBCL-OFF	SINGLE TAC-ON	LMCA Stenosis	0.31	0.39	0.47	0.18	0	0.08	unw
SBCL-OFF	SINGLE TAC-ON	Two Vessels Disease	0.29	0.32	0.47	0.07	0.02	0.03	unw
SBCL-OFF	SINGLE TAC-ON	Previuos PTCA	0.3	0.17	0.46	0.28	0	0.13	unw

SBCL-OFF	SINGLE TAC-ON	Antiplatelet Drugs	0.32	0.33	0.47	0.03	0.25	0.02	unw
SBCL-OFF	SINGLE TAC-ON	Surgery	0.04	0.15	0.28	0.4	0	0.11	unw
SBCL-OFF	SINGLE TAC-ON	REDO	0.02	0.08	0.2	0.32	0	0.06	unw
SBCL-OFF	SINGLE TAC-ON	NYHA ≥ 3	0.08	0.08	0.36	0.01	0.68	0	unw
SBCL-OFF	SINGLE TAC-ON	≥ 3-Vessel Disease	0.71	0.72	0.49	0.02	0.51	0.01	unw
SBCL-OFF	SINGLE TAC-ON	LAD Stenosis	0.99	0.93	0.24	0.26	0	0.06	unw
SBCL-OFF	TAC-SBCL-ON	Age	67.46	67.47	9.53	0	0.97	0.02	unw
SBCL-OFF	TAC-SBCL-ON	Gender(Male)	0.82	0.8	0.4	0.05	0.08	0.02	unw
SBCL-OFF	TAC-SBCL-ON	Hypertension	0.36	0.63	0.49	0.55	0	0.27	unw
SBCL-OFF	TAC-SBCL-ON	Smoker	0.13	0.4	0.48	0.57	0	0.27	unw
SBCL-OFF	TAC-SBCL-ON	Hypercholesterolemia	0.15	0.5	0.5	0.7	0	0.34	unw
SBCL-OFF	TAC-SBCL-ON	Diabetes	0.23	0.28	0.43	0.12	0	0.05	unw
SBCL-OFF	TAC-SBCL-ON	Insulin Dependent Diabetes	0.03	0.09	0.29	0.21	0	0.06	unw
SBCL-OFF	TAC-SBCL-ON	COPD	0.05	0.07	0.25	0.08	0	0.02	unw
SBCL-OFF	TAC-SBCL-ON	AKD	0.02	0.05	0.2	0.16	0	0.03	unw
SBCL-OFF	TAC-SBCL-ON	Dialysis	0	0.01	0.09	0.08	0	0.01	unw
SBCL-OFF	TAC-SBCL-ON	Stroke History	0.01	0.02	0.17	0.06	0	0.01	unw
SBCL-OFF	TAC-SBCL-ON	TIA History	0.01	0.04	0.17	0.17	0	0.03	unw
SBCL-OFF	TAC-SBCL-ON	Carotid Stenosis > 50%	0.06	0.04	0.27	0.06	0.01	0.02	unw
SBCL-OFF	TAC-SBCL-ON	PAD	0.06	0.18	0.38	0.31	0	0.12	unw
SBCL-OFF	TAC-SBCL-ON	Pulmonary Hypertension	0.03	0.04	0.21	0.06	0	0.01	unw
SBCL-OFF	TAC-SBCL-ON	IABP Preop	0.02	0.02	0.12	0	0.97	0	unw
SBCL-OFF	TAC-SBCL-ON	LVEF	55.09	52.04	11.36	0.27	0	0.13	unw
SBCL-OFF	TAC-SBCL-ON	LMCA Stenosis	0.31	0.33	0.47	0.05	0.05	0.02	unw
SBCL-OFF	TAC-SBCL-ON	Two Vessels Disease	0.29	0.34	0.47	0.11	0	0.05	unw
SBCL-OFF	TAC-SBCL-ON	Previuos PTCA	0.3	0.54	0.46	0.52	0	0.24	unw
SBCL-OFF	TAC-SBCL-ON	Antiplatelet Drugs	0.32	0.33	0.47	0.02	0.44	0.01	unw
SBCL-OFF	TAC-SBCL-ON	Surgery	0.04	0.07	0.28	0.08	0	0.02	unw
SBCL-OFF	TAC-SBCL-ON	REDO	0.02	0.03	0.2	0.04	0.02	0.01	unw
SBCL-OFF	TAC-SBCL-ON	NYHA ≥ 3	0.08	0.2	0.36	0.33	0	0.12	unw
SBCL-OFF	TAC-SBCL-ON	≥ 3-Vessel Disease	0.71	0.65	0.49	0.12	0	0.06	unw
SBCL-OFF	TAC-SBCL-ON	LAD Stenosis	0.99	0.94	0.24	0.19	0	0.05	unw

NPA-BHON	SBCL-BHON	Age	68.8	66.6	9.53	0.23	0	0.09	unw
NPA-BHON	SBCL-BHON	Gender(Male)	0.83	0.82	0.4	0.02	0.7	0.01	unw
NPA-BHON	SBCL-BHON	Hypertension	0.32	0.8	0.49	0.98	0	0.49	unw
NPA-BHON	SBCL-BHON	Smoker	0.12	0.54	0.48	0.89	0	0.43	unw
NPA-BHON	SBCL-BHON	Hypercholesterolemia	0.13	0.73	0.5	1.2	0	0.59	unw
NPA-BHON	SBCL-BHON	Diabetes	0.2	0.37	0.43	0.38	0	0.16	unw
NPA-BHON	SBCL-BHON	Insulin Dependent Diabetes	0.03	0.2	0.29	0.61	0	0.17	unw
NPA-BHON	SBCL-BHON	COPD	0.09	0.02	0.25	0.27	0	0.07	unw
NPA-BHON	SBCL-BHON	AKD	0.05	0.02	0.2	0.15	0	0.03	unw
NPA-BHON	SBCL-BHON	Dialysis	0.01	0.01	0.09	0.01	0.85	0	unw
NPA-BHON	SBCL-BHON	Stroke History	0.02	0.01	0.17	0.08	0.01	0.01	unw
NPA-BHON	SBCL-BHON	TIA History	0.02	0.02	0.17	0.01	0.74	0	unw
NPA-BHON	SBCL-BHON	Carotid Stenosis > 50%	0.13	0.02	0.27	0.4	0	0.11	unw
NPA-BHON	SBCL-BHON	PAD	0.08	0.24	0.38	0.43	0	0.16	unw
NPA-BHON	SBCL-BHON	Pulmonary Hypertension	0.04	0.01	0.21	0.12	0	0.03	unw
NPA-BHON	SBCL-BHON	IABP Preop	0.01	0.03	0.12	0.18	0	0.02	unw
NPA-BHON	SBCL-BHON	LVEF	55.65	52.22	11.36	0.3	0	0.24	unw
NPA-BHON	SBCL-BHON	LMCA Stenosis	0.34	0.35	0.47	0.01	0.83	0	unw
NPA-BHON	SBCL-BHON	Two Vessels Disease	0.55	0.22	0.47	0.69	0	0.33	unw
NPA-BHON	SBCL-BHON	Previuos PTCA	0.09	0.16	0.46	0.15	0	0.07	unw
NPA-BHON	SBCL-BHON	Antiplatelet Drugs	0.29	0.31	0.47	0.04	0.3	0.02	unw
NPA-BHON	SBCL-BHON	Surgery	0.05	0.06	0.28	0.06	0.07	0.02	unw
NPA-BHON	SBCL-BHON	REDO	0.04	0	0.2	0.2	0	0.04	unw
NPA-BHON	SBCL-BHON	NYHA ≥ 3	0.05	0.29	0.36	0.66	0	0.24	unw
NPA-BHON	SBCL-BHON	≥ 3-Vessel Disease	0.15	0.77	0.49	1.25	0	0.61	unw
NPA-BHON	SBCL-BHON	LAD Stenosis	0.97	1	0.24	0.12	0	0.03	unw
NPA-BHON	NPA-TAC-ON	Age	68.8	66.15	9.53	0.28	0	0.12	unw
NPA-BHON	NPA-TAC-ON	Gender(Male)	0.83	0.74	0.4	0.22	0	0.09	unw
NPA-BHON	NPA-TAC-ON	Hypertension	0.32	0.49	0.49	0.35	0	0.17	unw
NPA-BHON	NPA-TAC-ON	Smoker	0.12	0.29	0.48	0.37	0	0.18	unw
NPA-BHON	NPA-TAC-ON	Hypercholesterolemia	0.13	0.37	0.5	0.47	0	0.23	unw
NPA-BHON	NPA-TAC-ON	Diabetes	0.2	0.22	0.43	0.03	0.44	0.02	unw

NPA-BHON	NPA-TAC-ON	Insulin Dependent Diabetes	0.03	0.08	0.29	0.18	0	0.05	unw
NPA-BHON	NPA-TAC-ON	COPD	0.09	0.07	0.25	0.06	0.23	0.02	unw
NPA-BHON	NPA-TAC-ON	AKD	0.05	0.05	0.2	0	0.96	0	unw
NPA-BHON	NPA-TAC-ON	Dialysis	0.01	0.01	0.09	0	0.99	0	unw
NPA-BHON	NPA-TAC-ON	Stroke History	0.02	0.03	0.17	0.07	0.1	0.01	unw
NPA-BHON	NPA-TAC-ON	TIA History	0.02	0.02	0.17	0.02	0.6	0	unw
NPA-BHON	NPA-TAC-ON	Carotid Stenosis > 50%	0.13	0.05	0.27	0.28	0	0.07	unw
NPA-BHON	NPA-TAC-ON	PAD	0.08	0.15	0.38	0.21	0	0.08	unw
NPA-BHON	NPA-TAC-ON	Pulmonary Hypertension	0.04	0.04	0.21	0.03	0.55	0.01	unw
NPA-BHON	NPA-TAC-ON	IABP Preop	0.01	0.01	0.12	0.02	0.71	0	unw
NPA-BHON	NPA-TAC-ON	LVEF	55.65	52.96	11.36	0.24	0	0.17	unw
NPA-BHON	NPA-TAC-ON	LMCA Stenosis	0.34	0.25	0.47	0.2	0	0.09	unw
NPA-BHON	NPA-TAC-ON	Two Vessels Disease	0.55	0.39	0.47	0.34	0	0.16	unw
NPA-BHON	NPA-TAC-ON	Previuos PTCA	0.09	0.16	0.46	0.14	0	0.07	unw
NPA-BHON	NPA-TAC-ON	Antiplatelet Drugs	0.29	0.31	0.47	0.04	0.42	0.02	unw
NPA-BHON	NPA-TAC-ON	Surgery	0.05	0.13	0.28	0.29	0	0.08	unw
NPA-BHON	NPA-TAC-ON	REDO	0.04	0.05	0.2	0.03	0.5	0.01	unw
NPA-BHON	NPA-TAC-ON	NYHA ≥ 3	0.05	0.16	0.36	0.3	0	0.11	unw
NPA-BHON	NPA-TAC-ON	≥ 3-Vessel Disease	0.15	0.17	0.49	0.03	0.46	0.01	unw
NPA-BHON	NPA-TAC-ON	LAD Stenosis	0.97	0.92	0.24	0.18	0	0.04	unw
NPA-BHON	SINGLE TAC-ON	Age	68.8	65.83	9.53	0.31	0	0.14	unw
NPA-BHON	SINGLE TAC-ON	Gender(Male)	0.83	0.79	0.4	0.09	0.01	0.04	unw
NPA-BHON	SINGLE TAC-ON	Hypertension	0.32	0.66	0.49	0.69	0	0.34	unw
NPA-BHON	SINGLE TAC-ON	Smoker	0.12	0.44	0.48	0.68	0	0.32	unw
NPA-BHON	SINGLE TAC-ON	Hypercholesterolemia	0.13	0.5	0.5	0.74	0	0.37	unw
NPA-BHON	SINGLE TAC-ON	Diabetes	0.2	0.19	0.43	0.04	0.25	0.02	unw
NPA-BHON	SINGLE TAC-ON	Insulin Dependent Diabetes	0.03	0.11	0.29	0.3	0	0.09	unw
NPA-BHON	SINGLE TAC-ON	COPD	0.09	0.09	0.25	0.01	0.89	0	unw
NPA-BHON	SINGLE TAC-ON	AKD	0.05	0.05	0.2	0	0.97	0	unw
NPA-BHON	SINGLE TAC-ON	Dialysis	0.01	0.01	0.09	0	0.92	0	unw
NPA-BHON	SINGLE TAC-ON	Stroke History	0.02	0.07	0.17	0.29	0	0.05	unw
NPA-BHON	SINGLE TAC-ON	TIA History	0.02	0.04	0.17	0.13	0	0.02	unw

NPA-BHON	SINGLE TAC-ON	Carotid Stenosis > 50%	0.13	0.16	0.27	0.14	0	0.04	unw
NPA-BHON	SINGLE TAC-ON	PAD	0.08	0.24	0.38	0.44	0	0.17	unw
NPA-BHON	SINGLE TAC-ON	Pulmonary Hypertension	0.04	0.1	0.21	0.27	0	0.06	unw
NPA-BHON	SINGLE TAC-ON	IABP Preop	0.01	0.01	0.12	0.04	0.18	0.01	unw
NPA-BHON	SINGLE TAC-ON	LVEF	55.65	50.73	11.36	0.43	0	0.3	unw
NPA-BHON	SINGLE TAC-ON	LMCA Stenosis	0.34	0.39	0.47	0.1	0.01	0.05	unw
NPA-BHON	SINGLE TAC-ON	Two Vessels Disease	0.55	0.32	0.47	0.47	0	0.23	unw
NPA-BHON	SINGLE TAC-ON	Previuos PTCA	0.09	0.17	0.46	0.16	0	0.07	unw
NPA-BHON	SINGLE TAC-ON	Antiplatelet Drugs	0.29	0.33	0.47	0.09	0.02	0.04	unw
NPA-BHON	SINGLE TAC-ON	Surgery	0.05	0.15	0.28	0.39	0	0.11	unw
NPA-BHON	SINGLE TAC-ON	REDO	0.04	0.08	0.2	0.21	0	0.04	unw
NPA-BHON	SINGLE TAC-ON	NYHA ≥ 3	0.05	0.08	0.36	0.09	0	0.03	unw
NPA-BHON	SINGLE TAC-ON	≥ 3-Vessel Disease	0.15	0.72	0.49	1.16	0	0.57	unw
NPA-BHON	SINGLE TAC-ON	LAD Stenosis	0.97	0.93	0.24	0.17	0	0.04	unw
NPA-BHON	TAC-SBCL-ON	Age	68.8	67.47	9.53	0.14	0	0.06	unw
NPA-BHON	TAC-SBCL-ON	Gender(Male)	0.83	0.8	0.4	0.07	0.06	0.03	unw
NPA-BHON	TAC-SBCL-ON	Hypertension	0.32	0.63	0.49	0.64	0	0.31	unw
NPA-BHON	TAC-SBCL-ON	Smoker	0.12	0.4	0.48	0.59	0	0.28	unw
NPA-BHON	TAC-SBCL-ON	Hypercholesterolemia	0.13	0.5	0.5	0.74	0	0.36	unw
NPA-BHON	TAC-SBCL-ON	Diabetes	0.2	0.28	0.43	0.18	0	0.08	unw
NPA-BHON	TAC-SBCL-ON	Insulin Dependent Diabetes	0.03	0.09	0.29	0.21	0	0.06	unw
NPA-BHON	TAC-SBCL-ON	COPD	0.09	0.07	0.25	0.08	0.05	0.02	unw
NPA-BHON	TAC-SBCL-ON	AKD	0.05	0.05	0.2	0.04	0.29	0.01	unw
NPA-BHON	TAC-SBCL-ON	Dialysis	0.01	0.01	0.09	0.01	0.75	0	unw
NPA-BHON	TAC-SBCL-ON	Stroke History	0.02	0.02	0.17	0.01	0.73	0	unw
NPA-BHON	TAC-SBCL-ON	TIA History	0.02	0.04	0.17	0.13	0	0.02	unw
NPA-BHON	TAC-SBCL-ON	Carotid Stenosis > 50%	0.13	0.04	0.27	0.31	0	0.08	unw
NPA-BHON	TAC-SBCL-ON	PAD	0.08	0.18	0.38	0.27	0	0.1	unw
NPA-BHON	TAC-SBCL-ON	Pulmonary Hypertension	0.04	0.04	0.21	0	0.95	0	unw
NPA-BHON	TAC-SBCL-ON	IABP Preop	0.01	0.02	0.12	0.04	0.25	0	unw
NPA-BHON	TAC-SBCL-ON	LVEF	55.65	52.04	11.36	0.32	0	0.18	unw
NPA-BHON	TAC-SBCL-ON	LMCA Stenosis	0.34	0.33	0.47	0.02	0.5	0.01	unw

NPA-BHON	TAC-SBCL-ON	Two Vessels Disease	0.55	0.34	0.47	0.43	0	0.2	unw
NPA-BHON	TAC-SBCL-ON	Previuos PTCA	0.09	0.54	0.46	0.96	0	0.44	unw
NPA-BHON	TAC-SBCL-ON	Antiplatelet Drugs	0.29	0.33	0.47	0.07	0.04	0.03	unw
NPA-BHON	TAC-SBCL-ON	Surgery	0.05	0.07	0.28	0.08	0.01	0.02	unw
NPA-BHON	TAC-SBCL-ON	REDO	0.04	0.03	0.2	0.07	0.07	0.01	unw
NPA-BHON	TAC-SBCL-ON	NYHA ≥ 3	0.05	0.2	0.36	0.41	0	0.15	unw
NPA-BHON	TAC-SBCL-ON	≥ 3-Vessel Disease	0.15	0.65	0.49	1.02	0	0.5	unw
NPA-BHON	TAC-SBCL-ON	LAD Stenosis	0.97	0.94	0.24	0.1	0	0.02	unw
SBCL-BHON	NPA-TAC-ON	Age	66.6	66.15	9.53	0.05	0.29	0.03	unw
SBCL-BHON	NPA-TAC-ON	Gender(Male)	0.82	0.74	0.4	0.2	0	0.08	unw
SBCL-BHON	NPA-TAC-ON	Hypertension	0.8	0.49	0.49	0.64	0	0.31	unw
SBCL-BHON	NPA-TAC-ON	Smoker	0.54	0.29	0.48	0.53	0	0.25	unw
SBCL-BHON	NPA-TAC-ON	Hypercholesterolemia	0.73	0.37	0.5	0.72	0	0.36	unw
SBCL-BHON	NPA-TAC-ON	Diabetes	0.37	0.22	0.43	0.34	0	0.15	unw
SBCL-BHON	NPA-TAC-ON	Insulin Dependent Diabetes	0.2	0.08	0.29	0.43	0	0.12	unw
SBCL-BHON	NPA-TAC-ON	COPD	0.02	0.07	0.25	0.21	0	0.05	unw
SBCL-BHON	NPA-TAC-ON	AKD	0.02	0.05	0.2	0.15	0	0.03	unw
SBCL-BHON	NPA-TAC-ON	Dialysis	0.01	0.01	0.09	0.01	0.84	0	unw
SBCL-BHON	NPA-TAC-ON	Stroke History	0.01	0.03	0.17	0.15	0	0.02	unw
SBCL-BHON	NPA-TAC-ON	TIA History	0.02	0.02	0.17	0.03	0.35	0.01	unw
SBCL-BHON	NPA-TAC-ON	Carotid Stenosis > 50%	0.02	0.05	0.27	0.13	0	0.03	unw
SBCL-BHON	NPA-TAC-ON	PAD	0.24	0.15	0.38	0.22	0	0.08	unw
SBCL-BHON	NPA-TAC-ON	Pulmonary Hypertension	0.01	0.04	0.21	0.15	0	0.03	unw
SBCL-BHON	NPA-TAC-ON	IABP Preop	0.03	0.01	0.12	0.16	0	0.02	unw
SBCL-BHON	NPA-TAC-ON	LVEF	52.22	52.96	11.36	0.06	0.1	0.13	unw
SBCL-BHON	NPA-TAC-ON	LMCA Stenosis	0.35	0.25	0.47	0.21	0	0.1	unw
SBCL-BHON	NPA-TAC-ON	Two Vessels Disease	0.22	0.39	0.47	0.35	0	0.17	unw
SBCL-BHON	NPA-TAC-ON	Previuos PTCA	0.16	0.16	0.46	0.01	0.82	0	unw
SBCL-BHON	NPA-TAC-ON	Antiplatelet Drugs	0.31	0.31	0.47	0.01	0.88	0	unw
SBCL-BHON	NPA-TAC-ON	Surgery	0.06	0.13	0.28	0.23	0	0.06	unw
SBCL-BHON	NPA-TAC-ON	REDO	0	0.05	0.2	0.23	0	0.05	unw
SBCL-BHON	NPA-TAC-ON	NYHA ≥ 3	0.29	0.16	0.36	0.36	0	0.13	unw

SBCL-BHON	NPA-TAC-ON	≥ 3-Vessel Disease	0.77	0.17	0.49	1.22	0	0.6	unw
SBCL-BHON	NPA-TAC-ON	LAD Stenosis	1	0.92	0.24	0.3	0	0.07	unw
SBCL-BHON	SINGLE TAC-ON	Age	66.6	65.83	9.53	0.08	0.01	0.05	unw
SBCL-BHON	SINGLE TAC-ON	Gender(Male)	0.82	0.79	0.4	0.07	0.01	0.03	unw
SBCL-BHON	SINGLE TAC-ON	Hypertension	0.8	0.66	0.49	0.29	0	0.15	unw
SBCL-BHON	SINGLE TAC-ON	Smoker	0.54	0.44	0.48	0.22	0	0.1	unw
SBCL-BHON	SINGLE TAC-ON	Hypercholesterolemia	0.73	0.5	0.5	0.46	0	0.23	unw
SBCL-BHON	SINGLE TAC-ON	Diabetes	0.37	0.19	0.43	0.42	0	0.18	unw
SBCL-BHON	SINGLE TAC-ON	Insulin Dependent Diabetes	0.2	0.11	0.29	0.31	0	0.09	unw
SBCL-BHON	SINGLE TAC-ON	COPD	0.02	0.09	0.25	0.27	0	0.07	unw
SBCL-BHON	SINGLE TAC-ON	AKD	0.02	0.05	0.2	0.14	0	0.03	unw
SBCL-BHON	SINGLE TAC-ON	Dialysis	0.01	0.01	0.09	0.01	0.7	0	unw
SBCL-BHON	SINGLE TAC-ON	Stroke History	0.01	0.07	0.17	0.36	0	0.06	unw
SBCL-BHON	SINGLE TAC-ON	TIA History	0.02	0.04	0.17	0.14	0	0.02	unw
SBCL-BHON	SINGLE TAC-ON	Carotid Stenosis > 50%	0.02	0.16	0.27	0.54	0	0.15	unw
SBCL-BHON	SINGLE TAC-ON	PAD	0.24	0.24	0.38	0.01	0.81	0	unw
SBCL-BHON	SINGLE TAC-ON	Pulmonary Hypertension	0.01	0.1	0.21	0.39	0	0.08	unw
SBCL-BHON	SINGLE TAC-ON	IABP Preop	0.03	0.01	0.12	0.22	0	0.03	unw
SBCL-BHON	SINGLE TAC-ON	LVEF	52.22	50.73	11.36	0.13	0	0.19	unw
SBCL-BHON	SINGLE TAC-ON	LMCA Stenosis	0.35	0.39	0.47	0.09	0	0.04	unw
SBCL-BHON	SINGLE TAC-ON	Two Vessels Disease	0.22	0.32	0.47	0.22	0	0.1	unw
SBCL-BHON	SINGLE TAC-ON	Previuos PTCA	0.16	0.17	0.46	0	0.9	0	unw
SBCL-BHON	SINGLE TAC-ON	Antiplatelet Drugs	0.31	0.33	0.47	0.04	0.16	0.02	unw
SBCL-BHON	SINGLE TAC-ON	Surgery	0.06	0.15	0.28	0.33	0	0.09	unw
SBCL-BHON	SINGLE TAC-ON	REDO	0	0.08	0.2	0.41	0	0.08	unw
SBCL-BHON	SINGLE TAC-ON	NYHA ≥ 3	0.29	0.08	0.36	0.58	0	0.21	unw
SBCL-BHON	SINGLE TAC-ON	≥ 3-Vessel Disease	0.77	0.72	0.49	0.09	0	0.04	unw
SBCL-BHON	SINGLE TAC-ON	LAD Stenosis	1	0.93	0.24	0.29	0	0.07	unw
SBCL-BHON	TAC-SBCL-ON	Age	66.6	67.47	9.53	0.09	0	0.04	unw
SBCL-BHON	TAC-SBCL-ON	Gender(Male)	0.82	0.8	0.4	0.05	0.07	0.02	unw
SBCL-BHON	TAC-SBCL-ON	Hypertension	0.8	0.63	0.49	0.35	0	0.17	unw
SBCL-BHON	TAC-SBCL-ON	Smoker	0.54	0.4	0.48	0.3	0	0.14	unw

SBCL-BHON	TAC-SBCL-ON	Hypercholesterolemia	0.73	0.5	0.5	0.46	0	0.23	unw
SBCL-BHON	TAC-SBCL-ON	Diabetes	0.37	0.28	0.43	0.19	0	0.08	unw
SBCL-BHON	TAC-SBCL-ON	Insulin Dependent Diabetes	0.2	0.09	0.29	0.4	0	0.12	unw
SBCL-BHON	TAC-SBCL-ON	COPD	0.02	0.07	0.25	0.19	0	0.05	unw
SBCL-BHON	TAC-SBCL-ON	AKD	0.02	0.05	0.2	0.19	0	0.04	unw
SBCL-BHON	TAC-SBCL-ON	Dialysis	0.01	0.01	0.09	0.02	0.5	0	unw
SBCL-BHON	TAC-SBCL-ON	Stroke History	0.01	0.02	0.17	0.09	0	0.01	unw
SBCL-BHON	TAC-SBCL-ON	TIA History	0.02	0.04	0.17	0.14	0	0.02	unw
SBCL-BHON	TAC-SBCL-ON	Carotid Stenosis > 50%	0.02	0.04	0.27	0.1	0	0.03	unw
SBCL-BHON	TAC-SBCL-ON	PAD	0.24	0.18	0.38	0.16	0	0.06	unw
SBCL-BHON	TAC-SBCL-ON	Pulmonary Hypertension	0.01	0.04	0.21	0.12	0	0.03	unw
SBCL-BHON	TAC-SBCL-ON	IABP Preop	0.03	0.02	0.12	0.14	0	0.02	unw
SBCL-BHON	TAC-SBCL-ON	LVEF	52.22	52.04	11.36	0.02	0.5	0.11	unw
SBCL-BHON	TAC-SBCL-ON	LMCA Stenosis	0.35	0.33	0.47	0.03	0.24	0.02	unw
SBCL-BHON	TAC-SBCL-ON	Two Vessels Disease	0.22	0.34	0.47	0.26	0	0.12	unw
SBCL-BHON	TAC-SBCL-ON	Previuos PTCA	0.16	0.54	0.46	0.81	0	0.37	unw
SBCL-BHON	TAC-SBCL-ON	Antiplatelet Drugs	0.31	0.33	0.47	0.03	0.29	0.01	unw
SBCL-BHON	TAC-SBCL-ON	Surgery	0.06	0.07	0.28	0.01	0.56	0	unw
SBCL-BHON	TAC-SBCL-ON	REDO	0	0.03	0.2	0.13	0	0.03	unw
SBCL-BHON	TAC-SBCL-ON	NYHA ≥ 3	0.29	0.2	0.36	0.25	0	0.09	unw
SBCL-BHON	TAC-SBCL-ON	≥ 3-Vessel Disease	0.77	0.65	0.49	0.23	0	0.11	unw
SBCL-BHON	TAC-SBCL-ON	LAD Stenosis	1	0.94	0.24	0.22	0	0.05	unw
NPA-TAC-ON	SINGLE TAC-ON	Age	66.15	65.83	9.53	0.03	0.37	0.06	unw
NPA-TAC-ON	SINGLE TAC-ON	Gender(Male)	0.74	0.79	0.4	0.13	0	0.05	unw
NPA-TAC-ON	SINGLE TAC-ON	Hypertension	0.49	0.66	0.49	0.34	0	0.17	unw
NPA-TAC-ON	SINGLE TAC-ON	Smoker	0.29	0.44	0.48	0.31	0	0.15	unw
NPA-TAC-ON	SINGLE TAC-ON	Hypercholesterolemia	0.37	0.5	0.5	0.27	0	0.13	unw
NPA-TAC-ON	SINGLE TAC-ON	Diabetes	0.22	0.19	0.43	0.08	0.03	0.03	unw
NPA-TAC-ON	SINGLE TAC-ON	Insulin Dependent Diabetes	0.08	0.11	0.29	0.12	0	0.03	unw
NPA-TAC-ON	SINGLE TAC-ON	COPD	0.07	0.09	0.25	0.06	0.14	0.01	unw
NPA-TAC-ON	SINGLE TAC-ON	AKD	0.05	0.05	0.2	0	0.91	0	unw
NPA-TAC-ON	SINGLE TAC-ON	Dialysis	0.01	0.01	0.09	0	0.93	0	unw

NPA-TAC-ON	SINGLE TAC-ON	Stroke History	0.03	0.07	0.17	0.21	0	0.04	unw
NPA-TAC-ON	SINGLE TAC-ON	TIA History	0.02	0.04	0.17	0.11	0	0.02	unw
NPA-TAC-ON	SINGLE TAC-ON	Carotid Stenosis > 50%	0.05	0.16	0.27	0.42	0	0.11	unw
NPA-TAC-ON	SINGLE TAC-ON	PAD	0.15	0.24	0.38	0.23	0	0.09	unw
NPA-TAC-ON	SINGLE TAC-ON	Pulmonary Hypertension	0.04	0.1	0.21	0.24	0	0.05	unw
NPA-TAC-ON	SINGLE TAC-ON	IABP Preop	0.01	0.01	0.12	0.06	0.07	0.01	unw
NPA-TAC-ON	SINGLE TAC-ON	LVEF	52.96	50.73	11.36	0.2	0	0.13	unw
NPA-TAC-ON	SINGLE TAC-ON	LMCA Stenosis	0.25	0.39	0.47	0.3	0	0.14	unw
NPA-TAC-ON	SINGLE TAC-ON	Two Vessels Disease	0.39	0.32	0.47	0.13	0	0.06	unw
NPA-TAC-ON	SINGLE TAC-ON	Previuos PTCA	0.16	0.17	0.46	0.01	0.72	0	unw
NPA-TAC-ON	SINGLE TAC-ON	Antiplatelet Drugs	0.31	0.33	0.47	0.05	0.18	0.02	unw
NPA-TAC-ON	SINGLE TAC-ON	Surgery	0.13	0.15	0.28	0.1	0.02	0.03	unw
NPA-TAC-ON	SINGLE TAC-ON	REDO	0.05	0.08	0.2	0.18	0	0.03	unw
NPA-TAC-ON	SINGLE TAC-ON	NYHA ≥ 3	0.16	0.08	0.36	0.21	0	0.08	unw
NPA-TAC-ON	SINGLE TAC-ON	≥ 3-Vessel Disease	0.17	0.72	0.49	1.13	0	0.55	unw
NPA-TAC-ON	SINGLE TAC-ON	LAD Stenosis	0.92	0.93	0.24	0.01	0.75	0	unw
NPA-TAC-ON	TAC-SBCL-ON	Age	66.15	67.47	9.53	0.14	0	0.07	unw
NPA-TAC-ON	TAC-SBCL-ON	Gender(Male)	0.74	0.8	0.4	0.15	0	0.06	unw
NPA-TAC-ON	TAC-SBCL-ON	Hypertension	0.49	0.63	0.49	0.29	0	0.14	unw
NPA-TAC-ON	TAC-SBCL-ON	smoker	0.29	0.4	0.48	0.22	0	0.11	unw
NPA-TAC-ON	TAC-SBCL-ON	Hypercholesterolemia	0.37	0.5	0.5	0.26	0	0.13	unw
NPA-TAC-ON	TAC-SBCL-ON	Diabetes	0.22	0.28	0.43	0.15	0	0.06	unw
NPA-TAC-ON	TAC-SBCL-ON	Insulin Dependent Diabetes	0.08	0.09	0.29	0.03	0.39	0.01	unw
NPA-TAC-ON	TAC-SBCL-ON	COPD	0.07	0.07	0.25	0.02	0.64	0	unw
NPA-TAC-ON	TAC-SBCL-ON	AKD	0.05	0.05	0.2	0.04	0.3	0.01	unw
NPA-TAC-ON	TAC-SBCL-ON	Dialysis	0.01	0.01	0.09	0.01	0.75	0	unw
NPA-TAC-ON	TAC-SBCL-ON	Stroke History	0.03	0.02	0.17	0.06	0.08	0.01	unw
NPA-TAC-ON	TAC-SBCL-ON	TIA History	0.02	0.04	0.17	0.11	0	0.02	unw
NPA-TAC-ON	TAC-SBCL-ON	Carotid Stenosis > 50%	0.05	0.04	0.27	0.03	0.26	0.01	unw
NPA-TAC-ON	TAC-SBCL-ON	PAD	0.15	0.18	0.38	0.07	0.05	0.03	unw
NPA-TAC-ON	TAC-SBCL-ON	Pulmonary Hypertension	0.04	0.04	0.21	0.02	0.47	0.01	unw
NPA-TAC-ON	TAC-SBCL-ON	IABP Preop	0.01	0.02	0.12	0.02	0.51	0	unw

NPA-TAC-ON	TAC-SBCL-ON	LVEF	52.96	52.04	11.36	0.08	0.02	0.05	unw
NPA-TAC-ON	TAC-SBCL-ON	LMCA Stenosis	0.25	0.33	0.47	0.17	0	0.08	unw
NPA-TAC-ON	TAC-SBCL-ON	Two Vessels Disease	0.39	0.34	0.47	0.09	0.02	0.04	unw
NPA-TAC-ON	TAC-SBCL-ON	Previuos PTCA	0.16	0.54	0.46	0.81	0	0.38	unw
NPA-TAC-ON	TAC-SBCL-ON	Antiplatelet Drugs	0.31	0.33	0.47	0.04	0.3	0.02	unw
NPA-TAC-ON	TAC-SBCL-ON	Surgery	0.13	0.07	0.28	0.21	0	0.06	unw
NPA-TAC-ON	TAC-SBCL-ON	REDO	0.05	0.03	0.2	0.1	0.01	0.02	unw
NPA-TAC-ON	TAC-SBCL-ON	NYHA ≥ 3	0.16	0.2	0.36	0.11	0	0.04	unw
NPA-TAC-ON	TAC-SBCL-ON	≥ 3-Vessel Disease	0.17	0.65	0.49	0.99	0	0.49	unw
NPA-TAC-ON	TAC-SBCL-ON	LAD Stenosis	0.92	0.94	0.24	0.08	0.03	0.02	unw
SINGLE TAC-ON	TAC-SBCL-ON	Age	65.83	67.47	9.53	0.17	0	0.08	unw
SINGLE TAC-ON	TAC-SBCL-ON	Gender(Male)	0.79	0.8	0.4	0.02	0.26	0.01	unw
SINGLE TAC-ON	TAC-SBCL-ON	Hypertension	0.66	0.63	0.49	0.05	0.01	0.03	unw
SINGLE TAC-ON	TAC-SBCL-ON	Smoker	0.44	0.4	0.48	0.08	0	0.04	unw
SINGLE TAC-ON	TAC-SBCL-ON	Hypercholesterolemia	0.5	0.5	0.5	0	0.83	0	unw
SINGLE TAC-ON	TAC-SBCL-ON	Diabetes	0.19	0.28	0.43	0.22	0	0.1	unw
SINGLE TAC-ON	TAC-SBCL-ON	Insulin Dependent Diabetes	0.11	0.09	0.29	0.09	0	0.03	unw
SINGLE TAC-ON	TAC-SBCL-ON	COPD	0.09	0.07	0.25	0.07	0	0.02	unw
SINGLE TAC-ON	TAC-SBCL-ON	AKD	0.05	0.05	0.2	0.04	0.05	0.01	unw
SINGLE TAC-ON	TAC-SBCL-ON	Dialysis	0.01	0.01	0.09	0.01	0.69	0	unw
SINGLE TAC-ON	TAC-SBCL-ON	Stroke History	0.07	0.02	0.17	0.28	0	0.05	unw
SINGLE TAC-ON	TAC-SBCL-ON	TIA History	0.04	0.04	0.17	0	0.93	0	unw
SINGLE TAC-ON	TAC-SBCL-ON	Carotid Stenosis > 50%	0.16	0.04	0.27	0.45	0	0.12	unw
SINGLE TAC-ON	TAC-SBCL-ON	PAD	0.24	0.18	0.38	0.16	0	0.06	unw
SINGLE TAC-ON	TAC-SBCL-ON	Pulmonary Hypertension	0.1	0.04	0.21	0.27	0	0.06	unw
SINGLE TAC-ON	TAC-SBCL-ON	IABP Preop	0.01	0.02	0.12	0.08	0	0.01	unw
SINGLE TAC-ON	TAC-SBCL-ON	LVEF	50.73	52.04	11.36	0.12	0	0.13	unw
SINGLE TAC-ON	TAC-SBCL-ON	LMCA Stenosis	0.39	0.33	0.47	0.13	0	0.06	unw
SINGLE TAC-ON	TAC-SBCL-ON	Two Vessels Disease	0.32	0.34	0.47	0.04	0.03	0.02	unw
SINGLE TAC-ON	TAC-SBCL-ON	Previuos PTCA	0.17	0.54	0.46	0.8	0	0.37	unw
SINGLE TAC-ON	TAC-SBCL-ON	Antiplatelet Drugs	0.33	0.33	0.47	0.01	0.55	0.01	unw
SINGLE TAC-ON	TAC-SBCL-ON	Surgery	0.15	0.07	0.28	0.32	0	0.09	unw

SINGLE TAC-ON	TAC-SBCL-ON	REDO	0.08	0.03	0.2	0.28	0	0.05	unw
SINGLE TAC-ON	TAC-SBCL-ON	NYHA ≥ 3	0.08	0.2	0.36	0.32	0	0.12	unw
SINGLE TAC-ON	TAC-SBCL-ON	≥ 3-Vessel Disease	0.72	0.65	0.49	0.14	0	0.07	unw
SINGLE TAC-ON	TAC-SBCL-ON	LAD Stenosis	0.93	0.94	0.24	0.07	0	0.02	unw
NPA-OFF	SBCL-OFF	Age	66.2	67.21	9.53	0.11	0.13	0.06	wgt
NPA-OFF	SBCL-OFF	Gender(Male)	0.82	0.8	0.4	0.04	0.59	0.02	wgt
NPA-OFF	SBCL-OFF	Hypertension	0.63	0.62	0.49	0.02	0.74	0.01	wgt
NPA-OFF	SBCL-OFF	Smoker	0.38	0.36	0.48	0.05	0.48	0.02	wgt
NPA-OFF	SBCL-OFF	Hypercholesterolemia	0.45	0.43	0.5	0.04	0.57	0.02	wgt
NPA-OFF	SBCL-OFF	Diabetes	0.3	0.25	0.43	0.1	0.15	0.04	wgt
NPA-OFF	SBCL-OFF	Insulin Dependent Diabetes	0.08	0.08	0.29	0.01	0.84	0	wgt
NPA-OFF	SBCL-OFF	COPD	0.12	0.07	0.25	0.11	0.04	0.05	wgt
NPA-OFF	SBCL-OFF	AKD	0.06	0.04	0.2	0.06	0.43	0.01	wgt
NPA-OFF	SBCL-OFF	Dialysis	0.01	0	0.09	0.06	0.35	0.01	wgt
NPA-OFF	SBCL-OFF	Stroke History	0.04	0.02	0.17	0.09	0.22	0.02	wgt
NPA-OFF	SBCL-OFF	TIA History	0.03	0.02	0.17	0.02	0.73	0	wgt
NPA-OFF	SBCL-OFF	Carotid Stenosis > 50%	0.14	0.09	0.27	0.12	0.02	0.06	wgt
NPA-OFF	SBCL-OFF	PAD	0.18	0.17	0.38	0.03	0.71	0.01	wgt
NPA-OFF	SBCL-OFF	Pulmonary Hypertension	0.01	0.05	0.21	0.11	0	0.04	wgt
NPA-OFF	SBCL-OFF	IABP Preop	0.01	0.02	0.12	0.09	0.16	0.01	wgt
NPA-OFF	SBCL-OFF	LVEF	52.84	53.05	11.36	0.02	0.76	0.04	wgt
NPA-OFF	SBCL-OFF	LMCA Stenosis	0.27	0.31	0.47	0.1	0.12	0.05	wgt
NPA-OFF	SBCL-OFF	Two Vessels Disease	0.29	0.39	0.47	0.12	0	0.1	wgt
NPA-OFF	SBCL-OFF	Previuos PTCA	0.27	0.27	0.46	0.01	0.93	0	wgt
NPA-OFF	SBCL-OFF	Antiplatelet Drugs	0.26	0.3	0.47	0.09	0.15	0.04	wgt
NPA-OFF	SBCL-OFF	Surgery	0.05	0.07	0.28	0.07	0.31	0.02	wgt
NPA-OFF	SBCL-OFF	REDO	0.04	0.04	0.2	0.01	0.86	0	wgt
NPA-OFF	SBCL-OFF	NYHA ≥ 3	0.15	0.16	0.36	0.03	0.64	0.01	wgt
NPA-OFF	SBCL-OFF	≥ 3-Vessel Disease	0.59	0.59	0.49	0.01	0.87	0	wgt
NPA-OFF	SBCL-OFF	LAD Stenosis	0.91	0.97	0.24	0.14	0	0.06	wgt
NPA-OFF	NPA-BHON	Age	66.2	66.94	9.53	0.08	0.46	0.08	wgt
NPA-OFF	NPA-BHON	Gender(Male)	0.82	0.75	0.4	0.16	0.19	0.07	wgt

NPA-OFF	NPA-BHON	Hypertension	0.63	0.58	0.49	0.1	0.32	0.05	wgt
NPA-OFF	NPA-BHON	Smoker	0.38	0.3	0.48	0.17	0.14	0.08	wgt
NPA-OFF	NPA-BHON	Hypercholesterolemia	0.45	0.46	0.5	0.02	0.89	0.01	wgt
NPA-OFF	NPA-BHON	Diabetes	0.3	0.26	0.43	0.09	0.44	0.04	wgt
NPA-OFF	NPA-BHON	Insulin Dependent Diabetes	0.08	0.07	0.29	0.04	0.74	0.01	wgt
NPA-OFF	NPA-BHON	COPD	0.12	0.04	0.25	0.14	0	0.07	wgt
NPA-OFF	NPA-BHON	AKD	0.06	0.02	0.2	0.09	0.01	0.04	wgt
NPA-OFF	NPA-BHON	Dialysis	0.01	0	0.09	0.07	0.17	0.01	wgt
NPA-OFF	NPA-BHON	Stroke History	0.04	0.03	0.17	0.06	0.51	0.01	wgt
NPA-OFF	NPA-BHON	TIA History	0.03	0.01	0.17	0.11	0.09	0.02	wgt
NPA-OFF	NPA-BHON	Carotid Stenosis > 50%	0.14	0.04	0.27	0.16	0	0.1	wgt
NPA-OFF	NPA-BHON	PAD	0.18	0.12	0.38	0.15	0.17	0.06	wgt
NPA-OFF	NPA-BHON	Pulmonary Hypertension	0.01	0.05	0.21	0.17	0.05	0.04	wgt
NPA-OFF	NPA-BHON	IABP Preop	0.01	0.01	0.12	0	0.96	0	wgt
NPA-OFF	NPA-BHON	LVEF	52.84	53.79	11.36	0.08	0.33	0.06	wgt
NPA-OFF	NPA-BHON	LMCA Stenosis	0.27	0.25	0.47	0.03	0.75	0.02	wgt
NPA-OFF	NPA-BHON	Two Vessels Disease	0.29	0.37	0.47	0.17	0.07	0.08	wgt
NPA-OFF	NPA-BHON	Previuos PTCA	0.27	0.32	0.46	0.11	0.41	0.05	wgt
NPA-OFF	NPA-BHON	Antiplatelet Drugs	0.26	0.36	0.47	0.17	0.07	0.1	wgt
NPA-OFF	NPA-BHON	Surgery	0.05	0.05	0.28	0	0.96	0	wgt
NPA-OFF	NPA-BHON	REDO	0.04	0.05	0.2	0.03	0.79	0.01	wgt
NPA-OFF	NPA-BHON	NYHA ≥ 3	0.15	0.09	0.36	0.18	0.1	0.06	wgt
NPA-OFF	NPA-BHON	≥ 3-Vessel Disease	0.59	0.51	0.49	0.17	0.11	0.08	wgt
NPA-OFF	NPA-BHON	LAD Stenosis	0.91	0.97	0.24	0.15	0	0.05	wgt
NPA-OFF	SBCL-BHON	Age	66.2	67.48	9.53	0.13	0.18	0.06	wgt
NPA-OFF	SBCL-BHON	Gender(Male)	0.82	0.84	0.4	0.07	0.35	0.03	wgt
NPA-OFF	SBCL-BHON	Hypertension	0.63	0.65	0.49	0.04	0.71	0.02	wgt
NPA-OFF	SBCL-BHON	Smoker	0.38	0.4	0.48	0.03	0.73	0.01	wgt
NPA-OFF	SBCL-BHON	Hypercholesterolemia	0.45	0.5	0.5	0.11	0.25	0.05	wgt
NPA-OFF	SBCL-BHON	Diabetes	0.3	0.25	0.43	0.1	0.22	0.04	wgt
NPA-OFF	SBCL-BHON	Insulin Dependent Diabetes	0.08	0.1	0.29	0.07	0.27	0.02	wgt
NPA-OFF	SBCL-BHON	COPD	0.12	0.06	0.25	0.14	0.04	0.06	wgt

NPA-OFF	SBCL-BHON	AKD	0.06	0.03	0.2	0.14	0.17	0.03	wgt
NPA-OFF	SBCL-BHON	Dialysis	0.01	0	0.09	0.05	0.33	0	wgt
NPA-OFF	SBCL-BHON	Stroke History	0.04	0.01	0.17	0.17	0.04	0.03	wgt
NPA-OFF	SBCL-BHON	TIA History	0.03	0.01	0.17	0.1	0.11	0.02	wgt
NPA-OFF	SBCL-BHON	Carotid Stenosis > 50%	0.14	0.03	0.27	0.14	0	0.12	wgt
NPA-OFF	SBCL-BHON	PAD	0.18	0.17	0.38	0.01	0.91	0	wgt
NPA-OFF	SBCL-BHON	Pulmonary Hypertension	0.01	0.04	0.21	0.1	0.2	0.02	wgt
NPA-OFF	SBCL-BHON	IABP Preop	0.01	0.01	0.12	0.01	0.88	0	wgt
NPA-OFF	SBCL-BHON	LVEF	52.84	52.71	11.36	0.01	0.88	0.04	wgt
NPA-OFF	SBCL-BHON	LMCA Stenosis	0.27	0.37	0.47	0.13	0.04	0.1	wgt
NPA-OFF	SBCL-BHON	Two Vessels Disease	0.29	0.33	0.47	0.08	0.29	0.04	wgt
NPA-OFF	SBCL-BHON	Previuos PTCA	0.27	0.28	0.46	0.02	0.85	0.01	wgt
NPA-OFF	SBCL-BHON	Antiplatelet Drugs	0.26	0.35	0.47	0.18	0.03	0.09	wgt
NPA-OFF	SBCL-BHON	Surgery	0.05	0.06	0.28	0.01	0.89	0	wgt
NPA-OFF	SBCL-BHON	REDO	0.04	0	0.2	0.09	0	0.04	wgt
NPA-OFF	SBCL-BHON	NYHA ≥ 3	0.15	0.16	0.36	0.04	0.65	0.01	wgt
NPA-OFF	SBCL-BHON	≥ 3-Vessel Disease	0.59	0.59	0.49	0.01	0.89	0.01	wgt
NPA-OFF	SBCL-BHON	LAD Stenosis	0.91	0.93	0.24	0.05	0.82	0.01	wgt
NPA-OFF	NPA-TAC-ON	Age	66.2	66.63	9.53	0.05	0.56	0.04	wgt
NPA-OFF	NPA-TAC-ON	Gender(Male)	0.82	0.81	0.4	0	0.97	0	wgt
NPA-OFF	NPA-TAC-ON	Hypertension	0.63	0.53	0.49	0.12	0.01	0.1	wgt
NPA-OFF	NPA-TAC-ON	Smoker	0.38	0.34	0.48	0.09	0.27	0.04	wgt
NPA-OFF	NPA-TAC-ON	Hypercholesterolemia	0.45	0.42	0.5	0.06	0.5	0.03	wgt
NPA-OFF	NPA-TAC-ON	Diabetes	0.3	0.22	0.43	0.14	0.03	0.08	wgt
NPA-OFF	NPA-TAC-ON	Insulin Dependent Diabetes	0.08	0.08	0.29	0.01	0.88	0	wgt
NPA-OFF	NPA-TAC-ON	COPD	0.12	0.07	0.25	0.15	0.06	0.05	wgt
NPA-OFF	NPA-TAC-ON	AKD	0.06	0.03	0.2	0.14	0.08	0.03	wgt
NPA-OFF	NPA-TAC-ON	Dialysis	0.01	0.01	0.09	0.04	0.51	0	wgt
NPA-OFF	NPA-TAC-ON	Stroke History	0.04	0.02	0.17	0.08	0.34	0.01	wgt
NPA-OFF	NPA-TAC-ON	TIA History	0.03	0.01	0.17	0.08	0.23	0.01	wgt
NPA-OFF	NPA-TAC-ON	Carotid Stenosis > 50%	0.14	0.07	0.27	0.15	0	0.08	wgt
NPA-OFF	NPA-TAC-ON	PAD	0.18	0.16	0.38	0.06	0.49	0.02	wgt

NPA-OFF	NPA-TAC-ON	Pulmonary Hypertension	0.01	0.04	0.21	0.11	0.04	0.02	wgt
NPA-OFF	NPA-TAC-ON	IABP Preop	0.01	0.01	0.12	0.03	0.6	0	wgt
NPA-OFF	NPA-TAC-ON	LVEF	52.84	53.22	11.36	0.03	0.62	0.03	wgt
NPA-OFF	NPA-TAC-ON	LMCA Stenosis	0.27	0.33	0.47	0.13	0.11	0.06	wgt
NPA-OFF	NPA-TAC-ON	Two Vessels Disease	0.29	0.36	0.47	0.16	0.02	0.08	wgt
NPA-OFF	NPA-TAC-ON	Previuos PTCA	0.27	0.26	0.46	0.01	0.92	0	wgt
NPA-OFF	NPA-TAC-ON	Antiplatelet Drugs	0.26	0.3	0.47	0.1	0.22	0.04	wgt
NPA-OFF	NPA-TAC-ON	Surgery	0.05	0.07	0.28	0.06	0.36	0.02	wgt
NPA-OFF	NPA-TAC-ON	REDO	0.04	0.05	0.2	0.03	0.72	0.01	wgt
NPA-OFF	NPA-TAC-ON	NYHA ≥ 3	0.15	0.1	0.36	0.14	0.07	0.05	wgt
NPA-OFF	NPA-TAC-ON	≥ 3-Vessel Disease	0.59	0.58	0.49	0.03	0.65	0.02	wgt
NPA-OFF	NPA-TAC-ON	LAD Stenosis	0.91	0.94	0.24	0.09	0.22	0.02	wgt
NPA-OFF	SINGLE TAC-ON	Age	66.2	66.73	9.53	0.06	0.39	0.05	wgt
NPA-OFF	SINGLE TAC-ON	Gender(Male)	0.82	0.79	0.4	0.05	0.41	0.02	wgt
NPA-OFF	SINGLE TAC-ON	Hypertension	0.63	0.6	0.49	0.07	0.25	0.03	wgt
NPA-OFF	SINGLE TAC-ON	Smoker	0.38	0.37	0.48	0.03	0.64	0.01	wgt
NPA-OFF	SINGLE TAC-ON	Hypercholesterolemia	0.45	0.46	0.5	0.02	0.77	0.01	wgt
NPA-OFF	SINGLE TAC-ON	Diabetes	0.3	0.26	0.43	0.09	0.17	0.04	wgt
NPA-OFF	SINGLE TAC-ON	Insulin Dependent Diabetes	0.08	0.09	0.29	0.05	0.4	0.01	wgt
NPA-OFF	SINGLE TAC-ON	COPD	0.12	0.07	0.25	0.17	0.05	0.04	wgt
NPA-OFF	SINGLE TAC-ON	AKD	0.06	0.04	0.2	0.06	0.37	0.01	wgt
NPA-OFF	SINGLE TAC-ON	Dialysis	0.01	0.01	0.09	0.02	0.69	0	wgt
NPA-OFF	SINGLE TAC-ON	Stroke History	0.04	0.03	0.17	0.04	0.61	0.01	wgt
NPA-OFF	SINGLE TAC-ON	TIA History	0.03	0.03	0.17	0	0.95	0	wgt
NPA-OFF	SINGLE TAC-ON	Carotid Stenosis > 50%	0.14	0.08	0.27	0.08	0.01	0.06	wgt
NPA-OFF	SINGLE TAC-ON	PAD	0.18	0.18	0.38	0.02	0.8	0.01	wgt
NPA-OFF	SINGLE TAC-ON	Pulmonary Hypertension	0.01	0.05	0.21	0.16	0	0.03	wgt
NPA-OFF	SINGLE TAC-ON	IABP Preop	0.01	0.01	0.12	0.03	0.52	0	wgt
NPA-OFF	SINGLE TAC-ON	LVEF	52.84	52.42	11.36	0.04	0.5	0.05	wgt
NPA-OFF	SINGLE TAC-ON	LMCA Stenosis	0.27	0.33	0.47	0.14	0.02	0.06	wgt
NPA-OFF	SINGLE TAC-ON	Two Vessels Disease	0.29	0.33	0.47	0.1	0.05	0.05	wgt
NPA-OFF	SINGLE TAC-ON	Previuos PTCA	0.27	0.3	0.46	0.06	0.37	0.03	wgt

NPA-OFF	SINGLE TAC-ON	Antiplatelet Drugs	0.26	0.33	0.47	0.15	0.02	0.07	wgt
NPA-OFF	SINGLE TAC-ON	Surgery	0.05	0.08	0.28	0.1	0.07	0.03	wgt
NPA-OFF	SINGLE TAC-ON	REDO	0.04	0.05	0.2	0.01	0.89	0	wgt
NPA-OFF	SINGLE TAC-ON	NYHA ≥ 3	0.15	0.13	0.36	0.05	0.4	0.02	wgt
NPA-OFF	SINGLE TAC-ON	≥ 3-Vessel Disease	0.59	0.62	0.49	0.04	0.47	0.02	wgt
NPA-OFF	SINGLE TAC-ON	LAD Stenosis	0.91	0.94	0.24	0.1	0.03	0.03	wgt
NPA-OFF	TAC-SBCL-ON	Age	66.2	66.89	9.53	0.07	0.25	0.05	wgt
NPA-OFF	TAC-SBCL-ON	Gender(Male)	0.82	0.8	0.4	0.03	0.61	0.01	wgt
NPA-OFF	TAC-SBCL-ON	Hypertension	0.63	0.56	0.49	0.15	0.01	0.08	wgt
NPA-OFF	TAC-SBCL-ON	Smoker	0.38	0.33	0.48	0.11	0.09	0.05	wgt
NPA-OFF	TAC-SBCL-ON	Hypercholesterolemia	0.45	0.4	0.5	0.09	0.16	0.04	wgt
NPA-OFF	TAC-SBCL-ON	Diabetes	0.3	0.25	0.43	0.12	0.07	0.05	wgt
NPA-OFF	TAC-SBCL-ON	Insulin Dependent Diabetes	0.08	0.08	0.29	0.02	0.78	0.01	wgt
NPA-OFF	TAC-SBCL-ON	COPD	0.12	0.07	0.25	0.1	0.03	0.05	wgt
NPA-OFF	TAC-SBCL-ON	AKD	0.06	0.04	0.2	0.08	0.2	0.02	wgt
NPA-OFF	TAC-SBCL-ON	Dialysis	0.01	0.01	0.09	0.02	0.67	0	wgt
NPA-OFF	TAC-SBCL-ON	Stroke History	0.04	0.02	0.17	0.1	0.17	0.02	wgt
NPA-OFF	TAC-SBCL-ON	TIA History	0.03	0.03	0.17	0	0.97	0	wgt
NPA-OFF	TAC-SBCL-ON	Carotid Stenosis > 50%	0.14	0.08	0.27	0.13	0.01	0.06	wgt
NPA-OFF	TAC-SBCL-ON	PAD	0.18	0.17	0.38	0.03	0.65	0.01	wgt
NPA-OFF	TAC-SBCL-ON	Pulmonary Hypertension	0.01	0.05	0.21	0.14	0	0.04	wgt
NPA-OFF	TAC-SBCL-ON	IABP Preop	0.01	0.02	0.12	0.05	0.25	0.01	wgt
NPA-OFF	TAC-SBCL-ON	LVEF	52.84	52.91	11.36	0.01	0.9	0.04	wgt
NPA-OFF	TAC-SBCL-ON	LMCA Stenosis	0.27	0.33	0.47	0.14	0.02	0.07	wgt
NPA-OFF	TAC-SBCL-ON	Two Vessels Disease	0.29	0.34	0.47	0.12	0.01	0.06	wgt
NPA-OFF	TAC-SBCL-ON	Previuos PTCA	0.27	0.33	0.46	0.14	0.04	0.06	wgt
NPA-OFF	TAC-SBCL-ON	Antiplatelet Drugs	0.26	0.36	0.47	0.16	0	0.1	wgt
NPA-OFF	TAC-SBCL-ON	Surgery	0.05	0.09	0.28	0.14	0.02	0.04	wgt
NPA-OFF	TAC-SBCL-ON	REDO	0.04	0.04	0.2	0.01	0.93	0	wgt
NPA-OFF	TAC-SBCL-ON	NYHA ≥ 3	0.15	0.13	0.36	0.06	0.36	0.02	wgt
NPA-OFF	TAC-SBCL-ON	≥ 3-Vessel Disease	0.59	0.63	0.49	0.08	0.16	0.04	wgt
NPA-OFF	TAC-SBCL-ON	LAD Stenosis	0.91	0.95	0.24	0.16	0	0.04	wgt

SBCL-OFF	NPA-BHON	Age	67.21	66.94	9.53	0.03	0.76	0.03	wgt
SBCL-OFF	NPA-BHON	Gender(Male)	0.8	0.75	0.4	0.12	0.28	0.05	wgt
SBCL-OFF	NPA-BHON	Hypertension	0.62	0.58	0.49	0.08	0.38	0.04	wgt
SBCL-OFF	NPA-BHON	Smoker	0.36	0.3	0.48	0.12	0.25	0.06	wgt
SBCL-OFF	NPA-BHON	Hypercholesterolemia	0.43	0.46	0.5	0.06	0.59	0.03	wgt
SBCL-OFF	NPA-BHON	Diabetes	0.25	0.26	0.43	0.02	0.87	0.01	wgt
SBCL-OFF	NPA-BHON	Insulin Dependent Diabetes	0.08	0.07	0.29	0.02	0.83	0.01	wgt
SBCL-OFF	NPA-BHON	COPD	0.07	0.04	0.25	0.09	0.08	0.02	wgt
SBCL-OFF	NPA-BHON	AKD	0.04	0.02	0.2	0.13	0.02	0.03	wgt
SBCL-OFF	NPA-BHON	Dialysis	0	0	0.09	0.02	0.58	0	wgt
SBCL-OFF	NPA-BHON	Stroke History	0.02	0.03	0.17	0.03	0.68	0.01	wgt
SBCL-OFF	NPA-BHON	TIA History	0.02	0.01	0.17	0.09	0.04	0.01	wgt
SBCL-OFF	NPA-BHON	Carotid Stenosis > 50%	0.09	0.04	0.27	0.16	0.02	0.04	wgt
SBCL-OFF	NPA-BHON	PAD	0.17	0.12	0.38	0.13	0.23	0.05	wgt
SBCL-OFF	NPA-BHON	Pulmonary Hypertension	0.05	0.05	0.21	0.02	0.84	0	wgt
SBCL-OFF	NPA-BHON	IABP Preop	0.02	0.01	0.12	0.08	0.4	0.01	wgt
SBCL-OFF	NPA-BHON	LVEF	53.05	53.79	11.36	0.06	0.41	0.06	wgt
SBCL-OFF	NPA-BHON	LMCA Stenosis	0.31	0.25	0.47	0.13	0.15	0.06	wgt
SBCL-OFF	NPA-BHON	Two Vessels Disease	0.39	0.37	0.47	0.04	0.63	0.02	wgt
SBCL-OFF	NPA-BHON	Previuos PTCA	0.27	0.32	0.46	0.1	0.39	0.05	wgt
SBCL-OFF	NPA-BHON	Antiplatelet Drugs	0.3	0.36	0.47	0.11	0.29	0.05	wgt
SBCL-OFF	NPA-BHON	Surgery	0.07	0.05	0.28	0.06	0.43	0.02	wgt
SBCL-OFF	NPA-BHON	REDO	0.04	0.05	0.2	0.04	0.67	0.01	wgt
SBCL-OFF	NPA-BHON	NYHA ≥ 3	0.16	0.09	0.36	0.15	0.04	0.08	wgt
SBCL-OFF	NPA-BHON	≥ 3-Vessel Disease	0.59	0.51	0.49	0.16	0.11	0.08	wgt
SBCL-OFF	NPA-BHON	LAD Stenosis	0.97	0.97	0.24	0.03	0.49	0.01	wgt
SBCL-OFF	SBCL-BHON	Age	67.21	67.48	9.53	0.03	0.74	0.03	wgt
SBCL-OFF	SBCL-BHON	Gender(Male)	0.8	0.84	0.4	0.11	0.07	0.04	wgt
SBCL-OFF	SBCL-BHON	Hypertension	0.62	0.65	0.49	0.06	0.54	0.03	wgt
SBCL-OFF	SBCL-BHON	Smoker	0.36	0.4	0.48	0.08	0.29	0.04	wgt
SBCL-OFF	SBCL-BHON	Hypercholesterolemia	0.43	0.5	0.5	0.15	0.07	0.07	wgt
SBCL-OFF	SBCL-BHON	Diabetes	0.25	0.25	0.43	0	0.99	0	wgt

SBCL-OFF	SBCL-BHON	Insulin Dependent Diabetes	0.08	0.1	0.29	0.09	0.13	0.03	wgt
SBCL-OFF	SBCL-BHON	COPD	0.07	0.06	0.25	0.04	0.63	0.01	wgt
SBCL-OFF	SBCL-BHON	AKD	0.04	0.03	0.2	0.08	0.4	0.02	wgt
SBCL-OFF	SBCL-BHON	Dialysis	0	0	0.09	0	0.95	0	wgt
SBCL-OFF	SBCL-BHON	Stroke History	0.02	0.01	0.17	0.07	0.2	0.01	wgt
SBCL-OFF	SBCL-BHON	TIA History	0.02	0.01	0.17	0.08	0.06	0.01	wgt
SBCL-OFF	SBCL-BHON	Carotid Stenosis > 50%	0.09	0.03	0.27	0.11	0	0.06	wgt
SBCL-OFF	SBCL-BHON	PAD	0.17	0.17	0.38	0.02	0.79	0.01	wgt
SBCL-OFF	SBCL-BHON	Pulmonary Hypertension	0.05	0.04	0.21	0.09	0.33	0.02	wgt
SBCL-OFF	SBCL-BHON	IABP Preop	0.02	0.01	0.12	0.08	0.12	0.01	wgt
SBCL-OFF	SBCL-BHON	LVEF	53.05	52.71	11.36	0.03	0.67	0.08	wgt
SBCL-OFF	SBCL-BHON	LMCA Stenosis	0.31	0.37	0.47	0.11	0.24	0.05	wgt
SBCL-OFF	SBCL-BHON	Two Vessels Disease	0.39	0.33	0.47	0.13	0.08	0.06	wgt
SBCL-OFF	SBCL-BHON	Previuos PTCA	0.27	0.28	0.46	0.01	0.89	0	wgt
SBCL-OFF	SBCL-BHON	Antiplatelet Drugs	0.3	0.35	0.47	0.09	0.23	0.04	wgt
SBCL-OFF	SBCL-BHON	Surgery	0.07	0.06	0.28	0.06	0.3	0.02	wgt
SBCL-OFF	SBCL-BHON	REDO	0.04	0	0.2	0.12	0	0.04	wgt
SBCL-OFF	SBCL-BHON	NYHA ≥ 3	0.16	0.16	0.36	0	0.97	0	wgt
SBCL-OFF	SBCL-BHON	≥ 3-Vessel Disease	0.59	0.59	0.49	0	0.97	0	wgt
SBCL-OFF	SBCL-BHON	LAD Stenosis	0.97	0.93	0.24	0.09	0.38	0.05	wgt
SBCL-OFF	NPA-TAC-ON	Age	67.21	66.63	9.53	0.06	0.32	0.04	wgt
SBCL-OFF	NPA-TAC-ON	Gender(Male)	0.8	0.81	0.4	0.03	0.58	0.01	wgt
SBCL-OFF	NPA-TAC-ON	Hypertension	0.62	0.53	0.49	0.15	0	0.09	wgt
SBCL-OFF	NPA-TAC-ON	Smoker	0.36	0.34	0.48	0.04	0.55	0.02	wgt
SBCL-OFF	NPA-TAC-ON	Hypercholesterolemia	0.43	0.42	0.5	0.02	0.81	0.01	wgt
SBCL-OFF	NPA-TAC-ON	Diabetes	0.25	0.22	0.43	0.07	0.24	0.03	wgt
SBCL-OFF	NPA-TAC-ON	Insulin Dependent Diabetes	0.08	0.08	0.29	0	0.97	0	wgt
SBCL-OFF	NPA-TAC-ON	COPD	0.07	0.07	0.25	0	0.99	0	wgt
SBCL-OFF	NPA-TAC-ON	AKD	0.04	0.03	0.2	0.07	0.23	0.02	wgt
SBCL-OFF	NPA-TAC-ON	Dialysis	0	0.01	0.09	0.02	0.71	0	wgt
SBCL-OFF	NPA-TAC-ON	Stroke History	0.02	0.02	0.17	0.01	0.81	0	wgt
SBCL-OFF	NPA-TAC-ON	TIA History	0.02	0.01	0.17	0.06	0.24	0.01	wgt

SBCL-OFF	NPA-TAC-ON	Carotid Stenosis > 50%	0.09	0.07	0.27	0.08	0.22	0.02	wgt
SBCL-OFF	NPA-TAC-ON	PAD	0.17	0.16	0.38	0.03	0.67	0.01	wgt
SBCL-OFF	NPA-TAC-ON	Pulmonary Hypertension	0.05	0.04	0.21	0.08	0.19	0.02	wgt
SBCL-OFF	NPA-TAC-ON	IABP Preop	0.02	0.01	0.12	0.12	0.06	0.01	wgt
SBCL-OFF	NPA-TAC-ON	LVEF	53.05	53.22	11.36	0.01	0.81	0.04	wgt
SBCL-OFF	NPA-TAC-ON	LMCA Stenosis	0.31	0.33	0.47	0.03	0.68	0.01	wgt
SBCL-OFF	NPA-TAC-ON	Two Vessels Disease	0.39	0.36	0.47	0.05	0.38	0.03	wgt
SBCL-OFF	NPA-TAC-ON	Previuos PTCA	0.27	0.26	0.46	0.02	0.82	0.01	wgt
SBCL-OFF	NPA-TAC-ON	Antiplatelet Drugs	0.3	0.3	0.47	0	0.98	0	wgt
SBCL-OFF	NPA-TAC-ON	Surgery	0.07	0.07	0.28	0.01	0.89	0	wgt
SBCL-OFF	NPA-TAC-ON	REDO	0.04	0.05	0.2	0.04	0.58	0.01	wgt
SBCL-OFF	NPA-TAC-ON	NYHA ≥ 3	0.16	0.1	0.36	0.17	0.01	0.06	wgt
SBCL-OFF	NPA-TAC-ON	≥ 3-Vessel Disease	0.59	0.58	0.49	0.02	0.72	0.01	wgt
SBCL-OFF	NPA-TAC-ON	LAD Stenosis	0.97	0.94	0.24	0.15	0.02	0.04	wgt
SBCL-OFF	SINGLE TAC-ON	Age	67.21	66.73	9.53	0.05	0.24	0.04	wgt
SBCL-OFF	SINGLE TAC-ON	Gender(Male)	0.8	0.79	0.4	0.02	0.72	0.01	wgt
SBCL-OFF	SINGLE TAC-ON	Hypertension	0.62	0.6	0.49	0.05	0.24	0.02	wgt
SBCL-OFF	SINGLE TAC-ON	rr	0.36	0.37	0.48	0.02	0.66	0.01	wgt
SBCL-OFF	SINGLE TAC-ON	Hypercholesterolemia	0.43	0.46	0.5	0.06	0.19	0.03	wgt
SBCL-OFF	SINGLE TAC-ON	Diabetes	0.25	0.26	0.43	0.01	0.82	0	wgt
SBCL-OFF	SINGLE TAC-ON	Insulin Dependent Diabetes	0.08	0.09	0.29	0.06	0.18	0.02	wgt
SBCL-OFF	SINGLE TAC-ON	COPD	0.07	0.07	0.25	0.02	0.66	0	wgt
SBCL-OFF	SINGLE TAC-ON	AKD	0.04	0.04	0.2	0	0.99	0	wgt
SBCL-OFF	SINGLE TAC-ON	Dialysis	0	0.01	0.09	0.03	0.33	0	wgt
SBCL-OFF	SINGLE TAC-ON	Stroke History	0.02	0.03	0.17	0.06	0.17	0.01	wgt
SBCL-OFF	SINGLE TAC-ON	TIA History	0.02	0.03	0.17	0.03	0.47	0	wgt
SBCL-OFF	SINGLE TAC-ON	Carotid Stenosis > 50%	0.09	0.08	0.27	0.01	0.78	0	wgt
SBCL-OFF	SINGLE TAC-ON	PAD	0.17	0.18	0.38	0.04	0.36	0.02	wgt
SBCL-OFF	SINGLE TAC-ON	Pulmonary Hypertension	0.05	0.05	0.21	0.03	0.58	0.01	wgt
SBCL-OFF	SINGLE TAC-ON	IABP Preop	0.02	0.01	0.12	0.12	0.03	0.01	wgt
SBCL-OFF	SINGLE TAC-ON	LVEF	53.05	52.42	11.36	0.06	0.2	0.05	wgt
SBCL-OFF	SINGLE TAC-ON	LMCA Stenosis	0.31	0.33	0.47	0.04	0.41	0.02	wgt

SBCL-OFF	SINGLE TAC-ON	Two Vessels Disease	0.39	0.33	0.47	0.11	0.02	0.05	wgt
SBCL-OFF	SINGLE TAC-ON	Previuos PTCA	0.27	0.3	0.46	0.06	0.23	0.03	wgt
SBCL-OFF	SINGLE TAC-ON	Antiplatelet Drugs	0.3	0.33	0.47	0.05	0.22	0.02	wgt
SBCL-OFF	SINGLE TAC-ON	Surgery	0.07	0.08	0.28	0.03	0.46	0.01	wgt
SBCL-OFF	SINGLE TAC-ON	REDO	0.04	0.05	0.2	0.02	0.67	0	wgt
SBCL-OFF	SINGLE TAC-ON	NYHA ≥ 3	0.16	0.13	0.36	0.09	0.09	0.03	wgt
SBCL-OFF	SINGLE TAC-ON	≥ 3-Vessel Disease	0.59	0.62	0.49	0.05	0.25	0.03	wgt
SBCL-OFF	SINGLE TAC-ON	LAD Stenosis	0.97	0.94	0.24	0.14	0	0.03	wgt
SBCL-OFF	TAC-SBCL-ON	Age	67.21	66.89	9.53	0.03	0.42	0.03	wgt
SBCL-OFF	TAC-SBCL-ON	Gender(Male)	0.8	0.8	0.4	0	0.92	0	wgt
SBCL-OFF	TAC-SBCL-ON	Hypertension	0.62	0.56	0.49	0.13	0	0.07	wgt
SBCL-OFF	TAC-SBCL-ON	Smoker	0.36	0.33	0.48	0.06	0.2	0.03	wgt
SBCL-OFF	TAC-SBCL-ON	Hypercholesterolemia	0.43	0.4	0.5	0.05	0.26	0.02	wgt
SBCL-OFF	TAC-SBCL-ON	Diabetes	0.25	0.25	0.43	0.01	0.71	0.01	wgt
SBCL-OFF	TAC-SBCL-ON	Insulin Dependent Diabetes	0.08	0.08	0.29	0.03	0.56	0.01	wgt
SBCL-OFF	TAC-SBCL-ON	COPD	0.07	0.07	0.25	0	1	0	wgt
SBCL-OFF	TAC-SBCL-ON	AKD	0.04	0.04	0.2	0.02	0.63	0	wgt
SBCL-OFF	TAC-SBCL-ON	Dialysis	0	0.01	0.09	0.03	0.3	0	wgt
SBCL-OFF	TAC-SBCL-ON	Stroke History	0.02	0.02	0.17	0	0.97	0	wgt
SBCL-OFF	TAC-SBCL-ON	TIA History	0.02	0.03	0.17	0.03	0.49	0	wgt
SBCL-OFF	TAC-SBCL-ON	Carotid Stenosis > 50%	0.09	0.08	0.27	0.03	0.64	0.01	wgt
SBCL-OFF	TAC-SBCL-ON	PAD	0.17	0.17	0.38	0	0.96	0	wgt
SBCL-OFF	TAC-SBCL-ON	Pulmonary Hypertension	0.05	0.05	0.21	0.01	0.9	0	wgt
SBCL-OFF	TAC-SBCL-ON	IABP Preop	0.02	0.02	0.12	0.03	0.53	0	wgt
SBCL-OFF	TAC-SBCL-ON	LVEF	53.05	52.91	11.36	0.01	0.76	0.02	wgt
SBCL-OFF	TAC-SBCL-ON	LMCA Stenosis	0.31	0.33	0.47	0.04	0.36	0.02	wgt
SBCL-OFF	TAC-SBCL-ON	Two Vessels Disease	0.39	0.34	0.47	0.09	0.03	0.04	wgt
SBCL-OFF	TAC-SBCL-ON	Previuos PTCA	0.27	0.33	0.46	0.13	0	0.06	wgt
SBCL-OFF	TAC-SBCL-ON	Antiplatelet Drugs	0.3	0.36	0.47	0.12	0.01	0.06	wgt
SBCL-OFF	TAC-SBCL-ON	Surgery	0.07	0.09	0.28	0.07	0.16	0.02	wgt
SBCL-OFF	TAC-SBCL-ON	REDO	0.04	0.04	0.2	0.01	0.89	0	wgt
SBCL-OFF	TAC-SBCL-ON	NYHA ≥ 3	0.16	0.13	0.36	0.09	0.05	0.03	wgt

SBCL-OFF	TAC-SBCL-ON	≥ 3-Vessel Disease	0.59	0.63	0.49	0.09	0.03	0.04	wgt
SBCL-OFF	TAC-SBCL-ON	LAD Stenosis	0.97	0.95	0.24	0.09	0.01	0.02	wgt
NPA-BHON	SBCL-BHON	Age	66.94	67.48	9.53	0.06	0.63	0.06	wgt
NPA-BHON	SBCL-BHON	Gender(Male)	0.75	0.84	0.4	0.14	0.05	0.09	wgt
NPA-BHON	SBCL-BHON	Hypertension	0.58	0.65	0.49	0.14	0.27	0.07	wgt
NPA-BHON	SBCL-BHON	Smoker	0.3	0.4	0.48	0.13	0.09	0.1	wgt
NPA-BHON	SBCL-BHON	Hypercholesterolemia	0.46	0.5	0.5	0.09	0.44	0.05	wgt
NPA-BHON	SBCL-BHON	Diabetes	0.26	0.25	0.43	0.02	0.88	0.01	wgt
NPA-BHON	SBCL-BHON	Insulin Dependent Diabetes	0.07	0.1	0.29	0.11	0.27	0.03	wgt
NPA-BHON	SBCL-BHON	COPD	0.04	0.06	0.25	0.05	0.55	0.01	wgt
NPA-BHON	SBCL-BHON	AKD	0.02	0.03	0.2	0.05	0.56	0.01	wgt
NPA-BHON	SBCL-BHON	Dialysis	0	0	0.09	0.02	0.41	0	wgt
NPA-BHON	SBCL-BHON	Stroke History	0.03	0.01	0.17	0.11	0.19	0.02	wgt
NPA-BHON	SBCL-BHON	TIA History	0.01	0.01	0.17	0.01	0.75	0	wgt
NPA-BHON	SBCL-BHON	Carotid Stenosis > 50%	0.04	0.03	0.27	0.07	0.22	0.02	wgt
NPA-BHON	SBCL-BHON	PAD	0.12	0.17	0.38	0.15	0.19	0.05	wgt
NPA-BHON	SBCL-BHON	Pulmonary Hypertension	0.05	0.04	0.21	0.07	0.55	0.01	wgt
NPA-BHON	SBCL-BHON	IABP Preop	0.01	0.01	0.12	0	0.98	0	wgt
NPA-BHON	SBCL-BHON	LVEF	53.79	52.71	11.36	0.1	0.3	0.06	wgt
NPA-BHON	SBCL-BHON	LMCA Stenosis	0.25	0.37	0.47	0.11	0.05	0.12	wgt
NPA-BHON	SBCL-BHON	Two Vessels Disease	0.37	0.33	0.47	0.09	0.41	0.04	wgt
NPA-BHON	SBCL-BHON	Previuos PTCA	0.32	0.28	0.46	0.09	0.48	0.04	wgt
NPA-BHON	SBCL-BHON	Antiplatelet Drugs	0.36	0.35	0.47	0.02	0.87	0.01	wgt
NPA-BHON	SBCL-BHON	Surgery	0.05	0.06	0.28	0.01	0.95	0	wgt
NPA-BHON	SBCL-BHON	REDO	0.05	0	0.2	0.12	0.01	0.04	wgt
NPA-BHON	SBCL-BHON	NYHA ≥ 3	0.09	0.16	0.36	0.11	0.05	0.08	wgt
NPA-BHON	SBCL-BHON	≥ 3-Vessel Disease	0.51	0.59	0.49	0.16	0.21	0.08	wgt
NPA-BHON	SBCL-BHON	LAD Stenosis	0.97	0.93	0.24	0.16	0.47	0.04	wgt
NPA-BHON	NPA-TAC-ON	Age	66.94	66.63	9.53	0.03	0.75	0.07	wgt
NPA-BHON	NPA-TAC-ON	Gender(Male)	0.75	0.81	0.4	0.16	0.19	0.06	wgt
NPA-BHON	NPA-TAC-ON	Hypertension	0.58	0.53	0.49	0.11	0.27	0.05	wgt
NPA-BHON	NPA-TAC-ON	Smoker	0.3	0.34	0.48	0.08	0.49	0.04	wgt

NPA-BHON	NPA-TAC-ON	Hypercholesterolemia	0.46	0.42	0.5	0.07	0.53	0.04	wgt
NPA-BHON	NPA-TAC-ON	Diabetes	0.26	0.22	0.43	0.09	0.4	0.04	wgt
NPA-BHON	NPA-TAC-ON	Insulin Dependent Diabetes	0.07	0.08	0.29	0.02	0.82	0.01	wgt
NPA-BHON	NPA-TAC-ON	COPD	0.04	0.07	0.25	0.09	0.17	0.02	wgt
NPA-BHON	NPA-TAC-ON	AKD	0.02	0.03	0.2	0.05	0.33	0.01	wgt
NPA-BHON	NPA-TAC-ON	Dialysis	0	0.01	0.09	0.03	0.34	0	wgt
NPA-BHON	NPA-TAC-ON	Stroke History	0.03	0.02	0.17	0.02	0.84	0	wgt
NPA-BHON	NPA-TAC-ON	TIA History	0.01	0.01	0.17	0.03	0.47	0.01	wgt
NPA-BHON	NPA-TAC-ON	Carotid Stenosis > 50%	0.04	0.07	0.27	0.08	0.29	0.02	wgt
NPA-BHON	NPA-TAC-ON	PAD	0.12	0.16	0.38	0.1	0.38	0.04	wgt
NPA-BHON	NPA-TAC-ON	Pulmonary Hypertension	0.05	0.04	0.21	0.06	0.5	0.01	wgt
NPA-BHON	NPA-TAC-ON	IABP Preop	0.01	0.01	0.12	0.03	0.71	0	wgt
NPA-BHON	NPA-TAC-ON	LVEF	53.79	53.22	11.36	0.05	0.55	0.06	wgt
NPA-BHON	NPA-TAC-ON	LMCA Stenosis	0.25	0.33	0.47	0.16	0.12	0.08	wgt
NPA-BHON	NPA-TAC-ON	Two Vessels Disease	0.37	0.36	0.47	0.01	0.9	0.01	wgt
NPA-BHON	NPA-TAC-ON	Previuos PTCA	0.32	0.26	0.46	0.12	0.36	0.05	wgt
NPA-BHON	NPA-TAC-ON	Antiplatelet Drugs	0.36	0.3	0.47	0.11	0.34	0.05	wgt
NPA-BHON	NPA-TAC-ON	Surgery	0.05	0.07	0.28	0.06	0.47	0.02	wgt
NPA-BHON	NPA-TAC-ON	REDO	0.05	0.05	0.2	0	0.98	0	wgt
NPA-BHON	NPA-TAC-ON	NYHA ≥ 3	0.09	0.1	0.36	0.04	0.72	0.01	wgt
NPA-BHON	NPA-TAC-ON	≥ 3-Vessel Disease	0.51	0.58	0.49	0.14	0.2	0.07	wgt
NPA-BHON	NPA-TAC-ON	LAD Stenosis	0.97	0.94	0.24	0.12	0.09	0.03	wgt
NPA-BHON	SINGLE TAC-ON	Age	66.94	66.73	9.53	0.02	0.81	0.07	wgt
NPA-BHON	SINGLE TAC-ON	Gender(Male)	0.75	0.79	0.4	0.11	0.33	0.04	wgt
NPA-BHON	SINGLE TAC-ON	Hypertension	0.58	0.6	0.49	0.03	0.73	0.01	wgt
NPA-BHON	SINGLE TAC-ON	Smoker	0.3	0.37	0.48	0.14	0.16	0.07	wgt
NPA-BHON	SINGLE TAC-ON	Hypercholesterolemia	0.46	0.46	0.5	0	0.97	0	wgt
NPA-BHON	SINGLE TAC-ON	Diabetes	0.26	0.26	0.43	0.01	0.95	0	wgt
NPA-BHON	SINGLE TAC-ON	Insulin Dependent Diabetes	0.07	0.09	0.29	0.09	0.36	0.02	wgt
NPA-BHON	SINGLE TAC-ON	COPD	0.04	0.07	0.25	0.11	0.01	0.03	wgt
NPA-BHON	SINGLE TAC-ON	AKD	0.02	0.04	0.2	0.13	0	0.03	wgt
NPA-BHON	SINGLE TAC-ON	Dialysis	0	0.01	0.09	0.05	0.03	0	wgt

NPA-BHON	SINGLE TAC-ON	Stroke History	0.03	0.03	0.17	0.03	0.7	0	wgt
NPA-BHON	SINGLE TAC-ON	TIA History	0.01	0.03	0.17	0.11	0	0.02	wgt
NPA-BHON	SINGLE TAC-ON	Carotid Stenosis > 50%	0.04	0.08	0.27	0.15	0.01	0.04	wgt
NPA-BHON	SINGLE TAC-ON	PAD	0.12	0.18	0.38	0.17	0.08	0.06	wgt
NPA-BHON	SINGLE TAC-ON	Pulmonary Hypertension	0.05	0.05	0.21	0.01	0.94	0	wgt
NPA-BHON	SINGLE TAC-ON	IABP Preop	0.01	0.01	0.12	0.03	0.69	0	wgt
NPA-BHON	SINGLE TAC-ON	LVEF	53.79	52.42	11.36	0.12	0.1	0.1	wgt
NPA-BHON	SINGLE TAC-ON	LMCA Stenosis	0.25	0.33	0.47	0.17	0.06	0.08	wgt
NPA-BHON	SINGLE TAC-ON	Two Vessels Disease	0.37	0.33	0.47	0.07	0.44	0.03	wgt
NPA-BHON	SINGLE TAC-ON	Previuos PTCA	0.32	0.3	0.46	0.04	0.7	0.02	wgt
NPA-BHON	SINGLE TAC-ON	Antiplatelet Drugs	0.36	0.33	0.47	0.06	0.57	0.03	wgt
NPA-BHON	SINGLE TAC-ON	Surgery	0.05	0.08	0.28	0.1	0.17	0.03	wgt
NPA-BHON	SINGLE TAC-ON	REDO	0.05	0.05	0.2	0.02	0.82	0	wgt
NPA-BHON	SINGLE TAC-ON	NYHA ≥ 3	0.09	0.13	0.36	0.12	0.2	0.04	wgt
NPA-BHON	SINGLE TAC-ON	≥ 3-Vessel Disease	0.51	0.62	0.49	0.11	0.03	0.1	wgt
NPA-BHON	SINGLE TAC-ON	LAD Stenosis	0.97	0.94	0.24	0.11	0.01	0.03	wgt
NPA-BHON	TAC-SBCL-ON	Age	66.94	66.89	9.53	0	0.96	0.04	wgt
NPA-BHON	TAC-SBCL-ON	Gender(Male)	0.75	0.8	0.4	0.13	0.25	0.05	wgt
NPA-BHON	TAC-SBCL-ON	Hypertension	0.58	0.56	0.49	0.06	0.52	0.03	wgt
NPA-BHON	TAC-SBCL-ON	Smoker	0.3	0.33	0.48	0.06	0.54	0.03	wgt
NPA-BHON	TAC-SBCL-ON	Hypercholesterolemia	0.46	0.4	0.5	0.1	0.29	0.05	wgt
NPA-BHON	TAC-SBCL-ON	Diabetes	0.26	0.25	0.43	0.03	0.75	0.01	wgt
NPA-BHON	TAC-SBCL-ON	Insulin Dependent Diabetes	0.07	0.08	0.29	0.05	0.58	0.02	wgt
NPA-BHON	TAC-SBCL-ON	COPD	0.04	0.07	0.25	0.09	0.03	0.02	wgt
NPA-BHON	TAC-SBCL-ON	AKD	0.02	0.04	0.2	0.1	0	0.02	wgt
NPA-BHON	TAC-SBCL-ON	Dialysis	0	0.01	0.09	0.05	0.01	0	wgt
NPA-BHON	TAC-SBCL-ON	Stroke History	0.03	0.02	0.17	0.03	0.63	0.01	wgt
NPA-BHON	TAC-SBCL-ON	TIA History	0.01	0.03	0.17	0.11	0	0.02	wgt
NPA-BHON	TAC-SBCL-ON	Carotid Stenosis > 50%	0.04	0.08	0.27	0.13	0.05	0.04	wgt
NPA-BHON	TAC-SBCL-ON	PAD	0.12	0.17	0.38	0.12	0.22	0.05	wgt
NPA-BHON	TAC-SBCL-ON	Pulmonary Hypertension	0.05	0.05	0.21	0.01	0.91	0	wgt
NPA-BHON	TAC-SBCL-ON	IABP Preop	0.01	0.02	0.12	0.05	0.58	0.01	wgt

NPA-BHON	TAC-SBCL-ON	LVEF	53.79	52.91	11.36	0.08	0.28	0.07	wgt
NPA-BHON	TAC-SBCL-ON	LMCA Stenosis	0.25	0.33	0.47	0.17	0.05	0.08	wgt
NPA-BHON	TAC-SBCL-ON	Two Vessels Disease	0.37	0.34	0.47	0.05	0.58	0.02	wgt
NPA-BHON	TAC-SBCL-ON	Previuos PTCA	0.32	0.33	0.46	0.03	0.78	0.01	wgt
NPA-BHON	TAC-SBCL-ON	Antiplatelet Drugs	0.36	0.36	0.47	0.01	0.94	0	wgt
NPA-BHON	TAC-SBCL-ON	Surgery	0.05	0.09	0.28	0.13	0.07	0.04	wgt
NPA-BHON	TAC-SBCL-ON	REDO	0.05	0.04	0.2	0.03	0.7	0.01	wgt
NPA-BHON	TAC-SBCL-ON	NYHA ≥ 3	0.09	0.13	0.36	0.12	0.19	0.04	wgt
NPA-BHON	TAC-SBCL-ON	≥ 3-Vessel Disease	0.51	0.63	0.49	0.13	0.01	0.12	wgt
NPA-BHON	TAC-SBCL-ON	LAD Stenosis	0.97	0.95	0.24	0.06	0.16	0.01	wgt
SBCL-BHON	NPA-TAC-ON	Age	67.48	66.63	9.53	0.09	0.34	0.06	wgt
SBCL-BHON	NPA-TAC-ON	Gender(Male)	0.84	0.81	0.4	0.07	0.28	0.03	wgt
SBCL-BHON	NPA-TAC-ON	Hypertension	0.65	0.53	0.49	0.14	0.02	0.12	wgt
SBCL-BHON	NPA-TAC-ON	Smoker	0.4	0.34	0.48	0.12	0.16	0.06	wgt
SBCL-BHON	NPA-TAC-ON	Hypercholesterolemia	0.5	0.42	0.5	0.17	0.08	0.08	wgt
SBCL-BHON	NPA-TAC-ON	Diabetes	0.25	0.22	0.43	0.07	0.35	0.03	wgt
SBCL-BHON	NPA-TAC-ON	Insulin Dependent Diabetes	0.1	0.08	0.29	0.09	0.19	0.02	wgt
SBCL-BHON	NPA-TAC-ON	COPD	0.06	0.07	0.25	0.04	0.66	0.01	wgt
SBCL-BHON	NPA-TAC-ON	AKD	0.03	0.03	0.2	0	0.98	0	wgt
SBCL-BHON	NPA-TAC-ON	Dialysis	0	0.01	0.09	0.01	0.71	0	wgt
SBCL-BHON	NPA-TAC-ON	Stroke History	0.01	0.02	0.17	0.09	0.18	0.01	wgt
SBCL-BHON	NPA-TAC-ON	TIA History	0.01	0.01	0.17	0.02	0.61	0	wgt
SBCL-BHON	NPA-TAC-ON	Carotid Stenosis > 50%	0.03	0.07	0.27	0.15	0.01	0.04	wgt
SBCL-BHON	NPA-TAC-ON	PAD	0.17	0.16	0.38	0.05	0.54	0.02	wgt
SBCL-BHON	NPA-TAC-ON	Pulmonary Hypertension	0.04	0.04	0.21	0.01	0.95	0	wgt
SBCL-BHON	NPA-TAC-ON	IABP Preop	0.01	0.01	0.12	0.04	0.44	0	wgt
SBCL-BHON	NPA-TAC-ON	LVEF	52.71	53.22	11.36	0.04	0.56	0.05	wgt
SBCL-BHON	NPA-TAC-ON	LMCA Stenosis	0.37	0.33	0.47	0.08	0.43	0.04	wgt
SBCL-BHON	NPA-TAC-ON	Two Vessels Disease	0.33	0.36	0.47	0.07	0.36	0.04	wgt
SBCL-BHON	NPA-TAC-ON	Previuos PTCA	0.28	0.26	0.46	0.03	0.77	0.01	wgt
SBCL-BHON	NPA-TAC-ON	Antiplatelet Drugs	0.35	0.3	0.47	0.09	0.31	0.04	wgt
SBCL-BHON	NPA-TAC-ON	Surgery	0.06	0.07	0.28	0.05	0.35	0.01	wgt

SBCL-BHON	NPA-TAC-ON	REDO	0	0.05	0.2	0.15	0	0.05	wgt
SBCL-BHON	NPA-TAC-ON	NYHA ≥ 3	0.16	0.1	0.36	0.17	0.02	0.06	wgt
SBCL-BHON	NPA-TAC-ON	≥ 3-Vessel Disease	0.59	0.58	0.49	0.02	0.84	0.01	wgt
SBCL-BHON	NPA-TAC-ON	LAD Stenosis	0.93	0.94	0.24	0.04	0.86	0.01	wgt
SBCL-BHON	SINGLE TAC-ON	Age	67.48	66.73	9.53	0.08	0.35	0.06	wgt
SBCL-BHON	SINGLE TAC-ON	Gender(Male)	0.84	0.79	0.4	0.12	0.02	0.05	wgt
SBCL-BHON	SINGLE TAC-ON	Hypertension	0.65	0.6	0.49	0.11	0.25	0.05	wgt
SBCL-BHON	SINGLE TAC-ON	Smoker	0.4	0.37	0.48	0.06	0.38	0.03	wgt
SBCL-BHON	SINGLE TAC-ON	Hypercholesterolemia	0.5	0.46	0.5	0.09	0.25	0.05	wgt
SBCL-BHON	SINGLE TAC-ON	Diabetes	0.25	0.26	0.43	0.01	0.86	0	wgt
SBCL-BHON	SINGLE TAC-ON	Insulin Dependent Diabetes	0.1	0.09	0.29	0.02	0.63	0.01	wgt
SBCL-BHON	SINGLE TAC-ON	COPD	0.06	0.07	0.25	0.06	0.45	0.02	wgt
SBCL-BHON	SINGLE TAC-ON	AKD	0.03	0.04	0.2	0.08	0.35	0.02	wgt
SBCL-BHON	SINGLE TAC-ON	Dialysis	0	0.01	0.09	0.03	0.24	0	wgt
SBCL-BHON	SINGLE TAC-ON	Stroke History	0.01	0.03	0.17	0.13	0.01	0.02	wgt
SBCL-BHON	SINGLE TAC-ON	TIA History	0.01	0.03	0.17	0.1	0	0.02	wgt
SBCL-BHON	SINGLE TAC-ON	Carotid Stenosis > 50%	0.03	0.08	0.27	0.16	0	0.06	wgt
SBCL-BHON	SINGLE TAC-ON	PAD	0.17	0.18	0.38	0.03	0.68	0.01	wgt
SBCL-BHON	SINGLE TAC-ON	Pulmonary Hypertension	0.04	0.05	0.21	0.06	0.44	0.01	wgt
SBCL-BHON	SINGLE TAC-ON	IABP Preop	0.01	0.01	0.12	0.04	0.27	0	wgt
SBCL-BHON	SINGLE TAC-ON	LVEF	52.71	52.42	11.36	0.03	0.69	0.06	wgt
SBCL-BHON	SINGLE TAC-ON	LMCA Stenosis	0.37	0.33	0.47	0.08	0.4	0.04	wgt
SBCL-BHON	SINGLE TAC-ON	Two Vessels Disease	0.33	0.33	0.47	0.02	0.78	0.01	wgt
SBCL-BHON	SINGLE TAC-ON	Previuos PTCA	0.28	0.3	0.46	0.05	0.51	0.02	wgt
SBCL-BHON	SINGLE TAC-ON	Antiplatelet Drugs	0.35	0.33	0.47	0.04	0.6	0.02	wgt
SBCL-BHON	SINGLE TAC-ON	Surgery	0.06	0.08	0.28	0.09	0.03	0.03	wgt
SBCL-BHON	SINGLE TAC-ON	REDO	0	0.05	0.2	0.15	0	0.04	wgt
SBCL-BHON	SINGLE TAC-ON	NYHA ≥ 3	0.16	0.13	0.36	0.09	0.14	0.03	wgt
SBCL-BHON	SINGLE TAC-ON	≥ 3-Vessel Disease	0.59	0.62	0.49	0.06	0.53	0.03	wgt
SBCL-BHON	SINGLE TAC-ON	LAD Stenosis	0.93	0.94	0.24	0.05	0.81	0.01	wgt
SBCL-BHON	TAC-SBCL-ON	Age	67.48	66.89	9.53	0.06	0.45	0.05	wgt
SBCL-BHON	TAC-SBCL-ON	Gender(Male)	0.84	0.8	0.4	0.1	0.05	0.04	wgt

SBCL-BHON	TAC-SBCL-ON	Hypertension	0.65	0.56	0.49	0.09	0.04	0.09	wgt
SBCL-BHON	TAC-SBCL-ON	Smoker	0.4	0.33	0.48	0.14	0.04	0.07	wgt
SBCL-BHON	TAC-SBCL-ON	Hypercholesterolemia	0.5	0.4	0.5	0.15	0.01	0.1	wgt
SBCL-BHON	TAC-SBCL-ON	Diabetes	0.25	0.25	0.43	0.01	0.82	0.01	wgt
SBCL-BHON	TAC-SBCL-ON	Insulin Dependent Diabetes	0.1	0.08	0.29	0.06	0.32	0.02	wgt
SBCL-BHON	TAC-SBCL-ON	COPD	0.06	0.07	0.25	0.04	0.6	0.01	wgt
SBCL-BHON	TAC-SBCL-ON	AKD	0.03	0.04	0.2	0.05	0.5	0.01	wgt
SBCL-BHON	TAC-SBCL-ON	Dialysis	0	0.01	0.09	0.03	0.19	0	wgt
SBCL-BHON	TAC-SBCL-ON	Stroke History	0.01	0.02	0.17	0.07	0.12	0.01	wgt
SBCL-BHON	TAC-SBCL-ON	TIA History	0.01	0.03	0.17	0.1	0	0.02	wgt
SBCL-BHON	TAC-SBCL-ON	Carotid Stenosis > 50%	0.03	0.08	0.27	0.15	0	0.05	wgt
SBCL-BHON	TAC-SBCL-ON	PAD	0.17	0.17	0.38	0.02	0.74	0.01	wgt
SBCL-BHON	TAC-SBCL-ON	Pulmonary Hypertension	0.04	0.05	0.21	0.08	0.37	0.02	wgt
SBCL-BHON	TAC-SBCL-ON	IABP Preop	0.01	0.02	0.12	0.05	0.16	0.01	wgt
SBCL-BHON	TAC-SBCL-ON	LVEF	52.71	52.91	11.36	0.02	0.78	0.08	wgt
SBCL-BHON	TAC-SBCL-ON	LMCA Stenosis	0.37	0.33	0.47	0.07	0.42	0.04	wgt
SBCL-BHON	TAC-SBCL-ON	Two Vessels Disease	0.33	0.34	0.47	0.04	0.56	0.02	wgt
SBCL-BHON	TAC-SBCL-ON	Previuos PTCA	0.28	0.33	0.46	0.12	0.07	0.06	wgt
SBCL-BHON	TAC-SBCL-ON	Antiplatelet Drugs	0.35	0.36	0.47	0.03	0.7	0.01	wgt
SBCL-BHON	TAC-SBCL-ON	Surgery	0.06	0.09	0.28	0.13	0.01	0.04	wgt
SBCL-BHON	TAC-SBCL-ON	REDO	0	0.04	0.2	0.15	0	0.04	wgt
SBCL-BHON	TAC-SBCL-ON	NYHA ≥ 3	0.16	0.13	0.36	0.09	0.1	0.03	wgt
SBCL-BHON	TAC-SBCL-ON	≥ 3-Vessel Disease	0.59	0.63	0.49	0.09	0.29	0.04	wgt
SBCL-BHON	TAC-SBCL-ON	LAD Stenosis	0.93	0.95	0.24	0.1	0.64	0.03	wgt
NPA-TAC-ON	SINGLE TAC-ON	Age	66.63	66.73	9.53	0.01	0.85	0.02	wgt
NPA-TAC-ON	SINGLE TAC-ON	Gender(Male)	0.81	0.79	0.4	0.05	0.37	0.02	wgt
NPA-TAC-ON	SINGLE TAC-ON	Hypertension	0.53	0.6	0.49	0.14	0.03	0.07	wgt
NPA-TAC-ON	SINGLE TAC-ON	Smoker	0.34	0.37	0.48	0.06	0.32	0.03	wgt
NPA-TAC-ON	SINGLE TAC-ON	Hypercholesterolemia	0.42	0.46	0.5	0.08	0.23	0.04	wgt
NPA-TAC-ON	SINGLE TAC-ON	Diabetes	0.22	0.26	0.43	0.08	0.15	0.04	wgt
NPA-TAC-ON	SINGLE TAC-ON	Insulin Dependent Diabetes	0.08	0.09	0.29	0.06	0.28	0.02	wgt
NPA-TAC-ON	SINGLE TAC-ON	COPD	0.07	0.07	0.25	0.02	0.74	0	wgt

NPA-TAC-ON	SINGLE TAC-ON	AKD	0.03	0.04	0.2	0.07	0.13	0.02	wgt
NPA-TAC-ON	SINGLE TAC-ON	Dialysis	0.01	0.01	0.09	0.02	0.64	0	wgt
NPA-TAC-ON	SINGLE TAC-ON	Stroke History	0.02	0.03	0.17	0.04	0.4	0.01	wgt
NPA-TAC-ON	SINGLE TAC-ON	TIA History	0.01	0.03	0.17	0.09	0.02	0.01	wgt
NPA-TAC-ON	SINGLE TAC-ON	Carotid Stenosis > 50%	0.07	0.08	0.27	0.07	0.2	0.02	wgt
NPA-TAC-ON	SINGLE TAC-ON	PAD	0.16	0.18	0.38	0.07	0.21	0.03	wgt
NPA-TAC-ON	SINGLE TAC-ON	Pulmonary Hypertension	0.04	0.05	0.21	0.06	0.23	0.01	wgt
NPA-TAC-ON	SINGLE TAC-ON	IABP Preop	0.01	0.01	0.12	0	0.99	0	wgt
NPA-TAC-ON	SINGLE TAC-ON	LVEF	53.22	52.42	11.36	0.07	0.18	0.05	wgt
NPA-TAC-ON	SINGLE TAC-ON	LMCA Stenosis	0.33	0.33	0.47	0.01	0.91	0	wgt
NPA-TAC-ON	SINGLE TAC-ON	Two Vessels Disease	0.36	0.33	0.47	0.05	0.34	0.03	wgt
NPA-TAC-ON	SINGLE TAC-ON	Previous PTCA	0.26	0.3	0.46	0.07	0.29	0.03	wgt
NPA-TAC-ON	SINGLE TAC-ON	Antiplatelet Drugs	0.3	0.33	0.47	0.05	0.4	0.02	wgt
NPA-TAC-ON	SINGLE TAC-ON	Surgery	0.07	0.08	0.28	0.04	0.32	0.01	wgt
NPA-TAC-ON	SINGLE TAC-ON	REDO	0.05	0.05	0.2	0.02	0.73	0	wgt
NPA-TAC-ON	SINGLE TAC-ON	NYHA ≥ 3	0.1	0.13	0.36	0.09	0.13	0.03	wgt
NPA-TAC-ON	SINGLE TAC-ON	≥ 3-Vessel Disease	0.58	0.62	0.49	0.07	0.2	0.04	wgt
NPA-TAC-ON	SINGLE TAC-ON	LAD Stenosis	0.94	0.94	0.24	0.01	0.85	0	wgt
NPA-TAC-ON	TAC-SBCL-ON	Age	66.63	66.89	9.53	0.03	0.61	0.03	wgt
NPA-TAC-ON	TAC-SBCL-ON	Gender(Male)	0.81	0.8	0.4	0.03	0.59	0.01	wgt
NPA-TAC-ON	TAC-SBCL-ON	Hypertension	0.53	0.56	0.49	0.05	0.37	0.03	wgt
NPA-TAC-ON	TAC-SBCL-ON	Smoker	0.34	0.33	0.48	0.02	0.79	0.01	wgt
NPA-TAC-ON	TAC-SBCL-ON	Hypercholesterolemia	0.42	0.4	0.5	0.03	0.59	0.02	wgt
NPA-TAC-ON	TAC-SBCL-ON	Diabetes	0.22	0.25	0.43	0.06	0.28	0.03	wgt
NPA-TAC-ON	TAC-SBCL-ON	Insulin Dependent Diabetes	0.08	0.08	0.29	0.03	0.64	0.01	wgt
NPA-TAC-ON	TAC-SBCL-ON	COPD	0.07	0.07	0.25	0	0.99	0	wgt
NPA-TAC-ON	TAC-SBCL-ON	AKD	0.03	0.04	0.2	0.05	0.27	0.01	wgt
NPA-TAC-ON	TAC-SBCL-ON	Dialysis	0.01	0.01	0.09	0.02	0.63	0	wgt
NPA-TAC-ON	TAC-SBCL-ON	Stroke History	0.02	0.02	0.17	0.02	0.75	0	wgt
NPA-TAC-ON	TAC-SBCL-ON	TIA History	0.01	0.03	0.17	0.08	0.02	0.01	wgt
NPA-TAC-ON	TAC-SBCL-ON	Carotid Stenosis > 50%	0.07	0.08	0.27	0.05	0.4	0.01	wgt
NPA-TAC-ON	TAC-SBCL-ON	PAD	0.16	0.17	0.38	0.03	0.67	0.01	wgt

NPA-TAC-ON	TAC-SBCL-ON	Pulmonary Hypertension	0.04	0.05	0.21	0.07	0.23	0.02	wgt
NPA-TAC-ON	TAC-SBCL-ON	IABP Preop	0.01	0.02	0.12	0.08	0.09	0.01	wgt
NPA-TAC-ON	TAC-SBCL-ON	LVEF	53.22	52.91	11.36	0.03	0.59	0.03	wgt
NPA-TAC-ON	TAC-SBCL-ON	LMCA Stenosis	0.33	0.33	0.47	0.01	0.88	0	wgt
NPA-TAC-ON	TAC-SBCL-ON	2-Vessel Disease	0.36	0.34	0.47	0.04	0.52	0.02	wgt
NPA-TAC-ON	TAC-SBCL-ON	Previous PTCA	0.26	0.33	0.46	0.15	0.02	0.07	wgt
NPA-TAC-ON	TAC-SBCL-ON	Antiplatelet Drugs	0.3	0.36	0.47	0.12	0.06	0.05	wgt
NPA-TAC-ON	TAC-SBCL-ON	Surgery	0.07	0.09	0.28	0.08	0.09	0.02	wgt
NPA-TAC-ON	TAC-SBCL-ON	REDO	0.05	0.04	0.2	0.03	0.58	0.01	wgt
NPA-TAC-ON	TAC-SBCL-ON	NYHA ≥ 3	0.1	0.13	0.36	0.08	0.09	0.03	wgt
NPA-TAC-ON	TAC-SBCL-ON	≥ 3-Vessel Disease	0.58	0.63	0.49	0.11	0.05	0.05	wgt
NPA-TAC-ON	TAC-SBCL-ON	LAD Stenosis	0.94	0.95	0.24	0.06	0.31	0.02	wgt
SINGLE TAC-ON	TAC-SBCL-ON	Age	66.73	66.89	9.53	0.02	0.59	0.03	wgt
SINGLE TAC-ON	TAC-SBCL-ON	Gender(Male)	0.79	0.8	0.4	0.02	0.56	0.01	wgt
SINGLE TAC-ON	TAC-SBCL-ON	Hypertension	0.6	0.56	0.49	0.09	0.01	0.04	wgt
SINGLE TAC-ON	TAC-SBCL-ON	Smoker	0.37	0.33	0.48	0.08	0.01	0.04	wgt
SINGLE TAC-ON	TAC-SBCL-ON	Hypercholesterolemia	0.46	0.4	0.5	0.11	0	0.05	wgt
SINGLE TAC-ON	TAC-SBCL-ON	Diabetes	0.26	0.25	0.43	0.02	0.46	0.01	wgt
SINGLE TAC-ON	TAC-SBCL-ON	Insulin Dependent Diabetes	0.09	0.08	0.29	0.03	0.49	0.01	wgt
SINGLE TAC-ON	TAC-SBCL-ON	COPD	0.07	0.07	0.25	0.02	0.52	0	wgt
SINGLE TAC-ON	TAC-SBCL-ON	AKD	0.04	0.04	0.2	0.02	0.44	0	wgt
SINGLE TAC-ON	TAC-SBCL-ON	Dialysis	0.01	0.01	0.09	0	0.96	0	wgt
SINGLE TAC-ON	TAC-SBCL-ON	Stroke History	0.03	0.02	0.17	0.06	0.02	0.01	wgt
SINGLE TAC-ON	TAC-SBCL-ON	TIA History	0.03	0.03	0.17	0	0.94	0	wgt
SINGLE TAC-ON	TAC-SBCL-ON	Carotid Stenosis > 50%	0.08	0.08	0.27	0.01	0.73	0	wgt
SINGLE TAC-ON	TAC-SBCL-ON	PAD	0.18	0.17	0.38	0.05	0.2	0.02	wgt
SINGLE TAC-ON	TAC-SBCL-ON	Pulmonary Hypertension	0.05	0.05	0.21	0.02	0.7	0	wgt
SINGLE TAC-ON	TAC-SBCL-ON	IABP Preop	0.01	0.02	0.12	0.08	0.02	0.01	wgt
SINGLE TAC-ON	TAC-SBCL-ON	LVEF	52.42	52.91	11.36	0.04	0.15	0.04	wgt
SINGLE TAC-ON	TAC-SBCL-ON	LMCA Stenosis	0.33	0.33	0.47	0	0.92	0	wgt
SINGLE TAC-ON	TAC-SBCL-ON	Two Vessels Disease	0.33	0.34	0.47	0.02	0.55	0.01	wgt
SINGLE TAC-ON	TAC-SBCL-ON	Previuos PTCA	0.3	0.33	0.46	0.07	0.03	0.03	wgt

SINGLE TAC-ON	TAC-SBCL-ON	Antiplatelet Drugs	0.33	0.36	0.47	0.06	0.07	0.03	wgt
SINGLE TAC-ON	TAC-SBCL-ON	Surgery	0.08	0.09	0.28	0.03	0.21	0.01	wgt
SINGLE TAC-ON	TAC-SBCL-ON	REDO	0.05	0.04	0.2	0.01	0.63	0	wgt
SINGLE TAC-ON	TAC-SBCL-ON	NYHA ≥ 3	0.13	0.13	0.36	0	0.97	0	wgt
SINGLE TAC-ON	TAC-SBCL-ON	≥ 3-Vessel Disease	0.62	0.63	0.49	0.04	0.27	0.02	wgt
SINGLE TAC-ON	TAC-SBCL-ON	LAD Stenosis	0.94	0.95	0.24	0.05	0.03	0.01	wgt

Abbreviations: tmt1: Treatment 1, tmt2: Treatmen 2, var: Variables, pop.sd: Standard Deviation Beetwen Treatments, Std.eff.sz: Standardized Effect Size, ks: Kolmogorov–Smirnov test, NPA-OFF: Non-Touch Off-Pump; SBCL-OFF: Side-Clamp Off-Pump; NPA-BHON: No Proximal Anastomoses Beating Heart On-Pump; SBCL-BHON: Side-Clamp Beating Heart On-Pump; NPA-TAC-ON: No Proximal Anastomoses On-Pump; SINGLE TAC-ON: Single Total Clamp On-Pump; TAC-SBCL-ON: Side-Clamp On-Pump; COPD: Chronic Obstructive Pulmonary Disease; AKD: Acute Kidney Disease; TIA: Transient Ischemic Attack; PAD: Peripheral Arterial Disease; IABP: Intra-Aortic Balloon Pump; NYHA: New York Heart Association Score; LVEF: Left Ventricular Ejection Fraction; LMCA: Left Main Coronary Artery; PTCA: Percutaneous Transluminal Coronary Angioplasty; unw: unweighted; wgt: weighted.

Tab. S12. Balance Table Model 2 by-Number of Aortic Touches

tmt1	tmt2	var	mean1	mean2	pop.sd	std.eff.sz	p	ks	Stop Method
0	1	Age	66.12	67.89	9.53	0.19	0	0.08	unw
0	1	Gender(Male)	0.8	0.82	0.4	0.06	0.11	0.02	unw
0	1	Hypertension	0.27	0.35	0.49	0.16	0	0.08	unw
0	1	Smoker	0.14	0.12	0.48	0.04	0.14	0.02	unw
0	1	Hypercholesterolemia	0.14	0.15	0.5	0.01	0.78	0	unw
0	1	Diabetes	0.17	0.22	0.43	0.11	0	0.05	unw
0	1	Insulin Dependent Diabetes	0.05	0.03	0.29	0.09	0	0.03	unw
0	1	COPD	0.04	0.06	0.25	0.08	0.01	0.02	unw
0	1	AKD	0.03	0.03	0.2	0.02	0.46	0	unw
0	1	Dialysis	0.01	0	0.09	0.01	0.79	0	unw
0	1	Stroke History	0.01	0.01	0.17	0.01	0.71	0	unw
0	1	TIA History	0.01	0.01	0.17	0	0.95	0	unw
0	1	Carotid Stenosis > 50%	0.05	0.08	0.28	0.1	0	0.03	unw
0	1	PAD	0.07	0.07	0.38	0.02	0.41	0.01	unw
0	1	Pulmonary Hypertension	0.02	0.03	0.22	0.06	0.01	0.01	unw
0	1	IABP Preop	0.02	0.02	0.12	0.02	0.68	0	unw
0	1	LVEF	55.58	55.34	11.38	0.02	0.55	0.07	unw
0	1	LMCA Stenosis	0.21	0.32	0.47	0.23	0	0.11	unw
0	1	2-Vessel Disease	0.47	0.37	0.47	0.22	0	0.1	unw
0	1	Previous PTCA	0.11	0.24	0.46	0.27	0	0.13	unw
0	1	Antiplatelet Drugs	0.22	0.3	0.46	0.17	0	0.08	unw
0	1	Surgery	0.03	0.04	0.28	0.04	0.12	0.01	unw
0	1	REDO	0.04	0.03	0.19	0.07	0.06	0.01	unw
0	1	NYHA ≥ 3	0.11	0.08	0.36	0.08	0.01	0.03	unw
0	1	≥ 3-Vessel Disease	0.16	0.54	0.49	0.78	0	0.38	unw
0	1	LAD Stenosis	0.77	0.98	0.24	0.87	0	0.21	unw
0	2	Age	66.12	66.6	9.53	0.05	0.22	0.03	unw
0	2	Gender(Male)	0.8	0.82	0.4	0.06	0.15	0.02	unw
0	2	Hypertension	0.27	0.8	0.49	1.08	0	0.53	unw
0	2	Smoker	0.14	0.55	0.48	0.84	0	0.41	unw

0	2	Hypercholesterolemia	0.14	0.73	0.5	1.18	0	0.58	unw
0	2	Diabetes	0.17	0.37	0.43	0.45	0	0.19	unw
0	2	Insulin Dependent Diabetes	0.05	0.2	0.29	0.51	0	0.15	unw
0	2	COPD	0.04	0.02	0.25	0.09	0	0.02	unw
0	2	AKD	0.03	0.02	0.2	0.09	0.01	0.02	unw
0	2	Dialysis	0.01	0.01	0.09	0.06	0.1	0.01	unw
0	2	Stroke History	0.01	0.01	0.17	0.05	0.03	0.01	unw
0	2	TIA History	0.01	0.02	0.17	0.02	0.55	0	unw
0	2	Carotid Stenosis > 50%	0.05	0.02	0.28	0.12	0	0.03	unw
0	2	PAD	0.07	0.24	0.38	0.43	0	0.16	unw
0	2	Pulmonary Hypertension	0.02	0.01	0.22	0.02	0.34	0	unw
0	2	IABP Preop	0.02	0.03	0.12	0.13	0.01	0.02	unw
0	2	LVEF	55.58	52.22	11.38	0.29	0	0.23	unw
0	2	LMCA Stenosis	0.21	0.35	0.47	0.29	0	0.14	unw
0	2	2-Vessel Disease	0.47	0.22	0.47	0.53	0	0.25	unw
0	2	Previous PTCA	0.11	0.16	0.46	0.11	0	0.05	unw
0	2	Antiplatelet Drugs	0.22	0.32	0.46	0.2	0	0.09	unw
0	2	Surgery	0.03	0.06	0.28	0.1	0	0.03	unw
0	2	REDO	0.04	0	0.19	0.19	0	0.04	unw
0	2	NYHA ≥ 3	0.11	0.29	0.36	0.52	0	0.19	unw
0	2	≥ 3-Vessel Disease	0.16	0.76	0.49	1.22	0	0.6	unw
0	2	LAD Stenosis	0.77	1	0.24	0.93	0	0.23	unw
0	3	Age	66.12	65.89	9.53	0.02	0.48	0.03	unw
0	3	Gender(Male)	0.8	0.78	0.4	0.04	0.22	0.02	unw
0	3	Hypertension	0.27	0.65	0.49	0.77	0	0.38	unw
0	3	Smoker	0.14	0.45	0.48	0.65	0	0.31	unw
0	3	Hypercholesterolemia	0.14	0.51	0.5	0.73	0	0.36	unw
0	3	Diabetes	0.17	0.19	0.43	0.04	0.14	0.02	unw
0	3	Insulin Dependent Diabetes	0.05	0.11	0.29	0.2	0	0.06	unw
0	3	COPD	0.04	0.09	0.25	0.17	0	0.04	unw
0	3	AKD	0.03	0.05	0.2	0.06	0.07	0.01	unw
0	3	Dialysis	0.01	0.01	0.09	0.05	0.08	0	unw

0	3	Stroke History	0.01	0.06	0.17	0.27	0	0.04	unw
0	3	TIA History	0.01	0.04	0.17	0.13	0	0.02	unw
0	3	Carotid Stenosis > 50%	0.05	0.17	0.28	0.42	0	0.12	unw
0	3	PAD	0.07	0.22	0.38	0.38	0	0.14	unw
0	3	Pulmonary Hypertension	0.02	0.09	0.22	0.33	0	0.07	unw
0	3	IABP Preop	0.02	0.01	0.12	0.08	0.03	0.01	unw
0	3	LVEF	55.58	50.99	11.38	0.4	0	0.22	unw
0	3	LMCA Stenosis	0.21	0.35	0.47	0.29	0	0.14	unw
0	3	2-Vessel Disease	0.47	0.31	0.47	0.34	0	0.16	unw
0	3	Previous PTCA	0.11	0.17	0.46	0.12	0	0.06	unw
0	3	Antiplatelet Drugs	0.22	0.33	0.46	0.23	0	0.11	unw
0	3	Surgery	0.03	0.15	0.28	0.43	0	0.12	unw
0	3	REDO	0.04	0.07	0.19	0.16	0	0.03	unw
0	3	NYHA ≥ 3	0.11	0.1	0.36	0.01	0.66	0	unw
0	3	≥ 3-Vessel Disease	0.16	0.63	0.49	0.96	0	0.47	unw
0	3	LAD Stenosis	0.77	0.92	0.24	0.63	0	0.15	unw
0	4	Age	66.12	67.47	9.53	0.14	0	0.07	unw
0	4	Gender(Male)	0.8	0.8	0.4	0.01	0.85	0	unw
0	4	Hypertension	0.27	0.63	0.49	0.73	0	0.36	unw
0	4	Smoker	0.14	0.4	0.48	0.54	0	0.26	unw
0	4	Hypercholesterolemia	0.14	0.5	0.5	0.72	0	0.36	unw
0	4	Diabetes	0.17	0.28	0.43	0.25	0	0.11	unw
0	4	Insulin Dependent Diabetes	0.05	0.09	0.29	0.11	0	0.03	unw
0	4	COPD	0.04	0.07	0.25	0.1	0	0.03	unw
0	4	AKD	0.03	0.05	0.2	0.1	0	0.02	unw
0	4	Dialysis	0.01	0.01	0.09	0.04	0.14	0	unw
0	4	Stroke History	0.01	0.02	0.17	0.03	0.19	0.01	unw
0	4	TIA History	0.01	0.04	0.17	0.16	0	0.03	unw
0	4	Carotid Stenosis > 50%	0.05	0.04	0.28	0.03	0.21	0.01	unw
0	4	PAD	0.07	0.18	0.38	0.28	0	0.1	unw
0	4	Pulmonary Hypertension	0.02	0.04	0.22	0.12	0	0.03	unw
0	4	IABP Preop	0.02	0.02	0.12	0.01	0.77	0	unw

0	4	LVEF	55.58	52.06	11.38	0.31	0	0.14	unw
0	4	LMCA Stenosis	0.21	0.33	0.47	0.25	0	0.12	unw
0	4	2-Vessel Disease	0.47	0.35	0.47	0.27	0	0.13	unw
0	4	Previous PTCA	0.11	0.54	0.46	0.92	0	0.43	unw
0	4	Antiplatelet Drugs	0.22	0.32	0.46	0.22	0	0.1	unw
0	4	Surgery	0.03	0.07	0.28	0.11	0	0.03	unw
0	4	REDO	0.04	0.03	0.19	0.05	0.1	0.01	unw
0	4	NYHA ≥ 3	0.11	0.2	0.36	0.26	0	0.09	unw
0	4	≥ 3-Vessel Disease	0.16	0.66	0.49	1.01	0	0.49	unw
0	4	LAD Stenosis	0.77	0.94	0.24	0.71	0	0.17	unw
1	2	Age	67.89	66.6	9.53	0.14	0	0.05	unw
1	2	Gender(Male)	0.82	0.82	0.4	0	0.95	0	unw
1	2	Hypertension	0.35	0.8	0.49	0.92	0	0.45	unw
1	2	Smoker	0.12	0.55	0.48	0.88	0	0.43	unw
1	2	Hypercholesterolemia	0.15	0.73	0.5	1.17	0	0.58	unw
1	2	Diabetes	0.22	0.37	0.43	0.33	0	0.14	unw
1	2	Insulin Dependent Diabetes	0.03	0.2	0.29	0.6	0	0.17	unw
1	2	COPD	0.06	0.02	0.25	0.17	0	0.04	unw
1	2	AKD	0.03	0.02	0.2	0.07	0	0.01	unw
1	2	Dialysis	0	0.01	0.09	0.07	0.03	0.01	unw
1	2	Stroke History	0.01	0.01	0.17	0.04	0.01	0.01	unw
1	2	TIA History	0.01	0.02	0.17	0.02	0.42	0	unw
1	2	Carotid Stenosis > 50%	0.08	0.02	0.28	0.22	0	0.06	unw
1	2	PAD	0.07	0.24	0.38	0.45	0	0.17	unw
1	2	Pulmonary Hypertension	0.03	0.01	0.22	0.09	0	0.02	unw
1	2	IABP Preop	0.02	0.03	0.12	0.15	0	0.02	unw
1	2	LVEF	55.34	52.22	11.38	0.27	0	0.21	unw
1	2	LMCA Stenosis	0.32	0.35	0.47	0.06	0.06	0.03	unw
1	2	2-Vessel Disease	0.37	0.22	0.47	0.31	0	0.15	unw
1	2	Previous PTCA	0.24	0.16	0.46	0.16	0	0.08	unw
1	2	Antiplatelet Drugs	0.3	0.32	0.46	0.03	0.34	0.01	unw
1	2	Surgery	0.04	0.06	0.28	0.06	0.02	0.02	unw

1	2	REDO	0.03	0	0.19	0.13	0	0.02	unw
1	2	NYHA ≥ 3	0.08	0.29	0.36	0.6	0	0.22	unw
1	2	≥ 3-Vessel Disease	0.54	0.76	0.49	0.44	0	0.22	unw
1	2	LAD Stenosis	0.98	1	0.24	0.06	0	0.01	unw
1	3	Age	67.89	65.89	9.53	0.21	0	0.09	unw
1	3	Gender(Male)	0.82	0.78	0.4	0.1	0	0.04	unw
1	3	Hypertension	0.35	0.65	0.49	0.61	0	0.3	unw
1	3	Smoker	0.12	0.45	0.48	0.69	0	0.33	unw
1	3	Hypercholesterolemia	0.15	0.51	0.5	0.73	0	0.36	unw
1	3	Diabetes	0.22	0.19	0.43	0.07	0	0.03	unw
1	3	Insulin Dependent Diabetes	0.03	0.11	0.29	0.29	0	0.09	unw
1	3	COPD	0.06	0.09	0.25	0.09	0	0.02	unw
1	3	AKD	0.03	0.05	0.2	0.08	0	0.02	unw
1	3	Dialysis	0	0.01	0.09	0.06	0.01	0.01	unw
1	3	Stroke History	0.01	0.06	0.17	0.28	0	0.05	unw
1	3	TIA History	0.01	0.04	0.17	0.13	0	0.02	unw
1	3	Carotid Stenosis > 50%	0.08	0.17	0.28	0.32	0	0.09	unw
1	3	PAD	0.07	0.22	0.38	0.4	0	0.15	unw
1	3	Pulmonary Hypertension	0.03	0.09	0.22	0.26	0	0.06	unw
1	3	IABP Preop	0.02	0.01	0.12	0.06	0.01	0.01	unw
1	3	LVEF	55.34	50.99	11.38	0.38	0	0.25	unw
1	3	LMCA Stenosis	0.32	0.35	0.47	0.06	0.01	0.03	unw
1	3	2-Vessel Disease	0.37	0.31	0.47	0.12	0	0.06	unw
1	3	Previous PTCA	0.24	0.17	0.46	0.15	0	0.07	unw
1	3	Antiplatelet Drugs	0.3	0.33	0.46	0.06	0.01	0.03	unw
1	3	Surgery	0.04	0.15	0.28	0.39	0	0.11	unw
1	3	REDO	0.03	0.07	0.19	0.23	0	0.04	unw
1	3	NYHA ≥ 3	0.08	0.1	0.36	0.07	0	0.02	unw
1	3	≥ 3-Vessel Disease	0.54	0.63	0.49	0.18	0	0.09	unw
1	3	LAD Stenosis	0.98	0.92	0.24	0.25	0	0.06	unw
1	4	Age	67.89	67.47	9.53	0.04	0.06	0.02	unw
1	4	Gender(Male)	0.82	0.8	0.4	0.05	0.02	0.02	unw

1	4	Hypertension	0.35	0.63	0.49	0.58	0	0.28	unw
1	4	Smoker	0.12	0.4	0.48	0.58	0	0.28	unw
1	4	Hypercholesterolemia	0.15	0.5	0.5	0.71	0	0.35	unw
1	4	Diabetes	0.22	0.28	0.43	0.14	0	0.06	unw
1	4	Insulin Dependent Diabetes	0.03	0.09	0.29	0.2	0	0.06	unw
1	4	COPD	0.06	0.07	0.25	0.03	0.25	0.01	unw
1	4	AKD	0.03	0.05	0.2	0.12	0	0.02	unw
1	4	Dialysis	0	0.01	0.09	0.05	0.01	0	unw
1	4	Stroke History	0.01	0.02	0.17	0.04	0.01	0.01	unw
1	4	TIA History	0.01	0.04	0.17	0.16	0	0.03	unw
1	4	Carotid Stenosis > 50%	0.08	0.04	0.28	0.13	0	0.04	unw
1	4	PAD	0.07	0.18	0.38	0.3	0	0.11	unw
1	4	Pulmonary Hypertension	0.03	0.04	0.22	0.06	0	0.01	unw
1	4	IABP Preop	0.02	0.02	0.12	0.01	0.83	0	unw
1	4	LVEF	55.34	52.06	11.38	0.29	0	0.14	unw
1	4	LMCA Stenosis	0.32	0.33	0.47	0.03	0.25	0.01	unw
1	4	2-Vessel Disease	0.37	0.35	0.47	0.05	0.04	0.02	unw
1	4	Previous PTCA	0.24	0.54	0.46	0.65	0	0.3	unw
1	4	Antiplatelet Drugs	0.3	0.32	0.46	0.05	0.04	0.02	unw
1	4	Surgery	0.04	0.07	0.28	0.08	0	0.02	unw
1	4	REDO	0.03	0.03	0.19	0.01	0.49	0	unw
1	4	NYHA ≥ 3	0.08	0.2	0.36	0.34	0	0.12	unw
1	4	≥ 3-Vessel Disease	0.54	0.66	0.49	0.23	0	0.11	unw
1	4	LAD Stenosis	0.98	0.94	0.24	0.16	0	0.04	unw
2	3	Age	66.6	65.89	9.53	0.07	0.01	0.05	unw
2	3	Gender(Male)	0.82	0.78	0.4	0.1	0	0.04	unw
2	3	Hypertension	0.8	0.65	0.49	0.31	0	0.15	unw
2	3	Smoker	0.55	0.45	0.48	0.19	0	0.09	unw
2	3	Hypercholesterolemia	0.73	0.51	0.5	0.44	0	0.22	unw
2	3	Diabetes	0.37	0.19	0.43	0.4	0	0.17	unw
2	3	Insulin Dependent Diabetes	0.2	0.11	0.29	0.31	0	0.09	unw
2	3	COPD	0.02	0.09	0.25	0.26	0	0.06	unw

2	3	AKD	0.02	0.05	0.2	0.15	0	0.03	unw
2	3	Dialysis	0.01	0.01	0.09	0.01	0.71	0	unw
2	3	Stroke History	0.01	0.06	0.17	0.32	0	0.05	unw
2	3	TIA History	0.02	0.04	0.17	0.12	0	0.02	unw
2	3	Carotid Stenosis > 50%	0.02	0.17	0.28	0.55	0	0.15	unw
2	3	PAD	0.24	0.22	0.38	0.05	0.09	0.02	unw
2	3	Pulmonary Hypertension	0.01	0.09	0.22	0.35	0	0.07	unw
2	3	IABP Preop	0.03	0.01	0.12	0.21	0	0.03	unw
2	3	LVEF	52.22	50.99	11.38	0.11	0	0.17	unw
2	3	LMCA Stenosis	0.35	0.35	0.47	0	0.9	0	unw
2	3	2-Vessel Disease	0.22	0.31	0.47	0.19	0	0.09	unw
2	3	Previuos PTCA	0.16	0.17	0.46	0.01	0.6	0.01	unw
2	3	Antiplatelet Drugs	0.32	0.33	0.46	0.03	0.32	0.01	unw
2	3	Surgery	0.06	0.15	0.28	0.32	0	0.09	unw
2	3	REDO	0	0.07	0.19	0.35	0	0.07	unw
2	3	NYHA ≥ 3	0.29	0.1	0.36	0.53	0	0.19	unw
2	3	≥ 3-Vessel Disease	0.76	0.63	0.49	0.26	0	0.13	unw
2	3	LAD Stenosis	1	0.92	0.24	0.3	0	0.07	unw
2	4	Age	66.6	67.47	9.53	0.09	0	0.04	unw
2	4	Gender(Male)	0.82	0.8	0.4	0.05	0.07	0.02	unw
2	4	Hypertension	0.8	0.63	0.49	0.35	0	0.17	unw
2	4	Smoker	0.55	0.4	0.48	0.3	0	0.15	unw
2	4	Hypercholesterolemia	0.73	0.5	0.5	0.46	0	0.23	unw
2	4	Diabetes	0.37	0.28	0.43	0.19	0	0.08	unw
2	4	Insulin Dependent Diabetes	0.2	0.09	0.29	0.4	0	0.11	unw
2	4	COPD	0.02	0.07	0.25	0.19	0	0.05	unw
2	4	AKD	0.02	0.05	0.2	0.19	0	0.04	unw
2	4	Dialysis	0.01	0.01	0.09	0.02	0.5	0	unw
2	4	Stroke History	0.01	0.02	0.17	0.09	0	0.01	unw
2	4	TIA History	0.02	0.04	0.17	0.14	0	0.02	unw
2	4	Carotid Stenosis > 50%	0.02	0.04	0.28	0.09	0	0.03	unw
2	4	PAD	0.24	0.18	0.38	0.15	0	0.06	unw

2	4	Pulmonary Hypertension	0.01	0.04	0.22	0.14	0	0.03	unw
2	4	IABP Preop	0.03	0.02	0.12	0.14	0	0.02	unw
2	4	LVEF	52.22	52.06	11.38	0.01	0.55	0.11	unw
2	4	LMCA Stenosis	0.35	0.33	0.47	0.03	0.24	0.02	unw
2	4	2-Vessel Disease	0.22	0.35	0.47	0.26	0	0.12	unw
2	4	Previuos PTCA	0.16	0.54	0.46	0.81	0	0.38	unw
2	4	Antiplatelet Drugs	0.32	0.32	0.46	0.02	0.52	0.01	unw
2	4	Surgery	0.06	0.07	0.28	0.01	0.56	0	unw
2	4	REDO	0	0.03	0.19	0.14	0	0.03	unw
2	4	NYHA ≥ 3	0.29	0.2	0.36	0.25	0	0.09	unw
2	4	≥ 3-Vessel Disease	0.76	0.66	0.49	0.21	0	0.1	unw
2	4	LAD Stenosis	1	0.94	0.24	0.22	0	0.05	unw
3	4	Age	65.89	67.47	9.53	0.17	0	0.07	unw
3	4	Gender(Male)	0.78	0.8	0.4	0.05	0.01	0.02	unw
3	4	Hypertension	0.65	0.63	0.49	0.03	0.06	0.02	unw
3	4	Smoker	0.45	0.4	0.48	0.11	0	0.05	unw
3	4	Hypercholesterolemia	0.51	0.5	0.5	0.02	0.37	0.01	unw
3	4	Diabetes	0.19	0.28	0.43	0.21	0	0.09	unw
3	4	Insulin Dependent Diabetes	0.11	0.09	0.29	0.09	0	0.03	unw
3	4	COPD	0.09	0.07	0.25	0.06	0	0.02	unw
3	4	AKD	0.05	0.05	0.2	0.04	0.04	0.01	unw
3	4	Dialysis	0.01	0.01	0.09	0.01	0.65	0	unw
3	4	Stroke History	0.06	0.02	0.17	0.24	0	0.04	unw
3	4	TIA History	0.04	0.04	0.17	0.02	0.28	0	unw
3	4	Carotid Stenosis > 50%	0.17	0.04	0.28	0.45	0	0.13	unw
3	4	PAD	0.22	0.18	0.38	0.1	0	0.04	unw
3	4	Pulmonary Hypertension	0.09	0.04	0.22	0.2	0	0.04	unw
3	4	IABP Preop	0.01	0.02	0.12	0.07	0	0.01	unw
3	4	LVEF	50.99	52.06	11.38	0.09	0	0.11	unw
3	4	LMCA Stenosis	0.35	0.33	0.47	0.04	0.05	0.02	unw
3	4	2-Vessel Disease	0.31	0.35	0.47	0.07	0	0.03	unw
3	4	Previous PTCA	0.17	0.54	0.46	0.8	0	0.37	unw

3	4	Antiplatelet Drugs	0.33	0.32	0.46	0.01	0.6	0	unw
3	4	Surgery	0.15	0.07	0.28	0.31	0	0.09	unw
3	4	REDO	0.07	0.03	0.19	0.21	0	0.04	unw
3	4	NYHA ≥ 3	0.1	0.2	0.36	0.28	0	0.1	unw
3	4	≥ 3-Vessel Disease	0.63	0.66	0.49	0.06	0	0.03	unw
3	4	LAD Stenosis	0.92	0.94	0.24	0.08	0	0.02	unw
0	1	Age	66.38	66.74	9.53	0.04	0.59	0.04	wgt
0	1	Gender(Male)	0.8	0.8	0.4	0	0.99	0	wgt
0	1	Hypertension	0.62	0.6	0.49	0.04	0.51	0.02	wgt
0	1	Smoker	0.38	0.37	0.48	0.03	0.67	0.01	wgt
0	1	Hypercholesterolemia	0.46	0.43	0.5	0.06	0.36	0.03	wgt
0	1	Diabetes	0.27	0.25	0.43	0.04	0.55	0.02	wgt
0	1	Insulin Dependent Diabetes	0.07	0.08	0.29	0.02	0.79	0	wgt
0	1	COPD	0.13	0.06	0.25	0.16	0.01	0.06	wgt
0	1	AKD	0.05	0.05	0.2	0.02	0.82	0	wgt
0	1	Dialysis	0.01	0.01	0.09	0.02	0.66	0	wgt
0	1	Stroke History	0.04	0.02	0.17	0.07	0.33	0.01	wgt
0	1	TIA History	0.03	0.02	0.17	0.05	0.52	0.01	wgt
0	1	Carotid Stenosis > 50%	0.15	0.08	0.28	0.16	0	0.07	wgt
0	1	PAD	0.17	0.17	0.38	0.01	0.85	0.01	wgt
0	1	Pulmonary Hypertension	0.02	0.06	0.22	0.19	0	0.04	wgt
0	1	IABP Preop	0.01	0.02	0.12	0.07	0.15	0.01	wgt
0	1	LVEF	53.49	52.98	11.38	0.05	0.43	0.05	wgt
0	1	LMCA Stenosis	0.27	0.28	0.47	0.03	0.69	0.01	wgt
0	1	2-Vessel Disease	0.28	0.32	0.47	0.1	0.07	0.05	wgt
0	1	Previous PTCA	0.31	0.28	0.46	0.06	0.44	0.03	wgt
0	1	Antiplatelet Drugs	0.28	0.31	0.46	0.07	0.31	0.03	wgt
0	1	Surgery	0.05	0.08	0.28	0.1	0.18	0.03	wgt
0	1	REDO	0.05	0.04	0.19	0.02	0.79	0	wgt
0	1	NYHA ≥ 3	0.14	0.16	0.36	0.05	0.42	0.02	wgt
0	1	≥ 3-Vessel Disease	0.6	0.56	0.49	0.08	0.18	0.04	wgt
0	1	LAD Stenosis	0.91	0.96	0.24	0.18	0	0.04	wgt

0	2	Age	66.38	67.33	9.53	0.1	0.31	0.06	wgt
0	2	Gender(Male)	0.8	0.83	0.4	0.07	0.43	0.03	wgt
0	2	Hypertension	0.62	0.67	0.49	0.09	0.38	0.04	wgt
0	2	Smoker	0.38	0.41	0.48	0.06	0.53	0.03	wgt
0	2	Hypercholesterolemia	0.46	0.51	0.5	0.1	0.3	0.05	wgt
0	2	Diabetes	0.27	0.26	0.43	0.02	0.85	0.01	wgt
0	2	Insulin Dependent Diabetes	0.07	0.1	0.29	0.11	0.09	0.03	wgt
0	2	COPD	0.13	0.06	0.25	0.18	0.03	0.07	wgt
0	2	AKD	0.05	0.03	0.2	0.09	0.44	0.02	wgt
0	2	Dialysis	0.01	0	0.09	0.04	0.41	0	wgt
0	2	Stroke History	0.04	0.01	0.17	0.15	0.04	0.03	wgt
0	2	TIA History	0.03	0.01	0.17	0.13	0.06	0.02	wgt
0	2	Carotid Stenosis > 50%	0.15	0.03	0.28	0.19	0	0.12	wgt
0	2	PAD	0.17	0.19	0.38	0.04	0.67	0.01	wgt
0	2	Pulmonary Hypertension	0.02	0.03	0.22	0.04	0.52	0.01	wgt
0	2	IABP Preop	0.01	0.01	0.12	0.03	0.56	0	wgt
0	2	LVEF	53.49	52.47	11.38	0.09	0.26	0.05	wgt
0	2	LMCA Stenosis	0.27	0.37	0.47	0.19	0.06	0.09	wgt
0	2	2-Vessel Disease	0.28	0.32	0.47	0.09	0.24	0.04	wgt
0	2	Previous PTCA	0.31	0.26	0.46	0.09	0.32	0.04	wgt
0	2	Antiplatelet Drugs	0.28	0.34	0.46	0.14	0.11	0.06	wgt
0	2	Surgery	0.05	0.06	0.28	0.02	0.79	0.01	wgt
0	2	REDO	0.05	0	0.19	0.18	0	0.04	wgt
0	2	NYHA ≥ 3	0.14	0.17	0.36	0.1	0.25	0.03	wgt
0	2	≥ 3-Vessel Disease	0.6	0.57	0.49	0.06	0.52	0.03	wgt
0	2	LAD Stenosis	0.91	0.93	0.24	0.05	0.82	0.01	wgt
0	3	Age	66.38	66.73	9.53	0.04	0.57	0.04	wgt
0	3	Gender(Male)	0.8	0.8	0.4	0.01	0.84	0.01	wgt
0	3	Hypertension	0.62	0.58	0.49	0.1	0.11	0.05	wgt
0	3	Smoker	0.38	0.37	0.48	0.02	0.72	0.01	wgt
0	3	Hypercholesterolemia	0.46	0.43	0.5	0.06	0.4	0.03	wgt
0	3	Diabetes	0.27	0.24	0.43	0.06	0.34	0.03	wgt

0	3	Insulin Dependent Diabetes	0.07	0.08	0.29	0.04	0.46	0.01	wgt
0	3	COPD	0.13	0.07	0.25	0.13	0.02	0.06	wgt
0	3	AKD	0.05	0.04	0.2	0.07	0.29	0.01	wgt
0	3	Dialysis	0.01	0.01	0.09	0	0.95	0	wgt
0	3	Stroke History	0.04	0.03	0.17	0.04	0.55	0.01	wgt
0	3	TIA History	0.03	0.03	0.17	0.02	0.74	0	wgt
0	3	Carotid Stenosis > 50%	0.15	0.09	0.28	0.13	0.01	0.06	wgt
0	3	PAD	0.17	0.16	0.38	0.02	0.72	0.01	wgt
0	3	Pulmonary Hypertension	0.02	0.05	0.22	0.15	0	0.03	wgt
0	3	IABP Preop	0.01	0.01	0.12	0.01	0.89	0	wgt
0	3	LVEF	53.49	52.58	11.38	0.08	0.13	0.06	wgt
0	3	LMCA Stenosis	0.27	0.32	0.47	0.11	0.1	0.05	wgt
0	3	2-Vessel Disease	0.28	0.36	0.47	0.18	0	0.08	wgt
0	3	Previous PTCA	0.31	0.28	0.46	0.06	0.42	0.03	wgt
0	3	Antiplatelet Drugs	0.28	0.32	0.46	0.1	0.11	0.05	wgt
0	3	Surgery	0.05	0.08	0.28	0.12	0.05	0.03	wgt
0	3	REDO	0.05	0.04	0.19	0.02	0.72	0	wgt
0	3	NYHA ≥ 3	0.14	0.13	0.36	0.02	0.73	0.01	wgt
0	3	≥ 3-Vessel Disease	0.6	0.58	0.49	0.05	0.37	0.03	wgt
0	3	LAD Stenosis	0.91	0.94	0.24	0.12	0.03	0.03	wgt
0	4	Age	66.38	66.97	9.53	0.06	0.33	0.05	wgt
0	4	Gender(Male)	0.8	0.8	0.4	0.01	0.89	0	wgt
0	4	Hypertension	0.62	0.56	0.49	0.13	0.03	0.07	wgt
0	4	Smoker	0.38	0.33	0.48	0.11	0.1	0.05	wgt
0	4	Hypercholesterolemia	0.46	0.41	0.5	0.11	0.11	0.05	wgt
0	4	Diabetes	0.27	0.25	0.43	0.05	0.46	0.02	wgt
0	4	Insulin Dependent Diabetes	0.07	0.09	0.29	0.05	0.44	0.01	wgt
0	4	COPD	0.13	0.07	0.25	0.15	0.01	0.06	wgt
0	4	AKD	0.05	0.04	0.2	0.07	0.29	0.01	wgt
0	4	Dialysis	0.01	0.01	0.09	0.02	0.71	0	wgt
0	4	Stroke History	0.04	0.02	0.17	0.08	0.26	0.01	wgt
0	4	TIA History	0.03	0.03	0.17	0.01	0.83	0	wgt

0	4	Carotid Stenosis > 50%	0.15	0.09	0.28	0.13	0.02	0.06	wgt
0	4	PAD	0.17	0.16	0.38	0.03	0.63	0.01	wgt
0	4	Pulmonary Hypertension	0.02	0.05	0.22	0.15	0	0.03	wgt
0	4	IABP Preop	0.01	0.02	0.12	0.06	0.22	0.01	wgt
0	4	LVEF	53.49	52.89	11.38	0.05	0.32	0.05	wgt
0	4	LMCA Stenosis	0.27	0.33	0.47	0.13	0.05	0.06	wgt
0	4	2-Vessel Disease	0.28	0.34	0.47	0.14	0	0.07	wgt
0	4	Previous PTCA	0.31	0.33	0.46	0.05	0.46	0.02	wgt
0	4	Antiplatelet Drugs	0.28	0.35	0.46	0.15	0.02	0.07	wgt
0	4	Surgery	0.05	0.09	0.28	0.15	0.02	0.04	wgt
0	4	REDO	0.05	0.03	0.19	0.06	0.4	0.01	wgt
0	4	NYHA ≥ 3	0.14	0.13	0.36	0.01	0.82	0	wgt
0	4	≥ 3-Vessel Disease	0.6	0.63	0.49	0.06	0.28	0.03	wgt
0	4	LAD Stenosis	0.91	0.95	0.24	0.16	0	0.04	wgt
1	2	Age	66.74	67.33	9.53	0.06	0.46	0.04	wgt
1	2	Gender(Male)	0.8	0.83	0.4	0.07	0.25	0.03	wgt
1	2	Hypertension	0.6	0.67	0.49	0.13	0.14	0.06	wgt
1	2	Smoker	0.37	0.41	0.48	0.09	0.23	0.04	wgt
1	2	Hypercholesterolemia	0.43	0.51	0.5	0.16	0.04	0.08	wgt
1	2	Diabetes	0.25	0.26	0.43	0.02	0.72	0.01	wgt
1	2	Insulin Dependent Diabetes	0.08	0.1	0.29	0.09	0.09	0.03	wgt
1	2	COPD	0.06	0.06	0.25	0.02	0.8	0.01	wgt
1	2	AKD	0.05	0.03	0.2	0.07	0.49	0.01	wgt
1	2	Dialysis	0.01	0	0.09	0.02	0.52	0	wgt
1	2	Stroke History	0.02	0.01	0.17	0.09	0.07	0.01	wgt
1	2	TIA History	0.02	0.01	0.17	0.08	0.02	0.01	wgt
1	2	Carotid Stenosis > 50%	0.08	0.03	0.28	0.16	0	0.04	wgt
1	2	PAD	0.17	0.19	0.38	0.05	0.48	0.02	wgt
1	2	Pulmonary Hypertension	0.06	0.03	0.22	0.16	0.02	0.03	wgt
1	2	IABP Preop	0.02	0.01	0.12	0.05	0.24	0.01	wgt
1	2	LVEF	52.98	52.47	11.38	0.04	0.53	0.07	wgt
1	2	LMCA Stenosis	0.28	0.37	0.47	0.18	0.06	0.08	wgt

1	2	2-Vessel Disease	0.32	0.32	0.47	0.01	0.84	0.01	wgt
1	2	Previuos PTCA	0.28	0.26	0.46	0.03	0.65	0.01	wgt
1	2	Antiplatelet Drugs	0.31	0.34	0.46	0.07	0.32	0.03	wgt
1	2	Surgery	0.08	0.06	0.28	0.08	0.12	0.02	wgt
1	2	REDO	0.04	0	0.19	0.19	0	0.04	wgt
1	2	NYHA ≥ 3	0.16	0.17	0.36	0.04	0.55	0.02	wgt
1	2	≥ 3-Vessel Disease	0.56	0.57	0.49	0.02	0.79	0.01	wgt
1	2	LAD Stenosis	0.96	0.93	0.24	0.13	0.55	0.03	wgt
1	3	Age	66.74	66.73	9.53	0	0.97	0.03	wgt
1	3	Gender(Male)	0.8	0.8	0.4	0.01	0.71	0.01	wgt
1	3	Hypertension	0.6	0.58	0.49	0.06	0.11	0.03	wgt
1	3	Smoker	0.37	0.37	0.48	0.01	0.86	0	wgt
1	3	Hypercholesterolemia	0.43	0.43	0.5	0.01	0.81	0	wgt
1	3	Diabetes	0.25	0.24	0.43	0.02	0.59	0.01	wgt
1	3	Insulin Dependent Diabetes	0.08	0.08	0.29	0.02	0.58	0.01	wgt
1	3	COPD	0.06	0.07	0.25	0.03	0.4	0.01	wgt
1	3	AKD	0.05	0.04	0.2	0.05	0.18	0.01	wgt
1	3	Dialysis	0.01	0.01	0.09	0.02	0.49	0	wgt
1	3	Stroke History	0.02	0.03	0.17	0.03	0.35	0	wgt
1	3	TIA History	0.02	0.03	0.17	0.02	0.51	0	wgt
1	3	Carotid Stenosis > 50%	0.08	0.09	0.28	0.03	0.37	0.01	wgt
1	3	PAD	0.17	0.16	0.38	0.01	0.8	0	wgt
1	3	Pulmonary Hypertension	0.06	0.05	0.22	0.04	0.27	0.01	wgt
1	3	IABP Preop	0.02	0.01	0.12	0.08	0.04	0.01	wgt
1	3	LVEF	52.98	52.58	11.38	0.04	0.33	0.03	wgt
1	3	LMCA Stenosis	0.28	0.32	0.47	0.08	0.02	0.04	wgt
1	3	2-Vessel Disease	0.32	0.36	0.47	0.08	0.04	0.04	wgt
1	3	Previous PTCA	0.28	0.28	0.46	0	0.99	0	wgt
1	3	Antiplatelet Drugs	0.31	0.32	0.46	0.03	0.36	0.02	wgt
1	3	Surgery	0.08	0.08	0.28	0.02	0.55	0.01	wgt
1	3	REDO	0.04	0.04	0.19	0	0.91	0	wgt
1	3	NYHA ≥ 3	0.16	0.13	0.36	0.08	0.07	0.03	wgt

1	3	≥ 3-Vessel Disease	0.56	0.58	0.49	0.03	0.39	0.02	wgt
1	3	LAD Stenosis	0.96	0.94	0.24	0.06	0.13	0.01	wgt
1	4	Age	66.74	66.97	9.53	0.02	0.53	0.02	wgt
1	4	Gender(Male)	0.8	0.8	0.4	0.01	0.82	0	wgt
1	4	Hypertension	0.6	0.56	0.49	0.09	0.01	0.05	wgt
1	4	Smoker	0.37	0.33	0.48	0.08	0.05	0.04	wgt
1	4	Hypercholesterolemia	0.43	0.41	0.5	0.04	0.3	0.02	wgt
1	4	Diabetes	0.25	0.25	0.43	0.01	0.86	0	wgt
1	4	Insulin Dependent Diabetes	0.08	0.09	0.29	0.03	0.54	0.01	wgt
1	4	COPD	0.06	0.07	0.25	0	0.89	0	wgt
1	4	AKD	0.05	0.04	0.2	0.05	0.18	0.01	wgt
1	4	Dialysis	0.01	0.01	0.09	0	0.86	0	wgt
1	4	Stroke History	0.02	0.02	0.17	0.01	0.82	0	wgt
1	4	TIA History	0.02	0.03	0.17	0.03	0.39	0.01	wgt
1	4	Carotid Stenosis > 50%	0.08	0.09	0.28	0.03	0.51	0.01	wgt
1	4	PAD	0.17	0.16	0.38	0.02	0.68	0.01	wgt
1	4	Pulmonary Hypertension	0.06	0.05	0.22	0.05	0.31	0.01	wgt
1	4	IABP Preop	0.02	0.02	0.12	0.02	0.71	0	wgt
1	4	LVEF	52.98	52.89	11.38	0.01	0.82	0.01	wgt
1	4	LMCA Stenosis	0.28	0.33	0.47	0.1	0	0.05	wgt
1	4	2-Vessel Disease	0.32	0.34	0.47	0.04	0.26	0.02	wgt
1	4	Previous PTCA	0.28	0.33	0.46	0.11	0	0.05	wgt
1	4	Antiplatelet Drugs	0.31	0.35	0.46	0.08	0.04	0.04	wgt
1	4	Surgery	0.08	0.09	0.28	0.06	0.18	0.02	wgt
1	4	REDO	0.04	0.03	0.19	0.04	0.34	0.01	wgt
1	4	NYHA ≥ 3	0.16	0.13	0.36	0.07	0.08	0.02	wgt
1	4	≥ 3-Vessel Disease	0.56	0.63	0.49	0.15	0	0.07	wgt
1	4	LAD Stenosis	0.96	0.95	0.24	0.02	0.62	0	wgt
2	3	Age	67.33	66.73	9.53	0.06	0.43	0.05	wgt
2	3	Gender(Male)	0.83	0.8	0.4	0.08	0.13	0.03	wgt
2	3	Hypertension	0.67	0.58	0.49	0.19	0.03	0.09	wgt
2	3	Smoker	0.41	0.37	0.48	0.08	0.23	0.04	wgt

2	3	Hypercholesterolemia	0.51	0.43	0.5	0.15	0.04	0.08	wgt
2	3	Diabetes	0.26	0.24	0.43	0.04	0.48	0.02	wgt
2	3	Insulin Dependent Diabetes	0.1	0.08	0.29	0.07	0.12	0.02	wgt
2	3	COPD	0.06	0.07	0.25	0.05	0.56	0.01	wgt
2	3	AKD	0.03	0.04	0.2	0.02	0.85	0	wgt
2	3	Dialysis	0	0.01	0.09	0.04	0.12	0	wgt
2	3	Stroke History	0.01	0.03	0.17	0.12	0	0.02	wgt
2	3	TIA History	0.01	0.03	0.17	0.11	0	0.02	wgt
2	3	Carotid Stenosis > 50%	0.03	0.09	0.28	0.19	0	0.05	wgt
2	3	PAD	0.19	0.16	0.38	0.06	0.34	0.02	wgt
2	3	Pulmonary Hypertension	0.03	0.05	0.22	0.11	0.05	0.02	wgt
2	3	IABP Preop	0.01	0.01	0.12	0.03	0.28	0	wgt
2	3	LVEF	52.47	52.58	11.38	0.01	0.89	0.06	wgt
2	3	LMCA Stenosis	0.37	0.32	0.47	0.09	0.29	0.04	wgt
2	3	2-Vessel Disease	0.32	0.36	0.47	0.09	0.15	0.04	wgt
2	3	Previuos PTCA	0.26	0.28	0.46	0.03	0.64	0.01	wgt
2	3	Antiplatelet Drugs	0.34	0.32	0.46	0.04	0.6	0.02	wgt
2	3	Surgery	0.06	0.08	0.28	0.1	0.01	0.03	wgt
2	3	REDO	0	0.04	0.19	0.19	0	0.04	wgt
2	3	NYHA ≥ 3	0.17	0.13	0.36	0.12	0.07	0.04	wgt
2	3	≥ 3-Vessel Disease	0.57	0.58	0.49	0.01	0.91	0	wgt
2	3	LAD Stenosis	0.93	0.94	0.24	0.07	0.75	0.02	wgt
2	4	Age	67.33	66.97	9.53	0.04	0.64	0.04	wgt
2	4	Gender(Male)	0.83	0.8	0.4	0.08	0.16	0.03	wgt
2	4	Hypertension	0.67	0.56	0.49	0.17	0.01	0.11	wgt
2	4	Smoker	0.41	0.33	0.48	0.17	0.01	0.08	wgt
2	4	Hypercholesterolemia	0.51	0.41	0.5	0.18	0.01	0.1	wgt
2	4	Diabetes	0.26	0.25	0.43	0.03	0.62	0.01	wgt
2	4	Insulin Dependent Diabetes	0.1	0.09	0.29	0.06	0.27	0.02	wgt
2	4	COPD	0.06	0.07	0.25	0.03	0.75	0.01	wgt
2	4	AKD	0.03	0.04	0.2	0.02	0.85	0	wgt
2	4	Dialysis	0	0.01	0.09	0.02	0.28	0	wgt

2	4	Stroke History	0.01	0.02	0.17	0.08	0.08	0.01	wgt
2	4	TIA History	0.01	0.03	0.17	0.11	0	0.02	wgt
2	4	Carotid Stenosis > 50%	0.03	0.09	0.28	0.19	0	0.05	wgt
2	4	PAD	0.19	0.16	0.38	0.07	0.3	0.03	wgt
2	4	Pulmonary Hypertension	0.03	0.05	0.22	0.11	0.1	0.02	wgt
2	4	IABP Preop	0.01	0.02	0.12	0.03	0.38	0	wgt
2	4	LVEF	52.47	52.89	11.38	0.04	0.58	0.07	wgt
2	4	LMCA Stenosis	0.37	0.33	0.47	0.07	0.43	0.03	wgt
2	4	2-Vessel Disease	0.32	0.34	0.47	0.05	0.39	0.02	wgt
2	4	Previous PTCA	0.26	0.33	0.46	0.14	0.02	0.07	wgt
2	4	Antiplatelet Drugs	0.34	0.35	0.46	0.01	0.88	0	wgt
2	4	Surgery	0.06	0.09	0.28	0.14	0	0.04	wgt
2	4	REDO	0	0.03	0.19	0.16	0	0.03	wgt
2	4	NYHA ≥ 3	0.17	0.13	0.36	0.11	0.08	0.04	wgt
2	4	≥ 3-Vessel Disease	0.57	0.63	0.49	0.12	0.13	0.06	wgt
2	4	LAD Stenosis	0.93	0.95	0.24	0.11	0.61	0.03	wgt
3	4	Age	66.73	66.97	9.53	0.03	0.37	0.03	wgt
3	4	Gender(Male)	0.8	0.8	0.4	0	0.87	0	wgt
3	4	Hypertension	0.58	0.56	0.49	0.04	0.25	0.02	wgt
3	4	Smoker	0.37	0.33	0.48	0.09	0	0.04	wgt
3	4	Hypercholesterolemia	0.43	0.41	0.5	0.05	0.1	0.02	wgt
3	4	Diabetes	0.24	0.25	0.43	0.01	0.63	0.01	wgt
3	4	Insulin Dependent Diabetes	0.08	0.09	0.29	0.01	0.83	0	wgt
3	4	COPD	0.07	0.07	0.25	0.02	0.35	0.01	wgt
3	4	AKD	0.04	0.04	0.2	0	0.98	0	wgt
3	4	Dialysis	0.01	0.01	0.09	0.02	0.47	0	wgt
3	4	Stroke History	0.03	0.02	0.17	0.04	0.15	0.01	wgt
3	4	TIA History	0.03	0.03	0.17	0.01	0.78	0	wgt
3	4	Carotid Stenosis > 50%	0.09	0.09	0.28	0	0.92	0	wgt
3	4	PAD	0.16	0.16	0.38	0.01	0.78	0	wgt
3	4	Pulmonary Hypertension	0.05	0.05	0.22	0.01	0.87	0	wgt
3	4	IABP Preop	0.01	0.02	0.12	0.06	0.06	0.01	wgt

3	4	LVEF	52.58	52.89	11.38	0.03	0.32	0.03	wgt
3	4	LMCA Stenosis	0.32	0.33	0.47	0.02	0.47	0.01	wgt
3	4	2-Vessel Disease	0.36	0.34	0.47	0.04	0.22	0.02	wgt
3	4	Previous PTCA	0.28	0.33	0.46	0.11	0	0.05	wgt
3	4	Antiplatelet Drugs	0.32	0.35	0.46	0.05	0.16	0.02	wgt
3	4	Surgery	0.08	0.09	0.28	0.04	0.2	0.01	wgt
3	4	REDO	0.04	0.03	0.19	0.03	0.17	0.01	wgt
3	4	NYHA ≥ 3	0.13	0.13	0.36	0.01	0.78	0	wgt
3	4	≥ 3-Vessel Disease	0.58	0.63	0.49	0.11	0	0.06	wgt
3	4	LAD Stenosis	0.94	0.95	0.24	0.04	0.04	0.01	wgt

Abbreviations: tmt1: Treatment 1, tmt2: Treatmen 2, var: Variables, pop.sd: Standard Deviation Beetwen Treatments, Std.eff.sz: Standardized Effect Size, ks: Kolmogorov–Smirnov test , COPD: Chronic Obstructive Pulmonary Disease; AKD: Acute Kidney Disease; TIA: Transient Ischemic Attack; PAD: Peripheral Arterial Disease; IABP: Intra-Aortic Balloon Pump; NYHA: New York Heart Association Score; LVEF: Left Ventricular Ejection Fraction; LMCA: Left Main Coronary Artery; PTCA: Percutaneous Transluminal Coronary Angioplasty; unw: unweighted; wgt: weighted.

Table S13. Balance Model 3 by Cardiopulmonary Bypass

var	tx.mn	tx.sd	ct.mn	ct.sd	std.eff.sz	stat	p	ks	Stop Method
Age	67.02	9.03	65.97	9.51	0.12	6.53	0	0.05	unw
Gender(Male)	0.84	0.41	0.78	0.42	0.04	2.84	0.01	0.02	unw
Hypertension	0.63	0.48	0.56	0.49	0.13	7.74	0	0.06	unw
Smoker	0.36	0.49	0.37	0.49	0.02	1.31	0.23	0.01	unw
Hypercholesterolemia	0.45	0.51	0.43	0.5	0.13	8.69	0	0.07	unw
Diabetes	0.27	0.46	0.18	0.41	0.23	14.56	0	0.11	unw
Insulin Dependent Diabetes	0.08	0.28	0.08	0.28	0	0.36	0.79	0	unw
COPD	0.05	0.23	0.07	0.26	0.09	-5.04	0	0.02	unw
AKD	0.05	0.22	0.05	0.22	0.01	-0.76	0.51	0	unw
Dialysis	0.02	0.08	0.02	0.08	0.01	-0.79	0.44	0	unw
Stroke History	0.03	0.13	0.06	0.22	0.18	-11.23	0	0.03	unw
TIA History	0.03	0.18	0.03	0.18	0	0.08	0.98	0	unw
Carotid Stenosis > 50%	0.03	0.27	0.14	0.37	0.37	-22.05	0	0.12	unw
PAD	0.18	0.36	0.19	0.37	0.03	-1.74	0.06	0.01	unw
Pulmonary Hypertension	0.04	0.19	0.08	0.26	0.17	-9.64	0	0.04	unw
IABP Preop	0.02	0.13	0.01	0.14	0.07	5.23	0	0.01	unw
LVEF	52.97	11.87	52.35	11.45	0.03	2.43	0.03	0.07	unw
LMCA Stenosis	0.35	0.45	0.35	0.45	0	0.34	0.69	0	unw
2-Vessel Disease	0.32	0.47	0.37	0.49	0.12	-6.54	0	0.05	unw
Previous PTCA	0.39	0.61	0.15	0.36	0.81	41.58	0	0.29	unw
Antiplatelet Drugs	0.33	0.48	0.32	0.47	0.03	2.01	0.06	0.01	unw
Surgery	0.07	0.25	0.13	0.35	0.24	-13.01	0	0.07	unw
REDO	0.02	0.16	0.07	0.26	0.25	-12.05	0	0.05	unw
NYHA ≥ 3	0.21	0.41	0.12	0.32	0.29	18.8	0	0.13	unw
≥ 3-Vessel Disease	0.71	0.44	0.54	0.63	0.43	25.62	0	0.21	unw
LAD Stenosis	0.95	0.18	0.96	0.33	0.32	14.02	0	0.07	unw
Age	67.05	9.38	66.79	9.4	0.03	1.36	0.19	0.01	wgt
Gender(Male)	0.82	0.41	0.81	0.42	0.02	1.01	0.33	0.02	wgt
Hypertension	0.58	0.48	0.57	0.48	0.02	0.92	0.36	0.01	wgt
Smoker	0.37	0.47	0.38	0.47	0.01	-0.56	0.56	0.01	wgt

Hypercholesterolemia	0.45	0.52	0.45	0.52	0.01	-0.32	0.77	0	wgt
Diabetes	0.26	0.45	0.25	0.44	0.04	0.68	0.08	0.02	wgt
Insulin Dependent Diabetes	0.11	0.28	0.1	0.27	0.03	1.07	0.29	0.01	wgt
COPD	0.05	0.26	0.06	0.27	0.02	-1.12	0.27	0.01	wgt
AKD	0.05	0.18	0.05	0.19	0	-0.11	0.89	0	wgt
Dialysis	0.02	0.08	0.02	0.08	0.01	0.28	0.81	0	wgt
Stroke History	0.03	0.16	0.04	0.18	0.05	-2.54	0.01	0.01	wgt
TIA History	0.02	0.16	0.02	0.15	0.02	0.66	0.58	0	wgt
Carotid Stenosis > 50%	0.08	0.27	0.1	0.3	0.06	-2.69	0.01	0.02	wgt
PAD	0.16	0.37	0.16	0.36	0.02	0.85	0.46	0.01	wgt
Pulmonary Hypertension	0.06	0.23	0.06	0.24	0.02	-0.67	0.58	0	wgt
IABP Preop	0.03	0.13	0.02	0.12	0.03	1.59	0.15	0	wgt
LVEF	53.1	11.05	52.79	11.01	0.01	0.49	0.68	0.01	wgt
LMCA Stenosis	0.34	0.46	0.33	0.46	0.02	0.75	0.51	0.01	wgt
2-Vessel Disease	0.35	0.48	0.35	0.48	0.01	-0.54	0.61	0.01	wgt
Previous PTCA	0.33	0.46	0.28	0.47	0.09	3.54	0	0.05	wgt
Antiplatelet Drugs	0.32	0.46	0.33	0.46	0.02	0.97	0.41	0.01	wgt
Surgery	0.07	0.26	0.08	0.27	0.03	-1.58	0.16	0.01	wgt
REDO	0.04	0.18	0.05	0.21	0.06	-2.54	0.01	0.01	wgt
NYHA ≥ 3	0.14	0.35	0.12	0.31	0.08	3.68	0	0.02	wgt
≥ 3-Vessel Disease	0.58	0.48	0.57	0.48	0.05	2.36	0.02	0.02	wgt
LAD Stenosis	0.94	0.21	0.92	0.24	0.06	2.24	0.01	0.01	wgt

Abbreviations: tmt1: Treatment 1, tmt2: Treatmen 2, var: Variables, pop.sd: Standard Deviation Beetwen Treatments, Std.eff.sz: Standardized Effect Size, ks: Kolmogorov–Smirnov test , COPD: Chronic Obstructive Pulmonary Disease; AKD: Acute Kidney Disease; TIA: Transient Ischemic Attack; PAD: Peripheral Arterial Disease; IABP: Intra-Aortic Balloon Pump; NYHA: New York Heart Association Score; LVEF: Left Ventricular Ejection Fraction; LMCA: Left Main Coronary Artery; PTCA: Percutaneous Transluminal Coronary Angioplasty; unw: unweighted; wgt: weighted.

Table S14. Balance Table Model 4 by Total Aortic Clamp

var	tx.mn	tx.sd	ct.mn	ct.sd	std.eff.sz	stat	p	ks	Stop Method
Age	66.77	9.47	67.15	9.65	0.04	-2.38	0.02	0.02	unw
Gender(Male)	0.79	0.41	0.82	0.39	0.06	-3.7	0	0.02	unw
Hypertension	0.64	0.48	0.47	0.5	0.35	20.82	0	0.17	unw
Smoker	0.42	0.49	0.25	0.43	0.36	22.67	0	0.17	unw
Hypercholesterolemia	0.5	0.5	0.32	0.47	0.37	23.06	0	0.18	unw
Diabetes	0.24	0.43	0.26	0.44	0.03	-1.68	0.09	0.01	unw
Insulin Dependent Diabetes	0.1	0.3	0.08	0.28	0.05	2.93	0	0.01	unw
COPD	0.08	0.27	0.05	0.21	0.12	7.75	0	0.03	unw
AKD	0.05	0.22	0.03	0.16	0.12	7.8	0	0.02	unw
Dialysis	0.01	0.1	0.01	0.08	0.03	1.93	0.05	0	unw
Stroke History	0.04	0.19	0.01	0.1	0.16	11.51	0	0.03	unw
TIA History	0.04	0.19	0.01	0.12	0.14	10	0	0.02	unw
Carotid Stenosis > 50%	0.1	0.3	0.06	0.23	0.16	10.24	0	0.04	unw
PAD	0.2	0.4	0.12	0.32	0.21	13.21	0	0.08	unw
Pulmonary Hypertension	0.06	0.24	0.02	0.15	0.19	13.23	0	0.04	unw
IABP Preop	0.01	0.11	0.02	0.15	0.07	-3.92	0	0.01	unw
LVEF	51.58	11.75	54.46	10.31	0.25	-15.9	0	0.16	unw
LMCA Stenosis	0.34	0.47	0.3	0.46	0.07	4.31	0	0.03	unw
2-Vessel Disease	0.33	0.47	0.35	0.48	0.03	-2.01	0.04	0.02	unw
Previous PTCA	0.37	0.48	0.19	0.39	0.4	25.8	0	0.18	unw
Antiplatelet Drugs	0.33	0.47	0.29	0.45	0.08	4.73	0	0.04	unw
Surgery	0.11	0.31	0.05	0.21	0.2	13.89	0	0.06	unw
REDO	0.05	0.21	0.02	0.14	0.13	9.02	0	0.03	unw
NYHA ≥ 3	0.16	0.36	0.15	0.35	0.03	1.62	0.11	0.01	unw
≥ 3-Vessel Disease	0.64	0.48	0.53	0.5	0.24	13.95	0	0.12	unw
LAD Stenosis	0.93	0.25	0.94	0.23	0.04	-2.45	0.01	0.01	unw
Age	66.79	9.51	66.79	9.49	0	0.05	0.96	0.01	wgt
Gender(Male)	0.8	0.4	0.81	0.39	0.03	-1.59	0.11	0.01	wgt
Hypertension	0.59	0.49	0.58	0.49	0.02	1.14	0.25	0.01	wgt
Smoker	0.37	0.48	0.35	0.48	0.03	1.51	0.13	0.02	wgt

Hypercholesterolemia	0.45	0.5	0.43	0.5	0.03	1.28	0.2	0.01	wgt
Diabetes	0.25	0.43	0.26	0.44	0.03	-1.23	0.22	0.01	wgt
Insulin Dependent Diabetes	0.09	0.29	0.09	0.29	0.01	0.43	0.67	0	wgt
COPD	0.07	0.25	0.07	0.25	0.02	0.71	0.48	0	wgt
AKD	0.04	0.21	0.04	0.2	0.02	0.85	0.4	0	wgt
Dialysis	0.01	0.09	0.01	0.09	0	-0.03	0.98	0	wgt
Stroke History	0.03	0.17	0.03	0.16	0.02	0.72	0.47	0	wgt
TIA History	0.03	0.18	0.03	0.16	0.02	0.88	0.38	0	wgt
Carotid Stenosis > 50%	0.09	0.28	0.08	0.27	0.03	1.36	0.18	0.01	wgt
PAD	0.17	0.38	0.17	0.37	0.01	0.44	0.66	0	wgt
Pulmonary Hypertension	0.05	0.22	0.05	0.22	0.01	0.25	0.8	0	wgt
IABP Preop	0.02	0.12	0.02	0.13	0.01	-0.41	0.68	0	wgt
LVEF	52.38	11.42	52.59	11.21	0.02	-0.8	0.42	0.02	wgt
LMCA Stenosis	0.33	0.47	0.31	0.46	0.04	1.85	0.06	0.02	wgt
2-Vessel Disease	0.33	0.47	0.33	0.47	0.01	0.71	0.48	0.01	wgt
Previous PTCA	0.32	0.47	0.3	0.46	0.04	1.87	0.06	0.02	wgt
Antiplatelet Drugs	0.32	0.47	0.32	0.47	0.01	-0.39	0.7	0	wgt
Surgery	0.09	0.28	0.08	0.27	0.04	1.55	0.12	0.01	wgt
REDO	0.04	0.19	0.03	0.18	0.03	1.38	0.17	0.01	wgt
NYHA ≥ 3	0.15	0.36	0.15	0.36	0.01	-0.33	0.74	0	wgt
≥ 3-Vessel Disease	0.61	0.49	0.59	0.49	0.04	1.91	0.06	0.02	wgt
LAD Stenosis	0.94	0.24	0.94	0.23	0	-0.2	0.84	0	wgt

Abbreviations: tmt1: Treatment 1, tmt2: Treatmen 2, var: Variables, pop.sd: Standard Deviation Beetwen Treatments, Std.eff.sz: Standardized Effect Size,
ks: Kolmogorov–Smirnov test , COPD: Chronic Obstructive Pulmonary Disease; AKD: Acute Kidney Disease; TIA: Transient Ischemic Attack; PAD: Peripheral Arterial Disease;
IABP: Intra-Aortic Balloon Pump; NYHA: New York Heart Association Score; LVEF: Left Ventricular Ejection Fraction; LMCA: Left Main Coronary Artery;
PTCA: Percutaneous Transluminal Coronary Angioplasty; unw: unweighted; wgt: weighted.

Tab. S15. Balance Table Model 5 by Side-Biting Clamp

var	tx.mn	tx.sd	ct.mn	ct.sd	std.eff.sz	stat	p	ks	Stop Method
Age	67.33	9.44	66.28	9.62	0.11	6.87	0	0.05	unw
Gender(Male)	0.81	0.39	0.79	0.41	0.04	2.79	0.01	0.02	unw
Hypertension	0.61	0.49	0.55	0.5	0.12	7.63	0	0.06	unw
Smoker	0.37	0.48	0.36	0.48	0.02	1.26	0.21	0.01	unw
Hypercholesterolemia	0.47	0.5	0.4	0.49	0.14	8.54	0	0.07	unw
Diabetes	0.29	0.45	0.19	0.39	0.22	14.39	0	0.1	unw
Insulin Dependent Diabetes	0.09	0.29	0.09	0.29	0	0.3	0.77	0	unw
COPD	0.06	0.24	0.08	0.27	0.09	-5.25	0	0.02	unw
AKD	0.04	0.2	0.04	0.21	0.01	-0.7	0.49	0	unw
Dialysis	0.01	0.09	0.01	0.1	0.01	-0.81	0.42	0	unw
Stroke History	0.02	0.12	0.05	0.21	0.19	-11.07	0	0.03	unw
TIA History	0.03	0.17	0.03	0.17	0	0.09	0.93	0	unw
Carotid Stenosis > 50%	0.04	0.2	0.15	0.35	0.37	-21.91	0	0.1	unw
PAD	0.17	0.37	0.18	0.38	0.03	-1.84	0.07	0.01	unw
Pulmonary Hypertension	0.03	0.18	0.07	0.25	0.16	-9.82	0	0.04	unw
IABP Preop	0.02	0.14	0.01	0.1	0.08	5.05	0	0.01	unw
LVEF	52.68	11.48	52.29	11.23	0.03	2.17	0.03	0.07	unw
LMCA Stenosis	0.33	0.47	0.33	0.47	0	0.3	0.77	0	unw
2-Vessel Disease	0.31	0.46	0.36	0.48	0.11	-6.6	0	0.05	unw
Previous PTCA	0.43	0.5	0.15	0.36	0.6	41.26	0	0.28	unw
Antiplatelet Drugs	0.32	0.47	0.31	0.46	0.03	1.88	0.06	0.01	unw
Surgery	0.06	0.24	0.12	0.33	0.21	-12.95	0	0.06	unw
REDO	0.02	0.15	0.06	0.24	0.2	-11.79	0	0.04	unw
NYHA ≥ 3	0.19	0.4	0.1	0.29	0.27	18.1	0	0.1	unw
≥ 3-Vessel Disease	0.68	0.47	0.5	0.5	0.38	24.09	0	0.19	unw
LAD Stenosis	0.96	0.19	0.9	0.29	0.23	13.79	0	0.06	unw
Age	67	9.39	66.73	9.41	0.03	1.35	0.18	0.02	wgt
Gender(Male)	0.8	0.4	0.79	0.41	0.02	0.99	0.32	0.01	wgt
Hypertension	0.59	0.49	0.58	0.49	0.02	0.94	0.34	0.01	wgt
Smoker	0.36	0.48	0.37	0.48	0.01	-0.57	0.57	0.01	wgt

Hypercholesterolemia	0.44	0.5	0.44	0.5	0.01	-0.31	0.75	0	wgt
Diabetes	0.25	0.44	0.24	0.43	0.04	1.67	0.09	0.02	wgt
Insulin Dependent Diabetes	0.1	0.29	0.09	0.28	0.03	1.08	0.28	0.01	wgt
COPD	0.06	0.25	0.07	0.26	0.02	-1.13	0.26	0.01	wgt
AKD	0.04	0.19	0.04	0.2	0	-0.12	0.9	0	wgt
Dialysis	0.01	0.09	0.01	0.09	0.01	0.29	0.77	0	wgt
Stroke History	0.02	0.15	0.03	0.17	0.05	-2.51	0.01	0.01	wgt
TIA History	0.03	0.17	0.03	0.16	0.02	0.64	0.52	0	wgt
Carotid Stenosis > 50%	0.07	0.26	0.09	0.29	0.06	-2.73	0.01	0.02	wgt
PAD	0.17	0.38	0.17	0.37	0.02	0.83	0.41	0.01	wgt
Pulmonary Hypertension	0.05	0.22	0.05	0.23	0.02	-0.62	0.54	0	wgt
IABP Preop	0.02	0.12	0.01	0.11	0.03	1.53	0.13	0	wgt
LVEF	52.7	11.18	52.58	10.98	0.01	0.51	0.61	0.01	wgt
LMCA Stenosis	0.33	0.47	0.32	0.47	0.02	0.7	0.48	0.01	wgt
2-Vessel Disease	0.34	0.47	0.34	0.47	0.01	-0.56	0.58	0.01	wgt
Previous PTCA	0.32	0.47	0.29	0.45	0.08	3.25	0	0.04	wgt
Antiplatelet Drugs	0.33	0.47	0.32	0.47	0.02	0.92	0.36	0.01	wgt
Surgery	0.08	0.27	0.09	0.28	0.03	-1.62	0.11	0.01	wgt
REDO	0.03	0.17	0.04	0.2	0.06	-2.72	0.01	0.01	wgt
NYHA ≥ 3	0.15	0.36	0.13	0.33	0.07	3.1	0	0.02	wgt
≥ 3-Vessel Disease	0.61	0.49	0.59	0.49	0.05	2.25	0.02	0.02	wgt
LAD Stenosis	0.95	0.22	0.93	0.25	0.06	2.65	0.01	0.01	wgt

Abbreviations: tmt1: Treatment 1, tmt2: Treatmen 2, var: Variables, pop.sd: Standard Deviation Beetwen Treatments, std.eff.sz: Std.eff.sz: Standardized Effect Size, ks: Kolmogorov–Smirnov test, COPD: Chronic Obstructive Pulmonary Disease; AKD: Acute Kidney Disease; TIA: Transient Ischemic Attack; PAD: Peripheral Arterial Disease; IABP: Intra-Aortic Balloon Pump; NYHA: New York Heart Association Score; LVEF: Left Ventricular Ejection Fraction; LMCA: Left Main Coronary Artery; PTCA: Percutaneous Transluminal Coronary Angioplasty; unw: unweighte; wgt: weighted.

Tab. S16. Balance Table Model 6 by Proximal Anastomoses

tmt1	tmt2	var	mean1	mean2	pop.sd	std.eff.sz	p	ks	Stop Method
0	1	Age	66.92	67.1	9.53	0.02	0.39	0.02	unw
0	1	Gender(Male)	0.79	0.8	0.4	0.04	0.05	0.02	unw
0	1	Hypertension	0.36	0.62	0.49	0.53	0	0.26	unw
0	1	Smoker	0.18	0.39	0.48	0.43	0	0.21	unw
0	1	Hypercholesterolemia	0.21	0.48	0.5	0.53	0	0.26	unw
0	1	Diabetes	0.2	0.27	0.43	0.16	0	0.07	unw
0	1	Insulin Dependent Diabetes	0.05	0.1	0.29	0.17	0	0.05	unw
0	1	COPD	0.07	0.07	0.25	0	1	0	unw
0	1	AKD	0.04	0.04	0.2	0	0.95	0	unw
0	1	Dialysis	0.01	0.01	0.09	0	1	0	unw
0	1	Stroke History	0.02	0.03	0.17	0.04	0.03	0.01	unw
0	1	TIA History	0.02	0.03	0.17	0.09	0	0.02	unw
0	1	Carotid Stenosis > 50%	0.07	0.07	0.28	0.01	0.67	0	unw
0	1	PAD	0.1	0.18	0.38	0.2	0	0.08	unw
0	1	Pulmonary Hypertension	0.03	0.05	0.22	0.06	0	0.01	unw
0	1	IABP Preop	0.02	0.02	0.12	0.01	0.51	0	unw
0	1	LVEF	54.76	52.17	11.38	0.23	0	0.12	unw
0	1	LMCA Stenosis	0.26	0.33	0.47	0.15	0	0.07	unw
0	1	2-Vessel Disease	0.46	0.32	0.47	0.3	0	0.14	unw
0	1	Previuos PTCA	0.13	0.37	0.46	0.53	0	0.24	unw
0	1	Antiplatelet Drugs	0.27	0.31	0.46	0.09	0	0.04	unw
0	1	Surgery	0.07	0.09	0.28	0.08	0	0.02	unw
0	1	REDO	0.04	0.03	0.19	0.05	0.03	0.01	unw
0	1	NYHA ≥ 3	0.11	0.17	0.36	0.16	0	0.06	unw
0	1	≥ 3-Vessel Disease	0.16	0.68	0.49	0.74	0	0.51	unw
0	1	LAD Stenosis	0.88	0.95	0.24	0.3	0	0.07	unw
0	2	Age	66.92	64.87	9.53	0.22	0	0.1	unw
0	2	Gender(Male)	0.79	0.79	0.4	0.01	0.85	0	unw
0	2	Hypertension	0.36	0.77	0.49	0.73	0	0.41	unw

0	2	Smoker	0.18	0.55	0.48	0.76	0	0.37	unw
0	2	Hypercholesterolemia	0.21	0.6	0.5	0.77	0	0.38	unw
0	2	Diabetes	0.2	0.14	0.43	0.13	0	0.05	unw
0	2	Insulin Dependent Diabetes	0.05	0.12	0.29	0.24	0	0.07	unw
0	2	COPD	0.07	0.06	0.25	0.02	0.47	0.01	unw
0	2	AKD	0.04	0.05	0.2	0.04	0.27	0.01	unw
0	2	Dialysis	0.01	0.01	0.09	0.02	0.57	0	unw
0	2	Stroke History	0.02	0.06	0.17	0.23	0	0.04	unw
0	2	TIA History	0.02	0.03	0.17	0.1	0	0.02	unw
0	2	Carotid Stenosis > 50%	0.07	0.24	0.28	0.6	0	0.17	unw
0	2	PAD	0.1	0.25	0.38	0.38	0	0.14	unw
0	2	Pulmonary Hypertension	0.03	0.09	0.22	0.27	0	0.06	unw
0	2	IABP Preop	0.02	0	0.12	0.1	0	0.01	unw
0	2	LVEF	54.76	50.83	11.38	0.35	0	0.26	unw
0	2	LMCA Stenosis	0.26	0.4	0.47	0.3	0	0.14	unw
0	2	2-Vessel Disease	0.46	0.21	0.47	0.52	0	0.25	unw
0	2	Previous PTCA	0.13	0.15	0.46	0.04	0.1	0.02	unw
0	2	Antiplatelet Drugs	0.27	0.46	0.46	0.41	0	0.19	unw
0	2	Surgery	0.07	0.09	0.28	0.06	0.05	0.02	unw
0	2	REDO	0.04	0.06	0.19	0.11	0.01	0.02	unw
0	2	NYHA ≥ 3	0.11	0.1	0.36	0.03	0.28	0.01	unw
0	2	≥ 3-Vessel Disease	0.16	0.93	0.49	0.79	0	0.77	unw
0	2	LAD Stenosis	0.88	0.94	0.24	0.24	0	0.06	unw
0	3	Age	66.92	62.18	9.53	0.5	0	0.25	unw
0	3	Gender(Male)	0.79	0.88	0.4	0.24	0.04	0.1	unw
0	3	Hypertension	0.36	0.9	0.49	0.73	0	0.54	unw
0	3	Smoker	0.18	0.65	0.48	0.74	0	0.46	unw
0	3	Hypercholesterolemia	0.21	0.71	0.5	0.81	0	0.49	unw
0	3	Diabetes	0.2	0.27	0.43	0.18	0.23	0.08	unw
0	3	Insulin Dependent Diabetes	0.05	0.16	0.29	0.36	0.04	0.11	unw
0	3	COPD	0.07	0.14	0.25	0.28	0.15	0.07	unw
0	3	AKD	0.04	0.08	0.2	0.18	0.34	0.04	unw

0	3	Dialysis	0.01	0.02	0.09	0.11	0.58	0.01	unw
0	3	Stroke History	0.02	0.08	0.17	0.35	0.12	0.06	unw
0	3	TIA History	0.02	0.04	0.17	0.13	0.42	0.02	unw
0	3	Carotid Stenosis > 50%	0.07	0.33	0.28	0.77	0	0.26	unw
0	3	PAD	0.1	0.22	0.38	0.3	0.05	0.11	unw
0	3	Pulmonary Hypertension	0.03	0.18	0.22	0.66	0.01	0.14	unw
0	3	IABP Preop	0.02	0	0.12	0.12	0	0.02	unw
0	3	LVEF	54.76	50.36	11.38	0.39	0.01	0.26	unw
0	3	LMCA Stenosis	0.26	0.47	0.47	0.44	0	0.21	unw
0	3	2-Vessel Disease	0.46	0.12	0.47	0.73	0	0.34	unw
0	3	Previous PTCA	0.13	0.1	0.46	0.07	0.45	0.03	unw
0	3	Antiplatelet Drugs	0.27	0.41	0.46	0.31	0.04	0.15	unw
0	3	Surgery	0.07	0	0.28	0.24	0	0.07	unw
0	3	REDO	0.04	0.04	0.19	0.02	0.9	0	unw
0	3	NYHA ≥ 3	0.11	0.16	0.36	0.13	0.36	0.05	unw
0	3	≥ 3-Vessel Disease	0.16	1	0.49	0.79	0	0.84	unw
0	3	LAD Stenosis	0.88	0.94	0.24	0.26	0.06	0.06	unw
1	2	Age	67.1	64.87	9.53	0.23	0	0.09	unw
1	2	Gender(Male)	0.8	0.79	0.4	0.03	0.26	0.01	unw
1	2	Hypertension	0.62	0.77	0.49	0.31	0	0.16	unw
1	2	Smoker	0.39	0.55	0.48	0.33	0	0.16	unw
1	2	Hypercholesterolemia	0.48	0.6	0.5	0.24	0	0.12	unw
1	2	Diabetes	0.27	0.14	0.43	0.29	0	0.13	unw
1	2	Insulin Dependent Diabetes	0.1	0.12	0.29	0.07	0.04	0.02	unw
1	2	COPD	0.07	0.06	0.25	0.02	0.41	0.01	unw
1	2	AKD	0.04	0.05	0.2	0.04	0.23	0.01	unw
1	2	Dialysis	0.01	0.01	0.09	0.02	0.53	0	unw
1	2	Stroke History	0.03	0.06	0.17	0.19	0	0.03	unw
1	2	TIA History	0.03	0.03	0.17	0	0.88	0	unw
1	2	Carotid Stenosis > 50%	0.07	0.24	0.28	0.61	0	0.17	unw
1	2	PAD	0.18	0.25	0.38	0.18	0	0.07	unw
1	2	Pulmonary Hypertension	0.05	0.09	0.22	0.21	0	0.04	unw

1	2	IABP Preop	0.02	0	0.12	0.11	0	0.01	unw
1	2	LVEF	52.17	50.83	11.38	0.12	0	0.16	unw
1	2	LMCA Stenosis	0.33	0.4	0.47	0.15	0	0.07	unw
1	2	2-Vessel Disease	0.32	0.21	0.47	0.22	0	0.1	unw
1	2	Previuos PTCA	0.37	0.15	0.46	0.48	0	0.22	unw
1	2	Antiplatelet Drugs	0.31	0.46	0.46	0.32	0	0.15	unw
1	2	Surgery	0.09	0.09	0.28	0.02	0.53	0.01	unw
1	2	REDO	0.03	0.06	0.19	0.16	0	0.03	unw
1	2	NYHA ≥ 3	0.17	0.1	0.36	0.19	0	0.07	unw
1	2	≥ 3-Vessel Disease	0.68	0.93	0.49	0.53	0	0.26	unw
1	2	LAD Stenosis	0.95	0.94	0.24	0.06	0.05	0.01	unw
1	3	Age	67.1	62.18	9.53	0.52	0	0.26	unw
1	3	Gender(Male)	0.8	0.88	0.4	0.2	0.08	0.08	unw
1	3	Hypertension	0.62	0.9	0.49	0.58	0	0.28	unw
1	3	Smoker	0.39	0.65	0.48	0.53	0	0.26	unw
1	3	Hypercholesterolemia	0.48	0.71	0.5	0.46	0	0.23	unw
1	3	Diabetes	0.27	0.27	0.43	0.01	0.93	0.01	unw
1	3	Insulin Dependent Diabetes	0.1	0.16	0.29	0.2	0.27	0.06	unw
1	3	COPD	0.07	0.14	0.25	0.28	0.15	0.07	unw
1	3	AKD	0.04	0.08	0.2	0.18	0.34	0.04	unw
1	3	Dialysis	0.01	0.02	0.09	0.11	0.58	0.01	unw
1	3	Stroke History	0.03	0.08	0.17	0.31	0.17	0.05	unw
1	3	TIA History	0.03	0.04	0.17	0.04	0.82	0.01	unw
1	3	Carotid Stenosis > 50%	0.07	0.33	0.28	0.83	0	0.26	unw
1	3	PAD	0.18	0.22	0.38	0.1	0.52	0.04	unw
1	3	Pulmonary Hypertension	0.05	0.18	0.22	0.6	0.02	0.13	unw
1	3	IABP Preop	0.02	0	0.12	0.14	0	0.02	unw
1	3	LVEF	52.17	50.36	11.38	0.16	0.31	0.16	unw
1	3	LMCA Stenosis	0.33	0.47	0.47	0.29	0.05	0.14	unw
1	3	2-Vessel Disease	0.32	0.12	0.47	0.43	0	0.2	unw
1	3	Previous PTCA	0.37	0.1	0.46	0.59	0	0.28	unw
1	3	Antiplatelet Drugs	0.31	0.41	0.46	0.22	0.14	0.1	unw

1	3	Surgery	0.09	0	0.28	0.32	0	0.09	unw
1	3	REDO	0.03	0.04	0.19	0.03	0.84	0.01	unw
1	3	NYHA ≥ 3	0.17	0.16	0.36	0.03	0.83	0.01	unw
1	3	≥ 3-Vessel Disease	0.68	1	0.49	0.66	0	0.32	unw
1	3	LAD Stenosis	0.95	0.94	0.24	0.04	0.77	0.01	unw
2	3	Age	64.87	62.18	9.53	0.28	0.02	0.18	unw
2	3	Gender(Male)	0.79	0.88	0.4	0.23	0.05	0.09	unw
2	3	Hypertension	0.77	0.9	0.49	0.26	0	0.13	unw
2	3	Smoker	0.55	0.65	0.48	0.2	0.15	0.1	unw
2	3	Hypercholesterolemia	0.6	0.71	0.5	0.22	0.1	0.11	unw
2	3	Diabetes	0.14	0.27	0.43	0.3	0.04	0.13	unw
2	3	Insulin Dependent Diabetes	0.12	0.16	0.29	0.12	0.48	0.04	unw
2	3	COPD	0.06	0.14	0.25	0.3	0.12	0.08	unw
2	3	AKD	0.05	0.08	0.2	0.14	0.46	0.03	unw
2	3	Dialysis	0.01	0.02	0.09	0.09	0.65	0.01	unw
2	3	Stroke History	0.06	0.08	0.17	0.11	0.62	0.02	unw
2	3	TIA History	0.03	0.04	0.17	0.03	0.85	0.01	unw
2	3	Carotid Stenosis > 50%	0.24	0.33	0.28	0.32	0.17	0.09	unw
2	3	PAD	0.25	0.22	0.38	0.08	0.61	0.03	unw
2	3	Pulmonary Hypertension	0.09	0.18	0.22	0.39	0.12	0.08	unw
2	3	IABP Preop	0	0	0.12	0.03	0.05	0	unw
2	3	LVEF	50.83	50.36	11.38	0.04	0.8	0.09	unw
2	3	LMCA Stenosis	0.4	0.47	0.47	0.14	0.35	0.07	unw
2	3	2-Vessel Disease	0.21	0.12	0.47	0.21	0.04	0.1	unw
2	3	Previous PTCA	0.15	0.1	0.46	0.11	0.23	0.05	unw
2	3	Antiplatelet Drugs	0.46	0.41	0.46	0.1	0.52	0.05	unw
2	3	Surgery	0.09	0	0.28	0.31	0	0.09	unw
2	3	REDO	0.06	0.04	0.19	0.13	0.37	0.03	unw
2	3	NYHA ≥ 3	0.1	0.16	0.36	0.16	0.26	0.06	unw
2	3	≥ 3-Vessel Disease	0.93	1	0.49	0.14	0	0.07	unw
2	3	LAD Stenosis	0.94	0.94	0.24	0.02	0.89	0	unw
0	1	Age	66.8	66.91	9.53	0.01	0.73	0.03	wgt

0	1	Gender(Male)	0.8	0.8	0.4	0	0.94	0	wgt
0	1	Hypertension	0.58	0.59	0.49	0.02	0.48	0.01	wgt
0	1	Smoker	0.37	0.37	0.48	0.01	0.78	0	wgt
0	1	Hypercholesterolemia	0.44	0.45	0.5	0.02	0.64	0.01	wgt
0	1	Diabetes	0.25	0.25	0.43	0	0.93	0	wgt
0	1	Insulin Dependent Diabetes	0.08	0.1	0.29	0.04	0.23	0.01	wgt
0	1	COPD	0.09	0.07	0.25	0.07	0.06	0.02	wgt
0	1	AKD	0.04	0.04	0.2	0	0.99	0	wgt
0	1	Dialysis	0.01	0.01	0.09	0.01	0.75	0	wgt
0	1	Stroke History	0.04	0.03	0.17	0.07	0.12	0.01	wgt
0	1	TIA History	0.03	0.03	0.17	0.02	0.53	0	wgt
0	1	Carotid Stenosis > 50%	0.11	0.09	0.28	0.07	0.07	0.02	wgt
0	1	PAD	0.17	0.17	0.38	0.01	0.76	0	wgt
0	1	Pulmonary Hypertension	0.07	0.05	0.22	0.07	0.1	0.02	wgt
0	1	IABP Preop	0.02	0.02	0.12	0	1	0	wgt
0	1	LVEF	52.6	52.42	11.38	0.02	0.63	0.02	wgt
0	1	LMCA Stenosis	0.31	0.33	0.47	0.04	0.23	0.02	wgt
0	1	2-Vessel Disease	0.36	0.34	0.47	0.05	0.11	0.02	wgt
0	1	Previous PTCA	0.27	0.32	0.46	0.11	0	0.05	wgt
0	1	Antiplatelet Drugs	0.3	0.32	0.46	0.03	0.43	0.01	wgt
0	1	Surgery	0.08	0.09	0.28	0.01	0.71	0	wgt
0	1	REDO	0.06	0.04	0.19	0.1	0.03	0.02	wgt
0	1	NYHA ≥ 3	0.12	0.15	0.36	0.08	0.02	0.03	wgt
0	1	≥ 3-Vessel Disease	0.57	0.62	0.49	0.1	0	0.05	wgt
0	1	LAD Stenosis	0.93	0.94	0.24	0.04	0.16	0.01	wgt
0	2	Age	66.8	66.2	9.53	0.06	0.29	0.07	wgt
0	2	Gender(Male)	0.8	0.79	0.4	0.04	0.64	0.01	wgt
0	2	Hypertension	0.58	0.68	0.49	0.19	0	0.09	wgt
0	2	Smoker	0.37	0.46	0.48	0.19	0	0.09	wgt
0	2	Hypercholesterolemia	0.44	0.54	0.5	0.18	0	0.1	wgt
0	2	Diabetes	0.25	0.24	0.43	0.03	0.69	0.01	wgt
0	2	Insulin Dependent Diabetes	0.08	0.09	0.29	0.04	0.52	0.01	wgt

0	2	COPD	0.09	0.07	0.25	0.08	0.2	0.02	wgt
0	2	AKD	0.04	0.03	0.2	0.04	0.34	0.01	wgt
0	2	Dialysis	0.01	0	0.09	0.05	0.08	0.01	wgt
0	2	Stroke History	0.04	0.05	0.17	0.03	0.68	0.01	wgt
0	2	TIA History	0.03	0.02	0.17	0.07	0.1	0.01	wgt
0	2	Carotid Stenosis > 50%	0.11	0.13	0.28	0.07	0.3	0.02	wgt
0	2	PAD	0.17	0.2	0.38	0.08	0.21	0.03	wgt
0	2	Pulmonary Hypertension	0.07	0.05	0.22	0.06	0.28	0.01	wgt
0	2	IABP Preop	0.02	0	0.12	0.11	0	0.01	wgt
0	2	LVEF	52.6	51.69	11.38	0.08	0.21	0.1	wgt
0	2	LMCA Stenosis	0.31	0.35	0.47	0.08	0.24	0.04	wgt
0	2	2-Vessel Disease	0.36	0.29	0.47	0.15	0.03	0.07	wgt
0	2	Previuos PTCA	0.27	0.28	0.46	0.02	0.82	0.01	wgt
0	2	Antiplatelet Drugs	0.3	0.32	0.46	0.04	0.55	0.02	wgt
0	2	Surgery	0.08	0.1	0.28	0.05	0.45	0.01	wgt
0	2	REDO	0.06	0.06	0.19	0.02	0.8	0	wgt
0	2	NYHA ≥ 3	0.12	0.11	0.36	0.03	0.61	0.01	wgt
0	2	≥ 3-Vessel Disease	0.57	0.7	0.49	0.17	0	0.13	wgt
0	2	LAD Stenosis	0.93	0.95	0.24	0.08	0.06	0.02	wgt
0	3	Age	66.8	65.68	9.53	0.12	0.75	0.11	wgt
0	3	Gender(Male)	0.8	0.93	0.4	0.14	0	0.13	wgt
0	3	Hypertension	0.58	0.86	0.49	0.15	0	0.27	wgt
0	3	Smoker	0.37	0.65	0.48	0.19	0.03	0.29	wgt
0	3	Hypercholesterolemia	0.44	0.79	0.5	0.11	0	0.35	wgt
0	3	Diabetes	0.25	0.29	0.43	0.09	0.75	0.04	wgt
0	3	Insulin Dependent Diabetes	0.08	0.2	0.29	0.18	0.29	0.11	wgt
0	3	COPD	0.09	0.01	0.25	0.19	0	0.07	wgt
0	3	AKD	0.04	0.04	0.2	0	0.98	0	wgt
0	3	Dialysis	0.01	0	0.09	0.06	0.25	0.01	wgt
0	3	Stroke History	0.04	0.03	0.17	0.07	0.57	0.01	wgt
0	3	TIA History	0.03	0	0.17	0.13	0	0.02	wgt
0	3	Carotid Stenosis > 50%	0.11	0.11	0.28	0.03	0.9	0.01	wgt

0	3	PAD	0.17	0.15	0.38	0.04	0.82	0.02	wgt
0	3	Pulmonary Hypertension	0.07	0.05	0.22	0.1	0.5	0.02	wgt
0	3	IABP Preop	0.02	0	0.12	0.12	0	0.02	wgt
0	3	LVEF	52.6	51.14	11.38	0.13	0.28	0.29	wgt
0	3	LMCA Stenosis	0.31	0.26	0.47	0.12	0.58	0.06	wgt
0	3	2-Vessel Disease	0.36	0.09	0.47	0.19	0	0.28	wgt
0	3	Previous PTCA	0.27	0.41	0.46	0.15	0.41	0.14	wgt
0	3	Antiplatelet Drugs	0.3	0.24	0.46	0.14	0.47	0.06	wgt
0	3	Surgery	0.08	0	0.28	0.15	0	0.08	wgt
0	3	REDO	0.06	0	0.19	0.17	0	0.05	wgt
0	3	NYHA ≥ 3	0.12	0.09	0.36	0.08	0.59	0.03	wgt
0	3	≥ 3-Vessel Disease	0.57	1	0.49	0.18	0	0.43	wgt
0	3	LAD Stenosis	0.93	1	0.24	0.17	0	0.06	wgt
1	2	Age	66.91	66.2	9.53	0.08	0.13	0.06	wgt
1	2	Gender(Male)	0.8	0.79	0.4	0.03	0.63	0.01	wgt
1	2	Hypertension	0.59	0.68	0.49	0.17	0	0.08	wgt
1	2	Smoker	0.37	0.46	0.48	0.18	0	0.09	wgt
1	2	Hypercholesterolemia	0.45	0.54	0.5	0.18	0	0.09	wgt
1	2	Diabetes	0.25	0.24	0.43	0.03	0.69	0.01	wgt
1	2	Insulin Dependent Diabetes	0.1	0.09	0.29	0	0.95	0	wgt
1	2	COPD	0.07	0.07	0.25	0.01	0.85	0	wgt
1	2	AKD	0.04	0.03	0.2	0.04	0.25	0.01	wgt
1	2	Dialysis	0.01	0	0.09	0.04	0.01	0	wgt
1	2	Stroke History	0.03	0.05	0.17	0.1	0.12	0.02	wgt
1	2	TIA History	0.03	0.02	0.17	0.09	0	0.02	wgt
1	2	Carotid Stenosis > 50%	0.09	0.13	0.28	0.14	0.01	0.04	wgt
1	2	PAD	0.17	0.2	0.38	0.07	0.2	0.03	wgt
1	2	Pulmonary Hypertension	0.05	0.05	0.22	0.01	0.76	0	wgt
1	2	IABP Preop	0.02	0	0.12	0.1	0	0.01	wgt
1	2	LVEF	52.42	51.69	11.38	0.06	0.26	0.08	wgt
1	2	LMCA Stenosis	0.33	0.35	0.47	0.03	0.55	0.02	wgt
1	2	2-Vessel Disease	0.34	0.29	0.47	0.1	0.13	0.05	wgt

1	2	Previous PTCA	0.32	0.28	0.46	0.1	0.09	0.04	wgt
1	2	Antiplatelet Drugs	0.32	0.32	0.46	0.01	0.88	0	wgt
1	2	Surgery	0.09	0.1	0.28	0.03	0.52	0.01	wgt
1	2	REDO	0.04	0.06	0.19	0.11	0.07	0.02	wgt
1	2	NYHA ≥ 3	0.15	0.11	0.36	0.11	0.04	0.04	wgt
1	2	≥ 3-Vessel Disease	0.62	0.7	0.49	0.17	0.01	0.08	wgt
1	2	LAD Stenosis	0.94	0.95	0.24	0.04	0.24	0.01	wgt
1	3	Age	66.91	65.68	9.53	0.13	0.72	0.12	wgt
1	3	Gender(Male)	0.8	0.93	0.4	0.14	0	0.14	wgt
1	3	Hypertension	0.59	0.86	0.49	0.13	0	0.26	wgt
1	3	Smoker	0.37	0.65	0.48	0.18	0.03	0.28	wgt
1	3	Hypercholesterolemia	0.45	0.79	0.5	0.19	0	0.34	wgt
1	3	Diabetes	0.25	0.29	0.43	0.09	0.74	0.04	wgt
1	3	Insulin Dependent Diabetes	0.1	0.2	0.29	0.14	0.35	0.1	wgt
1	3	COPD	0.07	0.01	0.25	0.12	0	0.05	wgt
1	3	AKD	0.04	0.04	0.2	0	0.98	0	wgt
1	3	Dialysis	0.01	0	0.09	0.05	0.25	0	wgt
1	3	Stroke History	0.03	0.03	0.17	0	0.99	0	wgt
1	3	TIA History	0.03	0	0.17	0.15	0	0.03	wgt
1	3	Carotid Stenosis > 50%	0.09	0.11	0.28	0.1	0.63	0.03	wgt
1	3	PAD	0.17	0.15	0.38	0.05	0.78	0.02	wgt
1	3	Pulmonary Hypertension	0.05	0.05	0.22	0.02	0.87	0	wgt
1	3	IABP Preop	0.02	0	0.12	0.12	0	0.02	wgt
1	3	LVEF	52.42	51.14	11.38	0.11	0.33	0.27	wgt
1	3	LMCA Stenosis	0.33	0.26	0.47	0.16	0.44	0.08	wgt
1	3	2-Vessel Disease	0.34	0.09	0.47	0.13	0	0.25	wgt
1	3	Previuos PTCA	0.32	0.41	0.46	0.19	0.6	0.09	wgt
1	3	Antiplatelet Drugs	0.32	0.24	0.46	0.17	0.38	0.08	wgt
1	3	Surgery	0.09	0	0.28	0.15	0	0.09	wgt
1	3	REDO	0.04	0	0.19	0.18	0	0.03	wgt
1	3	NYHA ≥ 3	0.15	0.09	0.36	0.16	0.28	0.06	wgt
1	3	≥ 3-Vessel Disease	0.62	1	0.49	0.18	0	0.38	wgt

1	3	LAD Stenosis	0.94	1	0.24	0.13	0	0.06	wgt
2	3	Age	66.2	65.68	9.53	0.05	0.88	0.14	wgt
2	3	Gender(Male)	0.79	0.93	0.4	0.17	0	0.15	wgt
2	3	Hypertension	0.68	0.86	0.49	0.16	0.04	0.18	wgt
2	3	Smoker	0.46	0.65	0.48	0.17	0.14	0.19	wgt
2	3	Hypercholesterolemia	0.54	0.79	0.5	0.16	0.01	0.25	wgt
2	3	Diabetes	0.24	0.29	0.43	0.12	0.68	0.05	wgt
2	3	Insulin Dependent Diabetes	0.09	0.2	0.29	0.15	0.35	0.1	wgt
2	3	COPD	0.07	0.01	0.25	0.11	0	0.05	wgt
2	3	AKD	0.03	0.04	0.2	0.05	0.81	0.01	wgt
2	3	Dialysis	0	0	0.09	0.01	0.91	0	wgt
2	3	Stroke History	0.05	0.03	0.17	0.1	0.44	0.02	wgt
2	3	TIA History	0.02	0	0.17	0.07	0.04	0.01	wgt
2	3	Carotid Stenosis > 50%	0.13	0.11	0.28	0.04	0.84	0.01	wgt
2	3	PAD	0.2	0.15	0.38	0.12	0.53	0.05	wgt
2	3	Pulmonary Hypertension	0.05	0.05	0.22	0.03	0.81	0.01	wgt
2	3	IABP Preop	0	0	0.12	0.02	0.08	0	wgt
2	3	LVEF	51.69	51.14	11.38	0.05	0.71	0.19	wgt
2	3	LMCA Stenosis	0.35	0.26	0.47	0.19	0.37	0.09	wgt
2	3	2-Vessel Disease	0.29	0.09	0.47	0.14	0	0.21	wgt
2	3	Previous PTCA	0.28	0.41	0.46	0.19	0.44	0.13	wgt
2	3	Antiplatelet Drugs	0.32	0.24	0.46	0.18	0.38	0.08	wgt
2	3	Surgery	0.1	0	0.28	0.15	0	0.1	wgt
2	3	REDO	0.06	0	0.19	0.19	0	0.06	wgt
2	3	NYHA ≥ 3	0.11	0.09	0.36	0.05	0.75	0.02	wgt
2	3	≥ 3-Vessel Disease	0.7	1	0.49	0.17	0	0.3	wgt
2	3	LAD Stenosis	0.95	1	0.24	0.19	0	0.05	wgt

Abbreviations: tmt1: Treatment 1, tmt2: Treatmen 2, var: Variables, pop.sd: Standard Deviation Beetwen Treatments, Std.eff.sz: Standardized Effect Size, ks: Kolmogorov–Smirnov test, COPD: Chronic Obstructive Pulmonary Disease; AKD: Acute Kidney Disease; TIA: Transient Ischemic Attack; PAD: Peripheral Arterial Disease; IABP: Intra-Aortic Balloon Pump; NYHA: New York Heart Association Score; LVEF: Left Ventricular Ejection Fraction; LMCA: Left Main Coronary Artery; PTCA: Percutaneous Transluminal Coronary Angioplasty; unw: unweighte; wgt: weighted.

4.Estimate Tables

Table S17. Model 1. Stroke by Surgical technique

	Estimate	Lower 95% CI	Upper 95% CI
**NPA-OFF**	0.005	0.003	0.006
**SBCL-OFF**	0.020	0.018	0.022
**NPA-BHON**	0.009	0.006	0.011
**SBCL-BHON**	0.024	0.021	0.029
**NPA-TAC-ON**	0.060	0.057	0.062
**Single TAC-ON**	0.070	0.067	0.072
**TAC-SBCL-ON**	0.075	0.072	0.078

Abbreviations: CI: Confidence Interval; NPA-OFF: No Proximal Anastomoses Off-Pump; SBCL-OFF: Side Biting Clamp Off-Pump; NPA-BHON: No Proximal Anastomoses Beating Heart On-Pump; SBCL-BHON: Side Biting Clamp Beating Heart On-Pump; NPA-TAC-ON: No Proximal Anastomoses Aortic Total Clamp On-Pump; TAC-ON: Aortic Total Clamp On-Pump; TAC-SBCL-ON: Aortic Total Clamp Side Biting Clamp On-Pump.

Table S18. Model 2. Stroke by Number of Aortic Touches

	Estimate	Lower 95% CI	Upper 95% CI
**No-Touch**	0.005	0.001	0.009
**1-Touch**	0.013	0.011	0.014
**2-Touch**	0.029	0.026	0.031
**3-Touch**	0.068	0.064	0.071
**4-Touch**	0.085	0.082	0.087

Abbreviations: CI: Confidence Interval; TAC: Aortic Total Clamp.

Table S19. Model 3. Stroke by Cardiopulmonary Bypass

	Estimate	Lower 95% CI	Upper 95% CI
Off	0.012	0.008	0.015
On	0.042	0.024	0.059
On-Pump with Single TAC	0.068	0.050	0.085
On-Pump without Single TAC	0.018	0.012	0.023

Abbreviations: CI: Confidence Interval; TAC: Aortic Total Clamp.

Table S20. Model 4. Stroke by Single Total Aortic Clamp

	Estimate	Lower 95% CI	Upper 95% CI
No	0.014	0.010	0.017
Yes	0.070	0.060	0.079
TAC	0.070	0.068	0.071
TAC-NPA	0.060	0.050	0.069
TAC-SBCL	0.075	0.061	0.088

Abbreviations: CI: Confidence Interval; TAC: Aortic Total Clamp; NPA: No Prox. Anastomoses; SBCL: Side Biting Clamp.

Table S21. Model 5. Stroke by Side-Biting Clamp

	Estimate	Lower 95% CI	Upper 95% CI
No	0.026	0.022	0.029
Yes	0.039	0.033	0.044
SBCL OFF-Pump	0.026	0.022	0.029
SBCL ON-Pump	0.052	0.044	0.059
SBCL without TAC	0.023	0.019	0.026
SBCL with TAC	0.076	0.068	0.083

Abbreviations: CI: Confidence Interval; SBCL: Side Biting Clamp; TAC: Aortic Total Clamp.

Table S22. Model 6. Stroke by Proximal Anastomoses

	Estimate	Lower 95% CI	Upper 95% CI
No Prox Anast.	0.026	0.020	0.031
Yes	0.041	0.035	0.046
1 Prox Anast.	0.042	0.040	0.043
2 Prox Anast.	0.043	0.040	0.045
≥ 3 Prox Anast.	0.044	0.035	0.047

Abbreviations: CI: Confidence Interval.

5. Stroke Estimate: p values two-by-two comparisons

Table S23. Two-by-two comparisons by Surgical Technique

	NPA-OFF	SBCL-OFF	NPA-BHON	SBCL-BHON	NPA-TAC-ON	Single TAC-ON	TAC-SBCL-ON
**NPA-OFF**		0.091	0.71	0.062	0.01	< 0.001	< 0.001
**SBCL-OFF**			0.084	0.6	0.023	0.020	0.015
**NPA-BHON**				0.070	0.028	< 0.001	< 0.001
**SBCL-BHON**					0.027	0.023	0.015
**NPA-TAC-ON**						0.05	0.051
**Single TAC-ON**							0.4

Abbreviations: NPA-OFF: No Proximal Anastomoses Off-Pump; SBCL-OFF: Side Biting Clamp Off-Pump; NPA-BHON: No Proximal Anastomoses Beating Heart On-Pump; SBCL-BHON: Side Biting Clamp Beating Heart On-Pump; NPA-TAC-ON: No Proximal Anastomoses Aortic Total Clamp On-Pump; TAC-ON: Aortic Total Clamp On-Pump; TAC-SBCL-ON: Aortic Total Clamp Side Biting Clamp On-Pump.

S24. Two-by-two comparisons by Number of Aortic Touches

	1-Touch	2-Touch	3-Touch	4-Touch
**No-Touch**	0.58	0.052	< 0.001	< 0.001
**1-Touch**		0.055	0.020	< 0.001
**2-Touch**			0.022	0.017
**3-Touch**				0.055
**3-Touch NPA**			0.62	0.35

Abbreviations: TAC: Aortic Total Clamp.

S25. Two-by-two comparisons by Cardiopulmonary Bypass (CPB)

	On-Pump	On-Pump with TAC	On-Pump without TAC
**Off-Pump**	0.026	0.01	0.6

Abbreviations: TAC: Aortic Total Clamp.

S26. Two-by-two comparisons by Cardiopulmonary Bypass (CPB) and by Total Aortic Clamp

	TAC	TAC-NPA	TAC+SBCL
**No TAC**	0.01		
**TAC**		0.057	0.4

Abbreviations: TAC: Aortic Total Clamp; NPA: No Prox Anastomoses; SBCL: Side Biting Clamp.

S27. Two-by-two comparisons by the use of Side-Biting Clamp

	SBCL	SBCL ON-Pump	SBCL with TAC
**No SBCL**	0.052		
**SBCL OFF-Pump**		0.06	
**SBCL without TAC**			0.018

Abbreviations: SBCL: Side Biting Clamp; TAC: Aortic Total Clamp.

S28. Two-by-two comparisons by Creation of Proximal Anastomoses

	Prox Anast	1 Prox Anast	2 Prox Anast	Prox Anast ≥ 3
No Prox Anast	0.055			
1 Prox Anast			0.8	0.7
2 Prox Anast				0.8
Prox Anast ≥ 3				

6. Statistics Notes

The multinomial propensity score[1], implemented for multiple treatments, was created for each model[2-4]. Inverse Probability of Treatment Weighting (IPTW) was used to correct for imbalances between groups on pre-treatment covariates so that the distribution of the pre-treatment characteristics would be similar across all the groups. A machine learning technique, generalized boosted model (GBM), was used to estimate the PS weights. GBM estimation captures complex relationships between treatment assignment and pre-treatment variables without over-fitting data[3], and GBM can be fine-tuned to find the best balance among groups.

IPTW is one technique for reducing the bias due to observed variables. It relies on two key conditions for obtaining unbiased estimates [5]: 1) No unknown or unmeasured confounders assumption or exchangeability and 2) Sufficient overlap or positivity: $0 < \Pr(T_i = t|\mathbf{X}) < 1$, for all **X** and *t*, where T_i is the random treatment assignment variable, Pr is probability, X is the vector of observed treatment covariates and t is the treatment. The first assumption states that the set of observed variables is rich enough to include all variables influencing both treatments and outcomes. The second condition states that each patient has a non-zero probability of receiving each treatment. Both assumptions were met in our models.

For our binary outcome (ischemic stroke) many simple regression trees were generated starting from a single regression tree and adding another tree at each new iteration to create an overall piecewise constant function[6]. This iterative fitting algorithm was chosen so as to provide the best fit to the residuals of the model from the previous iteration and because it offers the greatest increase to the log likelihood for the data. Indeed, each iteration increases the likelihood making the model sufficiently flexible to perfectly fit data. To avoid data overfitting, GBM selects an intermediate iteration (or number of trees) for the final model so as to "minimize an external criterion such as out-of-sample prediction error or — in the case of propensity score estimation — imbalance on the pre-treatment covariates across the treatment and control groups"[3,4]. Therefore, the key is to use GBM iteratively with the optimal iteration (number of trees) for estimating the PS and minimizing a

"stopping rule" criterion based on the difference between the weighted distributions of the pre-treatment variables in the two treatment conditions. In practice, different stopping rules have been used to select the optimal iteration of GBM for use in estimating propensity score weights: maximum or minimum absolute standardized bias (SB, also referred to as the absolute standardized mean difference) or the Kolmogorov-Smirnov (KS) statistic, each of which compares the means or the distributions of the covariates between treatment groups. Since the balance was nearly invariant with the stopping rule, we used the max Kolmogorov-Smirnov (KS) statistic.

For 2 groups the KS is:

$KS_k = sup_x |EDF_{1k}(x) – EDF_{0k}(x)|$

where EDF is the empirical distribution function for the treatment and control samples and k is the covariate.

For multiple treatments this function was modified as suggested by McCaffrey[4]:

$PKS_{tk} = sup_x |EDF_{tk}(x) – EDF_{pk}(x)|$

where EDF is the empirical distribution function for the pooled sample across all treatments, and t and p are the treatments under consideration.

Causal effects can be estimated through two different summaries: average treatment effect (ATE) and average treatment effect between treatments (ATT). The ATE of treatment t_i versus treatment t_j is the comparison of mean outcome had the entire population been observed under treatment t_i versus had the entire population been observed under treatment t_i [7].The ATT of t_i versus t_j is the comparison of the mean "t_i" patient outcome with the mean outcome they would have had if they had instead been treated by t_j treatment[7].

Since we could not observe a patient under each of the seven treatments, the casual effect was estimated by ATE which takes into account summary statistics of the effects across populations of interest.[8]

As we were interested in estimating ATE, it was necessary to estimate the population mean of the stroke for each of the treatments to obtain estimates of the desired causal effect. Let $p_t(\mathbf{X})$ be the PS,

and let the probability that an individual with pre-treatment variables **X** receives treatment be t(p_t (**X**) = Pr(T[t =1 | **X**). A consistent estimate of stroke (e[μ_t]) is given by the weighted mean

$$e[\mu_t] = \frac{\sum_{1=1}^{n} T_i[t] Y_i w_i[t]}{\sum_{1=1}^{n} T_i[t] w_i[t]}$$

where the weights are

$$w_i[t] = 1 / p_t(\mathbf{X_i})$$

R software v. 3.6.1 (R Foundation for Statistical Computing, Vienna, Austria) and the TWANG and SURVEY packages were used for analysis.

6.1 Advantages of the PS method over regression-based prediction

Regression-based, covariate-adjustment techniques correct for imbalances between groups on pre-treatment covariates.

Compared to these techniques, the theoretical advantages of PS are

1) In case of a large number of pretreatment covariates, PS summarizes all pretreatment variables to a single score thus being an advantageous reduction tool for evaluating treatment effects[9].
2) Using PS causal questions can be well-defined and explicitly specified and is not combined with the modeling approach as occurs in traditional regression methods.
3) PS methods allow us to avoid bias for model misspecification since they do not model the mean[10].
4) Differently from regression modelling, PS methods do not extrapolate beyond the observed data[11].
5) Most importantly, PS methods eliminate the potential bias of choosing the model specification for pretreatment variables thus influencing its impact on the estimated treatment effect[12].

6.2 Pretreatment variables

The pretreatment variables included in the models were: Age, Gender (Male), Hypertension, Smoke, Hypercholesterolemia, Diabetes, Insulin Dependent Diabetes, Chronic Obstructive Pulmonary Disease (COPD), Acute Kidney Disease (AKD), Dialysis, Stroke, Transient Ischemic Attack (TIA), Carotid stenosis > 50%, Peripheral Arterial Disease (PAD), Pulmonary Hypertension, Intra-Aortic Balloon Pump (IABP), NYHA ≥ 3, Left Ventricle Ejection Fraction (LVEF), LMCA Stenosis, LAD Stenosis, Two-vessel disease, Three-vessel disease, Previous PTCA, Antiplatelet Drugs, REDO.

6.3 Variables Included in the Propensity Score Models

Before weighting, the following pretreatment variables were chosen to balance patients, taking into account that the set was sufficiently rich to include all variables influencing observed treatments and outcomes: Age, Gender (Male), Hypertension, Smoke, Hypercholesterolemia, Diabetes, Insulin Dependent Diabetes, COPD, AKD, Dialysis, Stroke, TIA, Carotid Stenosis > 50%, PAD, Pulmonary Hypertension, IABP, NYHA ≥ 3, LVEF, LMCA Stenosis, LAD Stenosis, Two-vessel disease, Three-vessel disease, Previous PTCA, Antiplatelet Drugs, REDO.

The positivity assumption (each patient has a non-zero probability of receiving each treatment) was tested.

7. STROBE

Supplementary Table S29. Full guidelines for reporting propensity score analysis, modified From the STROBE (STrengthening the Reporting of Observational studies in Epidemiology) Statement[13-14].

Section/topic	Item No		Recommendation
**Title and abstract**	**x**	1	Indicate the use of propensity analysis with a commonly used term in the title or the abstract
	**x**	2	Provide in the abstract an informative and balanced summary of what was done and what was found
**Introduction**			
Background/rationale	**x**	3	Explain the scientific background and rationale for the investigation being reported
Objectives	**x**	4	State specific objectives, including any prespecified hypotheses
**Methods**			
Setting	**x**	5	Describe the setting, locations, and relevant dates, including periods of recruitment, treatment, follow-up, and data collection
Patient selection	**x**	6	Give the eligibility criteria, and the sources and methods of subject ascertainment and selection
Variables	**x**	7	Clearly define all outcomes, treatments, predictors. Give diagnostic criteria, if applicable
Data sources/ measurement	**x**	8	For each variable of interest, give sources of data and details of methods of assessment (measurement)
Bias	**x**	9	Describe how propensity score analysis was used to address bias
	**x**	10	Describe any other methods to address potential sources of bias, e.g. sensitivity analysis
Sample size	**x**	11	Explain how the study size was arrived at
Statistical analyses	**x**	12	Describe all the analytic methods, including the propensity score methods, e.g. matching, weighting, stratification, or covariate adjustment using propensity score
	**x**	13	Indicate the model used to estimate propensity score, e.g. logistic model, boosting (meta-classifiers), decision trees
	**x**	14	State the variables included in the propensity score model
	**x**	15	Explain the variable selection procedure for propensity score model
		16	For propensity score matching:
	**NA**	16.1	Explicitly state the matching algorithm and distance metric
	**NA**	16.2	Indicate matching ratio (1:m matching)

	**NA**	16.3	Indicate whether sampling with or without replacement was used
	**NA**	16.4	Describe the statistical methods for the analysis of matched data
	**NA**	16.5	Describe methods for assessing the comparability of baseline characteristics in the matched groups
	**x**	17	For propensity score weighting, describe methods for assessing the comparability of baseline characteristics in the weighted groups
		18	For propensity score stratification:
	**NA**	18.1	Give the number of strata
	**NA**	18.2	Describe methods for assessing the comparability of baseline characteristics in each stratum
	**x**	19	Explain how assumption of propensity score analysis was examined
	**x**	20	Explain how missing data were addressed, including missing data in propensity score estimation
	**x**	21	If applicable, describe any methods used to examine subgroups and interactions
	**x**	22	Describe any sensitivity analyses
	**x**	23	Indicate the software used for analysis
	**x**	24	If applicable, report the package used to create matched sample, e.g. GMATCH macro in SAS, MatchIt package®, Optmatch package ®
**Results**			
Participants		25	Report numbers of participants at each stage of study:
	**x**	25.1	sample size of patients potentially eligible
	**x**	25.2	sample size of patients confirmed eligible and included
	**x**	25.3	sample size of patients analyzed
	**x**	25.4	for propensity score matching, sample size for each treatment group before and after matching
	**x**	26	Explain reasons for exclusion at each stage
	**NA**	27	Consider use of a flow diagram
Patient characteristics	**x**	28	Describe the distribution of baseline characteristics for each group before propensity score analysis
		29	For propensity score matching, weighting, or stratification:
	**x**	29.1	Describe the distribution of baseline characteristics in the matched/weighted groups or in each stratum
	**x**	29.2	Describe the results of the comparability of baseline characteristics, whether there are still systematic differences between treatment groups
	**NA**	30	Indicate number of patients with missing data for each variable of interest, especially the variables used

			in propensity score model
Outcome data	**x**	31	Report outcomes of each treatment group
Main results	**x**	32	Give propensity score analysis estimates and their precision, e.g. 95% confidence interval
	**x**	33	If applicable, give unadjusted estimates and/or adjusted estimates and their precision, e.g. 95% confidence interval. Make clear which additional factors were adjusted for
Other analyses	**x**	34	Report other analyses done, e.g. analyses of subgroups and interactions, and sensitivity analyses
**Discussion**			
Key results	**x**	35	Summarize key results with reference to study objectives
Limitations	**x**	36	Discuss limitations of the study, taking into account sources of potential bias or imprecision
	**x**	37	Discuss both direction and magnitude of any potential bias
Interpretation	**x**	38	Discuss whether imbalance of baseline characteristics still exists, and give a cautious interpretation
	**x**	39	Give a cautious overall interpretation of results considering objectives, limitations, multiplicity of analyses, results from similar studies, and other relevant evidence
Generalizability	**NA**	40	For propensity score matching, discuss the possibility and potential influence of incomplete matching, especially the studies in which the matched sample size is less than 50%
**Other information**			
Funding	**x**	41	Give the source of funding and the role of the funders for the present study and, if applicable, for the original study on which the present article is based

Author roles

This study was designed by Prof. Gelsomino. Data were acquired by Drs. Stefano del Pace, Prof. Bonacchi, and Prof. Prifti and were analyzed by Drs. Orlando Parise and Francesco Matteucci. All authors contributed to the interpretation of the data, vouch for the data and analysis, contributed to the writing of the manuscript, and agreed to publish this study. Prof. Gelsomino, Prof. LaMeir, and Prof. Bonacchi authored first drafts of parts of the manuscript that were subsequently combined. Prof. Edvin Prifti served as cardiac surgeon at Careggi University Hospital of Florence. Now he is Chief of Cardiac Surgery at Tirana Hospital, Tirana, Albania. Prof. Sandro Gelsomino was cardiac surgeon at Careggi University Hospital of Florence. Now he is Chair of Cardiac Surgery at University of Maastricht.

7.1 Database

A specific database was created by Sandro Gelsomino, Stefano del Pace, Edvin Prifti, and Massimo Bonacchi. The database included clinical and demographic characteristics, perioperative and operative data. Information regarding patients who were transferred after surgery to a companion hospital was directly obtained by collegues. Training students at the Cardiology School of the University of Florence took care of filling in data under the direct supervision of one of the main authors and senior researchers. The database was transferred from the original excel file to R for statistical analysis.

8. Ethical Issues

Ethical Committee approval was waived due to the retrospective analysis of the study according to national laws regulating observational retrospective studies (Italian law nr.11960, released on 13/07/2004). All patients gave their consent for the use of data for scientific purposes.

References:

1. Harder VS, Stuart EA, Anthony JC. Propensity score techniques and the assessment of measured covariate balance to test causal associations in psychological research. Psychol Methods 2010;15:234-49.
2. Pirracchio R, Petersen ML, van der Laan M. Improving propensity score estimators' robustness to model misspecification using super learner. American journal of epidemiology 2015;181:108-19.
3. McCaffrey DF, Ridgeway G, Morral AR. Propensity score estimation with boosted regression for evaluating causal effects in observational studies. Psychol Methods 2004;9:403-25.
4. McCaffrey DF, Griffin BA, Almirall D, Slaughter ME, Ramchand R, Burgette LF. A tutorial on propensity score estimation for multiple treatments using generalized boosted models. Stat Med 2013;32:3388-414.
5. Robins JM, Hernan MA, Brumback B. Marginal structural models and causal inference in epidemiology. Epidemiology 2000;11:550-60.
6. Breiman L, Friedman JH, Olshen RA, Stone CJ. Classification and Regression Tree. 1983.
7. Wooldridge JM. Econometric analysis of cross section and panel data MIT Press. Cambridge, MA 2002;108.
8. Holland PW. Statistics and Causal Inference. Journal of the American Statistical Association 1986;81:945-60.
9. Rosenbaum PR, Rubin DB. Reducing Bias in Observational Studies Using Subclassification on the Propensity Score. Journal of the American Statistical Association 1984;79:516-24.
10. Ho DE, Imai K, King G, Stuart EA. MTAChing as Nonparametric Preprocessing for Reducing Model Dependence in Parametric Causal Inference. Political Analysis 2007;15:199-236.
11. Stuart EA. MTAChing methods for causal inference: A review and a look forward. Stat Sci 2010;25:1-21.
12. Rubin DB. Using Propensity Scores to Help Design Observational Studies: Application to the Tobacco Litigation. Health Services and Outcomes Research Methodology 2001;2:169-88.
13. von Elm E, Altman DG, Egger M, et al. The Strengthening the Reporting of Observational Studies in Epidemiology (STROBE) statement: guidelines for reporting observational studies. J Clin Epidemiol 2008;61(4):344-9.
14. Yao, X. I. *et al.* Reporting and Guidelines in Propensity Score Analysis: A Systematic Review of Cancer and Cancer Surgical Studies. *J Natl Cancer Inst* **109**, djw323, doi:10.1093/jnci/djw323 (2017
