# Supplementary material for: Surgical Risk Factors for Ischemic Stroke Following Coronary Artery Bypass Grafting. A Multi-Factor Multimodel Analysis
Source: Front Cardiovasc Med. 2021 Jul 5;8:622480. doi: 10.3389/fcvm.2021.622480 (PMC8287035; doi:10.3389/fcvm.2021.622480)
Supplement: Supplementary file 2 [file Table_1.DOCX]

NUMEROSITY of SUB-GROUPS

**By Touch:**

no-touch: 1,062

1-touch: 2,634

2-touch: 1,561

3-touch: 4,907

4-touch: 6,091

**By Proximal Anastomoses:**

0: 2,839

1: 12,155

2: 1,210

3: 51

**By Side Clamp:**

No: 6,809

Yes: 9,446

**By Needle:**

No: 5,257

Yes: 10,998

**By Cannula:**

No: 2,856

Yes: 13,399
